# Global, regional, and national esophageal cancer deaths and DALYs attributable to diet low in vegetables and fruits, 1990–2019: analysis for the global burden of disease study

**DOI:** 10.3389/fnut.2024.1478325

**Published:** 2025-01-07

**Authors:** Bing Cui, Aqin Chen, Chengcheng Xu

**Affiliations:** ^1^Department of Blood Transfusion, Affiliated Hospital of Jiangsu University, Zhenjiang, Jiangsu Province, China; ^2^Department of Nuclear Medicine, Affiliated Hospital of Jiangsu University, Zhenjiang, Jiangsu Province, China

**Keywords:** esophageal cancer, diet, vegetables, fruits, disease burden

## Abstract

**Background:**

This study aimed to comprehensively assess the global, regional, and national burden of esophageal cancer (EC) attributable to inadequate vegetable and fruit intake from 1990 to 2019 and explore the potential impact of existing dietary intervention programs on EC prevention.

**Methods:**

Using the Global Burden of Disease Study 2019 (GBD 2019) database, we conducted descriptive analyses stratified by age, sex, Socio-demographic Index (SDI), and regional levels. Temporal trends were assessed using linear regression models, and cluster analysis was employed to explore burden patterns across different GBD regions. Decomposition analysis quantified the contributions of aging, population dynamics, and epidemiological changes to deaths and disability-adjusted life years (DALYs). Frontier analysis was used to evaluate the relationship between dietary risk-related disease burden and sociodemographic progress.

**Results:**

In 2019, inadequate vegetable and fruit intake contributed to 65,919 global EC deaths, accounting for 0.12% of all deaths, with an age-standardized death rate of 0.81 per 100,000 population. The associated DALYs totaled 16,065,68, representing 0.06% of total global DALYs, with an age-standardized DALY rate of 19.24. The disease burden attributable to insufficient fruit intake (51,210 deaths, 12,497,75 DALYs) was significantly higher than that from inadequate vegetable intake (17,176 deaths, 4,203,09 DALYs). The burden was greater in males than females, peaking in middle-aged groups. Substantial regional differences were observed, with low-SDI regions bearing the highest burden. From 1990 to 2019, while the absolute numbers of deaths and DALYs followed a complex trajectory of initial increase followed by decline, age-standardized rates consistently decreased, reflecting the positive impact of epidemiological improvements. Existing dietary intervention programs, such as subsidies for fruit and vegetable production and health education initiatives, have contributed to a reduction in dietary risk-related disease burden but exhibited varying effectiveness across SDI regions.

**Conclusion:**

Targeted dietary interventions, such as promoting fruit and vegetable consumption, are critical for the prevention and control of the EC disease burden. Future efforts should focus on optimizing the implementation of current programs, enhancing nutritional supplementation in resource-limited regions, and expanding health education initiatives to achieve broader health benefits.

## 1 Introduction

Esophageal cancer (EC) represents a highly aggressive malignancy, exhibiting a rising incidence and an ominous prognosis (1). This malignancy stands out as a relatively common and lethal form of cancer, ranking as the sixth leading cause of cancer-related deaths globally (2). EC has emerged as a pressing global health concern, affecting numerous regions and carrying a significant burden of morbidity and mortality (3, 4). The etiology of EC is a complex tapestry of diverse factors, with dietary habits playing a pivotal role in its development (3, 5, 6). Specifically, a diet lacking in vegetables and fruits, which are rich repositories of micronutrients and antioxidants, has been widely recognized as a modifiable risk factor for EC (7, 8). These nutrients are vital for maintaining optimal health and preventing chronic diseases. A dietary deficiency in these nutrients can lead to nutritional imbalances, thereby compromising the body’s defensive mechanisms against carcinogenic agents (9).

Previous studies have established a clear association between a diet low in vegetables and fruits and the risk of EC (10, 11). However, a comprehensive analysis of the global, regional, and national burden of EC deaths and Disability-Adjusted Life Years (DALYs) attributable to this dietary pattern is still lacking. Such an analysis is crucial for understanding the scope of the problem, identifying high-risk regions and subpopulations, and guiding targeted intervention strategies.

The Global Burden of Disease (GBD) Study, a collaborative effort among multiple institutions worldwide, aims to quantify the health loss due to various diseases and injuries. Within this framework, the present study aims to estimate the burden of EC deaths and DALYs attributable to a diet low in vegetables and fruits. By doing so, we hope to provide valuable insights into the impact of this modifiable risk factor on global health and identify key areas for intervention. This will inform policies and programs aimed at promoting healthy dietary habits and reducing the burden of EC worldwide.

## 2 Methods

### 2.1 Data source

We utilized the Global Burden of Disease Study 2019 (GBD 2019) database as a primary source of epidemiological data. GBD 2019 is a comprehensive global health statistics database that assesses the burden of diseases, injuries, and risk factors, providing estimates for incidence, prevalence, mortality, and disability-adjusted life years (DALYs) across various conditions (12–15). DALYs, a metric for measuring the total disease burden, are composed of years of life lost due to premature mortality (YLLs) and years lived with disability (YLDs), calculated using the formula: DALYs = YLLs + YLDs. This enables a comprehensive evaluation of health loss caused by specific conditions. The database supports analysis by age, sex, and geographical region, facilitating assessments of temporal trends and the potential impacts of interventions. In this study, we additionally calculated the estimated annual percentage change (EAPC) using linear regression, with the formula: EAPC = (e^b^ − 1) × 100, where b represents the time regression coefficient. Positive and negative EAPC values indicate upward or downward trends in metrics, respectively. Furthermore, the Socio-demographic Index (SDI), a composite indicator reflecting development levels, ranging from 0 to 1, was utilized. SDI incorporates three key dimensions: per capita income, educational attainment, and total fertility rate. In this study, SDI was applied to classify regions and investigate the relationship between development levels and disease burden.

### 2.2 Description analysis

Based on the GBD 2019 database, we conducted a subgroup analysis of the disease burden data for 2019. The analysis stratified key indicators by age, sex, SDI, GBD regions, and countries, enabling a comparative assessment of the burden of EC attributable to inadequate vegetable and fruit intake. For each subgroup, we comprehensively evaluated deaths and DALYs, highlighting disparities in the EC burden linked to this dietary risk factor across age groups, sexes, regions, and countries.

### 2.3 Trend analysis

To investigate the trends in the global and regional burden of EC from 1990 to 2019, we used linear regression models to calculate the EAPC. Additionally, we employed hierarchical clustering analysis to explore the heterogeneity in dietary risk-related EC burden patterns across different GBD regions. Euclidean distance was used as the similarity measure, and the Ward method was applied to optimize clustering results. The GBD regions were classified into four distinct categories, revealing similarities and differences in disease burden patterns. This analysis grouped regions based on the similarity of trends in dietary risk-related EC burden, providing insights into the patterns of change across these areas.

### 2.4 Decomposition analysis

We applied decomposition analysis to evaluate the sources of changes in EC deaths and DALYs from 1990 to 2019, categorizing them into three key factors: aging (changes in population age structure), population dynamics (changes in population size), and epidemiological changes (age- and population size-adjusted changes in incidence or mortality rates) (16).

### 2.5 Frontier analysis

To examine the relationship between sociodemographic development levels and the burden of EC, we employed non-parametric Data Envelopment Analysis (DEA) to construct a health frontier, assessing the realization of health potential across different countries and regions. Health frontier analysis is a non-parametric method used to evaluate health performance in different regions at specific levels of sociodemographic development. Specifically, this analysis establishes a theoretical “optimal health outcome benchmark” and quantifies the gap between actual health outcomes and this benchmark in each region. This gap represents the unrealized health potential at the current level of development. For example, in some regions, optimizing dietary interventions could substantially reduce the EC burden attributable to insufficient fruit and vegetable intake. However, actual outcomes may fall short of expectations, highlighting potential areas for improvement in health outcomes. By identifying these unrealized potentials, health frontier analysis provides critical insights for public health policy-making, helping prioritize resource allocation and optimize intervention strategies (17, 18). All statistical analyses and graphical representations within this study were conducted and executed using the statistical software R (version 3.5.1).

## 3 Results

### 3.1 The disease burden of EC attributable to diet low in vegetables and fruits in 2019

In 2019, EC attributable to dietary risks resulted in 65,919 deaths [95% uncertainty intervals (UI): 26,635-121,827], accounting for 0.12% of deaths globally. Of these deaths, 17,176 (95% UI: 2,549-33,958) were attributed to diets low in vegetables, while 51,210 (95% UI: 15,227–108,734) were linked to diets low in fruits. The corresponding age-standardized deaths rate was 0.81 (95% UI: 0.33–1.50) per 100,000 population. Specifically, the rate for diet low in vegetables was 0.21 (95% UI: 0.03–0.42). And for diet low in fruits, it was 0.63 (95% UI: 0.19–1.33). Furthermore, the total number of DALYs cases associated with these dietary risks amounted to 16,065,68 (95% UI: 6,684,74-29,136,73), representing 0.06% of global DALYs in 2019. Among these, 4,203,09 (95% UI: 641,54–8,276,94) were attributed to diet low in vegetables, and 12,497,75 (95% UI: 3,844,70-25,950,57) were due to diet low in fruits. The corresponding ASR of DALYs was 19.24 (95% UI: 7.99–34.88) for EC attributable to dietary risks per 100,000 population. Specifically, the ASR for diet low in vegetables was 5.03 (95% UI: 0.77–9.91). And for diet low in fruits, it was 14.96 (95% UI: 4.60–31.05; Supplementary Tables S1–S6).

In 2019, males exhibited a significantly higher burden of dietary risks-related EC compared to females, with the number of deaths cases being 2.27 times greater and the number of DALYs cases being 2.45 times than in females. Similarly, the corresponding age-standardized rates (ASRs) were 2.63 times for deaths and 2.66 times for DALYs in males compared to females. This gender disparity was consistent across both diets low in vegetables and fruits (Supplementary Figure S1; Supplementary Tables S1–S6).

In Supplementary Figure S2, we presented the distribution of deaths and DALYs across different age groups in 2019 for EC attributable to dietary risks, diets low in vegetables, and diets low in fruits. The data revealed a distinct pattern: the number of deaths and DALYs cases initially rose with age, reaching a peak, and then subsequently declined. Notably, the ASRs exhibited a similar trend to the number of cases, further supporting this observed pattern (Supplementary Figure S2; Supplementary Tables S1–S6).

At the level of the SDI regions, the ASRs for deaths and DALYs associated with dietary risks-related EC exhibited an inverse relationship with the SDI, indicating that the rates were highest in low SDI regions. However, the number of deaths and DALYs cases initially increased as the SDI decreased, peaking at a certain point, and then subsequently declined. Consequently, the burden of disease was greatest in middle SDI regions. For EC attributable to diets low in fruits, the disease burden across different SDI regions followed a similar pattern to that of dietary risks-related EC. When considering EC related to diets low in vegetables, the trend differed. The ASRs for deaths and DALYs displayed a “U-shaped” relationship with decreasing SDI, with the burden being lowest in high-middle SDI regions and highest in low SDI regions. The number of deaths and DALYs cases associated with EC attributable to diets low in vegetables exhibited a more complex “S-shaped” relationship with decreasing SDI. Specifically, the numbers initially decreased, reaching their lowest point in high-middle SDI regions, then increased to their highest level in low-middle SDI regions, and finally continued to decrease (Supplementary Figure S3; Supplementary Tables S1–S6).

Across the 45 GBD regions and 204 countries studied, we observed substantial variation in the disease burden. A detailed visualization of these disparities was shown in Supplementary Figures S4–S7 and Supplementary Tables S1–S6.

### 3.2 Temporal trend for the disease burden of EC attributable to diet low in vegetables and fruits from 1990 to 2019

**Figure 1:**
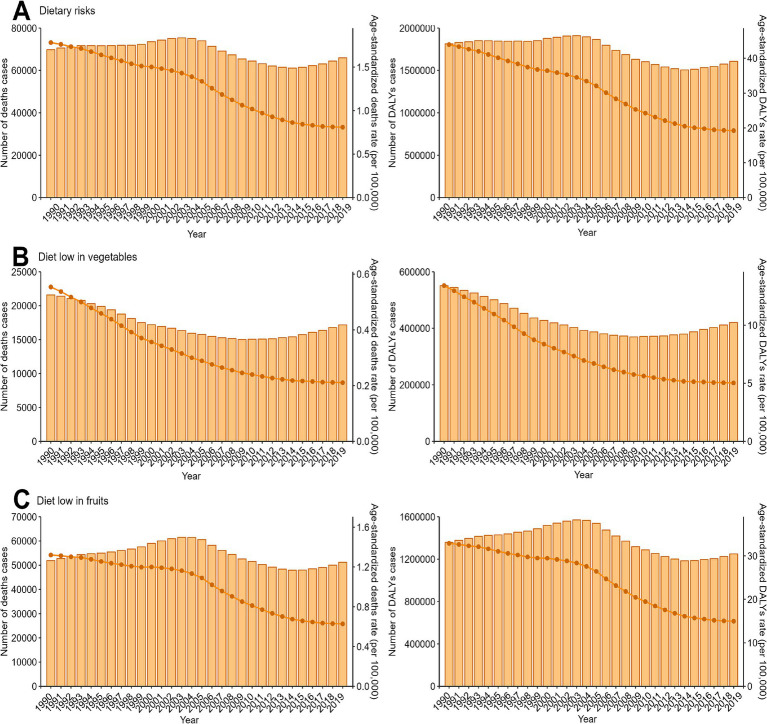
Trends in the numbers and age-standardized rates of esophageal cancer attributable to diet low in vegetables and fruits deaths and DALYs globallyfrom 1990 to 2019. DALYs, disability-adjusted-life-years. **(A)** Dietary risks, Global Trends of Deaths and DALYs Attributable to Dietary Risks (1990–2019). **(B)** Diet low in vegetables, Global Trends of Deaths and DALYs Attributable to Low Vegetable Intake (1990–2019). **(C)** Diet low in fruits, Global Trends of Deaths and DALYs Attributable to Low Fruit Intake (1990–2019).

From 1990 to 2019, the number of deaths and DALYs cases of EC attributable to dietary risks initially increased, followed by a decrease. However, in recent years, these numbers have begun to rise again. However, the age-standardized rates exhibited a decreasing trend, with EAPC values of −3.08 (95% UI: −3.3–2.86) for deaths and − 3.22 (95% UI: −3.44–3) for DALYs. Notably, the trends observed for EC associated with a low intake of fruits mirrored those linked to overall dietary risks. In contrast, the trend in the disease burden of EC attributed to a diet low in vegetables differed. Specifically, the number of deaths and DALYs cases decreased initially but subsequently increased (Figure 1; Supplementary Tables S1–S6).

The trends observed in males and females, respectively, aligned with those of the overall population, as shown in Supplementary Figure S8 and detailed in Supplementary Tables S1–S6. Furthermore, these trends were consistent across most age groups, as illustrated in Supplementary Figure S9 and corroborated by the data presented in Supplementary Tables S1–S6.

At the regional level of the SDI, the trend observed for ASRs of deaths and DALYs of EC attributable to dietary risks including low vegetable and fruit intake, mirrored the overall trend. Nevertheless, in terms of the absolute number of deaths and DALYs cases, regions categorized as high SDI, low-middle SDI, and low SDI exhibited a consistent upward trend, regardless of the specific dietary risk factor, namely diets low in vegetables or fruits. Conversely, the pattern in high-middle SDI and middle SDI regions followed the same trend as the overall total for deaths and DALYs cases (Supplementary Figure S10; Supplementary Tables S1–S6).

**Figure 2:**
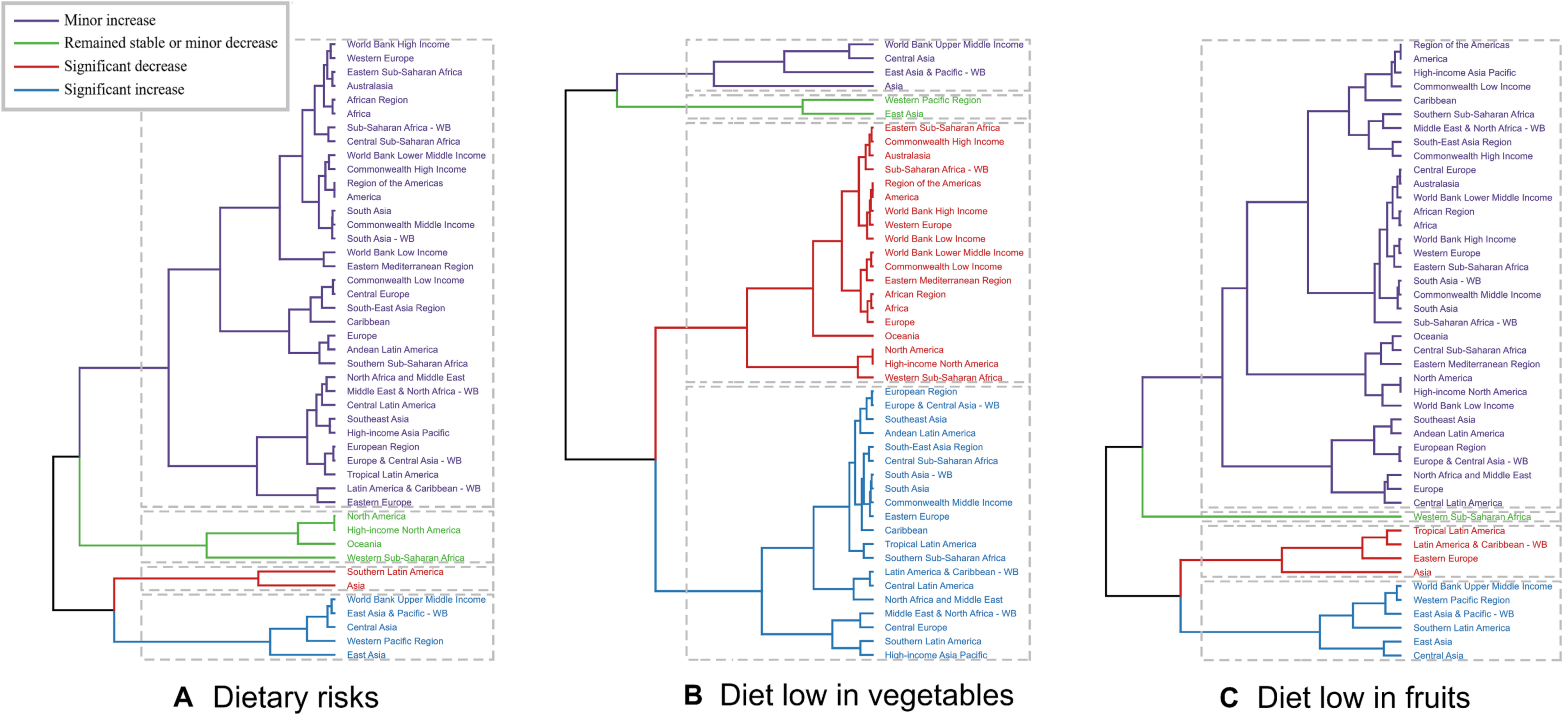
Results of cluster analysis based on the EAPC values of the age-standardized deaths and DALYs rates of esophageal cancer attributable to diet low in vegetables and fruits from 1990 to 2019. EAPC, estimated annual percentage change; DALYs, disability-adjusted-life-years. **(A)** Dietary risks: South Asia and Central Asia show significant decreases, while East Asia and Pacific regions show significant increases. **(B)** Diet low in vegetables: Eastern Europe and Tropical Latin America have significant increases, whereas South Asia and Central Asia show decreases. **(C)** Diet low in fruits: Significant decreases in East Asia and Southeast Asia, but increases in Eastern Europe and Tropical Latin America.

Across the various GBD regions, significant differences in the burden of EC attributable to diets low in vegetables and fruits were observed. To identify regions exhibiting similar patterns of variation in disease burden, a hierarchical clustering analysis was performed in this study. The findings of this analysis are presented in Figure 2. For EC attributable to dietary risks, a notable increase in both the death rate and DALYs rate was evident in regions such as the World Bank Upper Middle Income, East Asia & Pacific - WB, Central Asia, Western Pacific Region, and East Asia. Conversely, a significant decrease was observed in Southern Latin America and Asia. Regarding the more specific dietary risks of diet low in vegetables and fruits, the clustering analysis results are displayed in Figure 2 (Supplementary Tables S1–S6).

**Figure 3:**
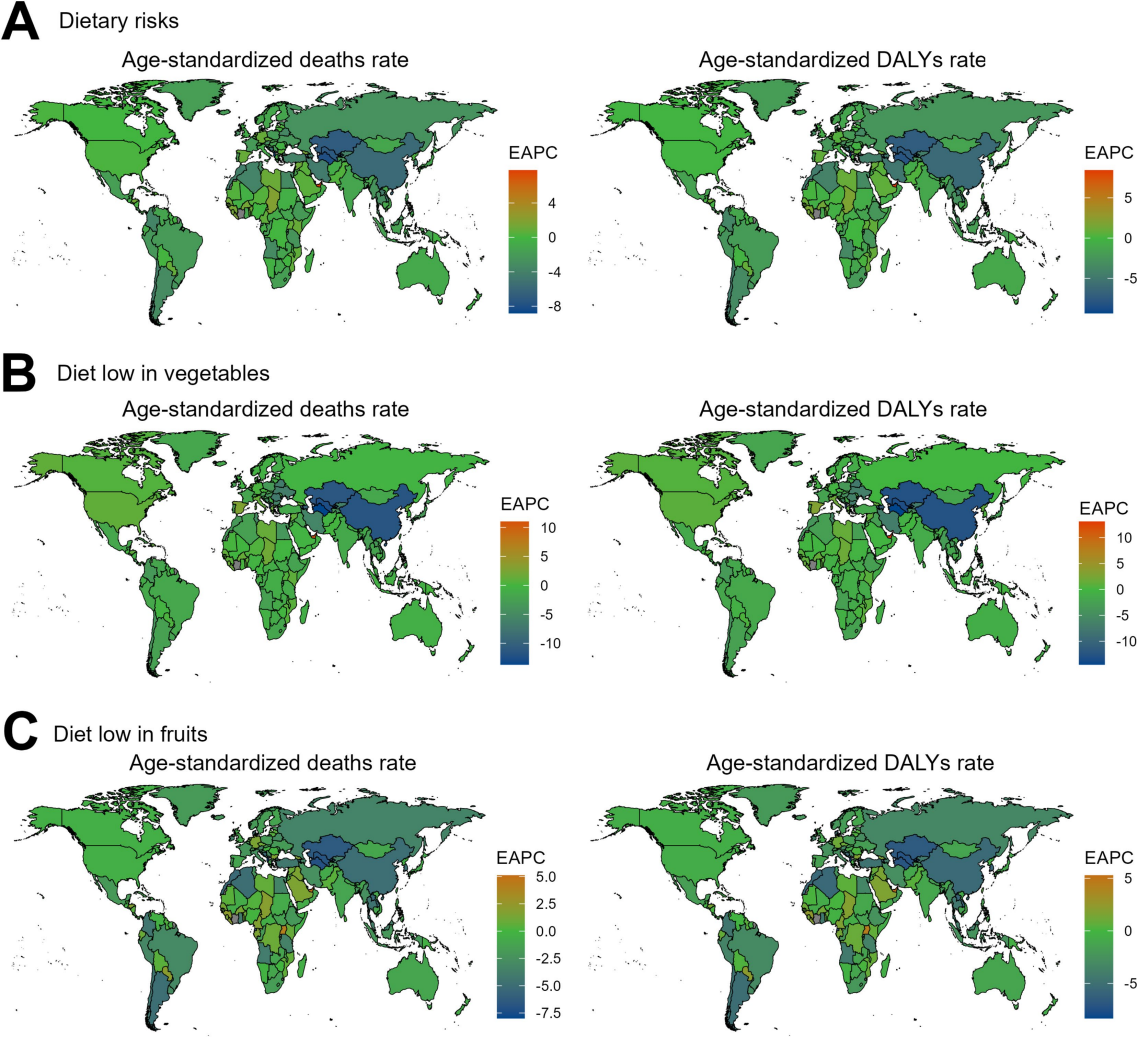
The EAPC value for the age-standardized deaths and DALYs rates of esophageal cancer attributable to diet low in vegetables and fruits from 1990 to 2019. EAPC, estimated annual percentage change; DALYs, disability-adjusted-life-years. **(A)** Dietary risks: Significant decreases in South Asia and Sub-Saharan Africa, while increases are seen in Eastern Europe and Central Asia. **(B)** Diet low in vegetables: Decreases in South Asia, with increases in Eastern Europe and Latin America. **(C)** Diet low in fruits: Decreases in East Asia and Southeast Asia, but increases in Eastern Europe and Latin America.

**Figure 4:**
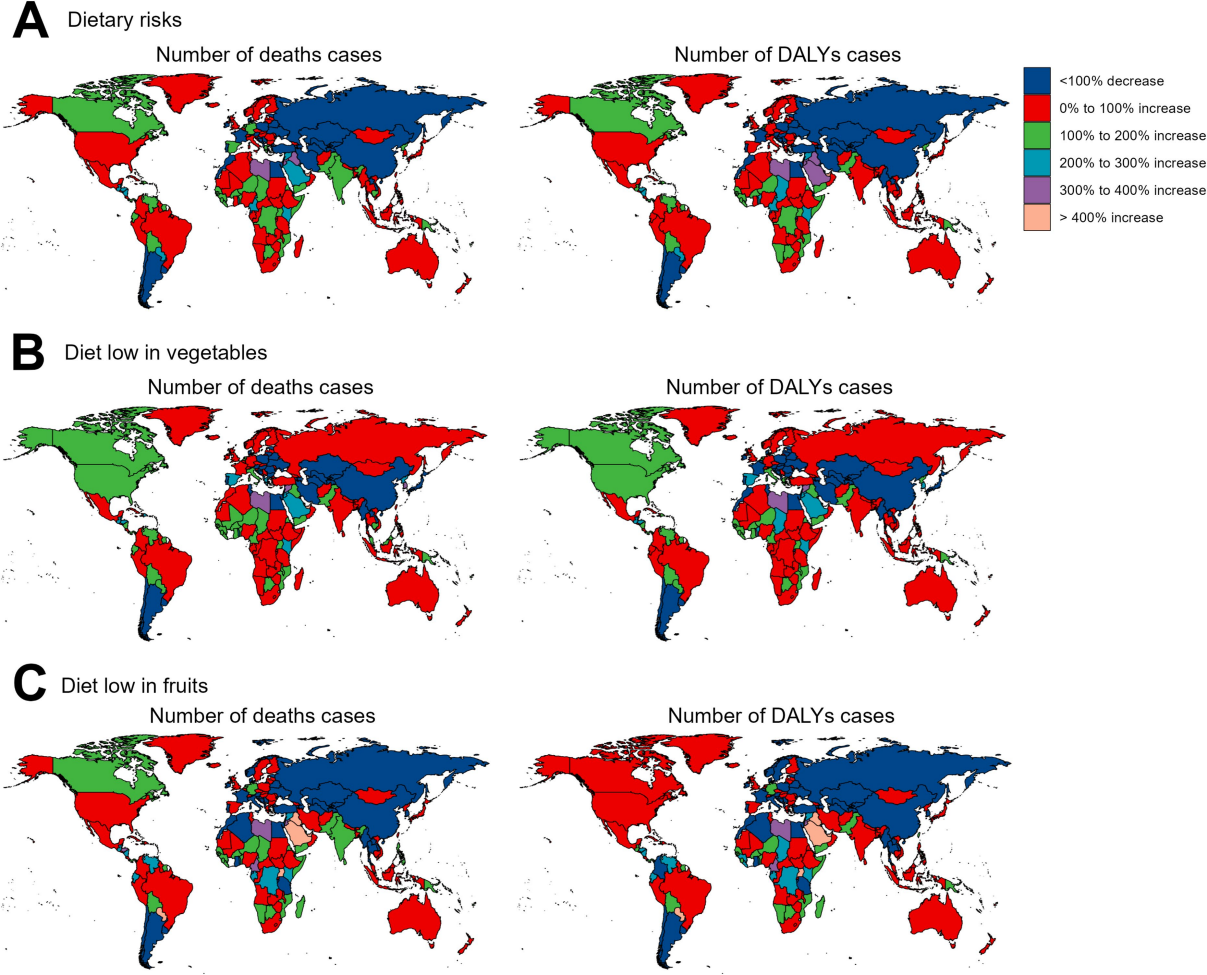
The relative change in the numbers of deaths and DALYs cases of esophageal cancer attributable to diet low in vegetables and fruits between 1990 and 2019. DALYs, disability-adjusted-life-years. **(A)** Dietary risks: Significant increases in deaths and DALYs are observed in most regions, especially in parts of Africa and Asia, while decreases are evident in some high-income regions. **(B)** Diet low in vegetables: Large increases in burden are noted in Eastern Europe and parts of Africa, with some reductions in specific high-income regions. **(C)** Diet low in fruits: Increases dominate in Africa, Asia, and Eastern Europe, while reductions are concentrated in a few high-income countries.

Regarding the 204 countries and territories examined, the temporal trend in the disease burden of EC attributable to diets low in vegetables and fruits also varied significantly from 1990 to 2019 (Figures 3, 4; Supplementary Tables S1–S6).

### 3.3 Decomposition analysis of age-standardized deaths and DALYs rates

**Figure 5:**
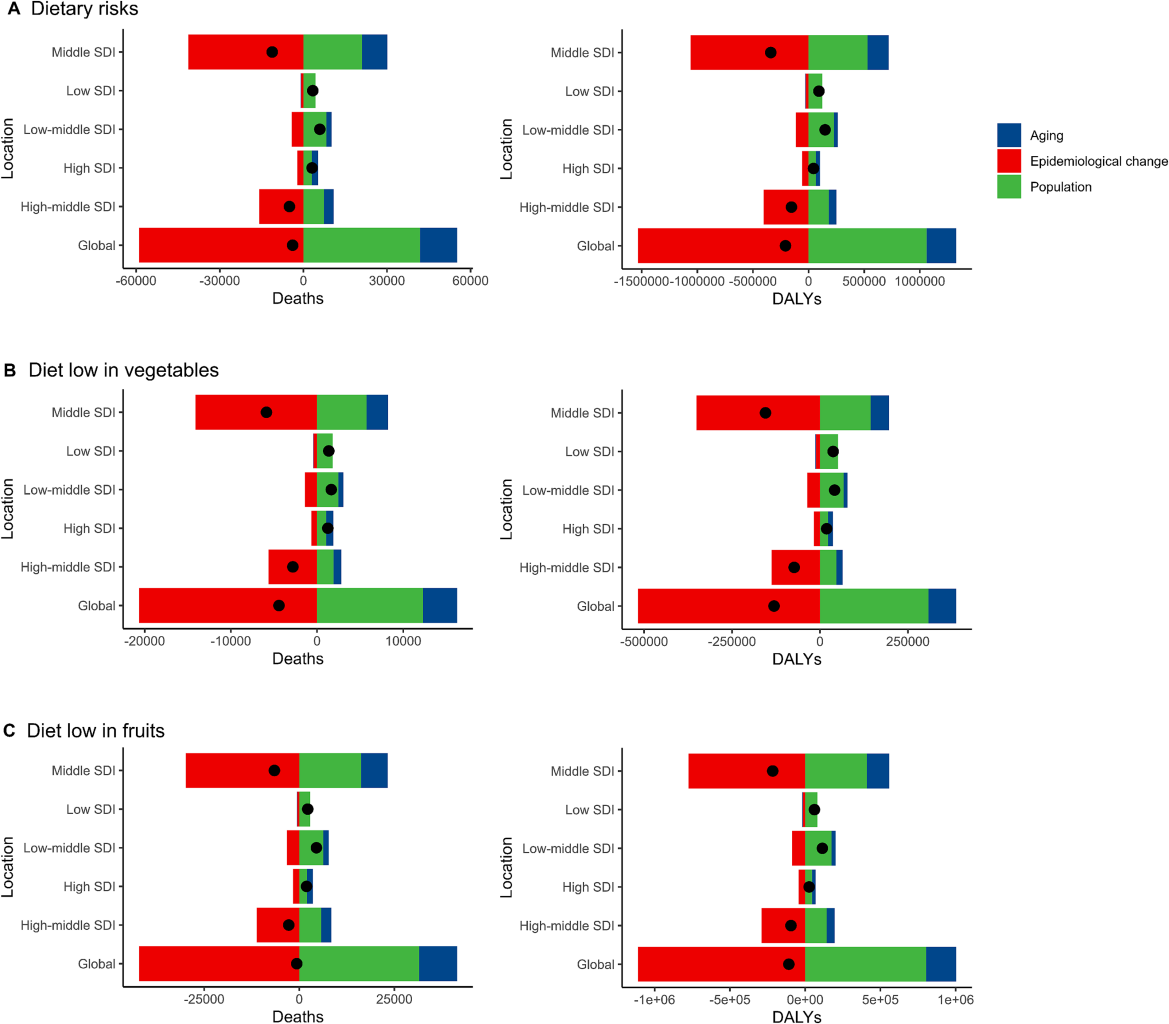
Alterations in the number of desths and DALYs cases of esophageal cancer attributable to diet low in vegetables and fruits based on the population-level determinants of population growth, aging, and epidemiological alteration between 1990 and 2019 at the global level and by SDI quintile. DALYs, Disability-Adjusted Life Years; SDI, Socio-demographic index. **(A)** Dietary risks. Global reductions due to epidemiological improvements were offset by increases from population growth and aging, especially in low and middle SDI regions. **(B)** Diet low in vegetables. Population growth was the main driver of increases in low and low-middle SDI regions, while high SDI regions experienced minimal change. **(C)** Diet low in fruits. Low SDI regions faced the greatest increases from population growth, while epidemiological improvements reduced the burden in high SDI regions.

Over the past 30 years, there has been a notable global decline in the number of deaths and DALYs cases of EC attributable to dietary risks. This reduction has been primarily driven by positive epidemiological change, leading to a significant decrease in deaths (1521.51%) and DALYs (738.93%) worldwide. However, these improvements have been partially offset by negative alterations related to population growth (−1078.54% in deaths, −511.29% in DALYs) and aging (−342.97% in deaths, −127.63% in DALYs) during the same period. The increase in absolute case numbers due to aging was particularly pronounced in high-SDI regions, while population growth contributed more significantly to the disease burden in low- and middle-SDI regions. Additionally, the extent of epidemiological improvements varied across regions, with greater progress observed in high-SDI regions compared to low-SDI regions. When analyzing the trends across different socio-demographic index (SDI) regions, it was observed that the reduction in deaths and DALYs cases was most prominent in the middle SDI regions, followed by the high-middle SDI regions. Conversely, the number of deaths and DALYs cases of EC increased in the high SDI, low SDI, and low-middle SDI regions, primarily attributed to population growth. Notably, the findings related to EC attributable to diets low in vegetables and fruits closely mirrored the overall trends observed for EC associated with dietary risks (Figure 5; Supplementary Tables S7, S8).

### 3.4 Frontier analysis of age-standardized deaths and DALYs rates

The unrealized health gains achieved by countries or regions across various levels of development between 1990 and 2019 are depicted in Supplementary Figures S11, S12. These figures illustrated the disease burden and the effective differences among countries or regions with differing sociodemographic development levels in 2019. As sociodemographic development progressed, the effective difference generally increased to a certain extent, suggesting that countries or regions with a higher SDI possess greater potential for improving their health burden (Supplementary Figures S11, S12).

## 4 Discussion

As far as we know, this was the first study to comprehensively assess and quantify the disease burden of EC attributable to diet low in vegetables and fruits globally. In 2019, this condition imposed a significant disease burden worldwide, with notable disparities observed across genders, age groups, SDI regions, GBD regions, and individual countries. Despite a global decline in age-standardized rates from 1990 to 2019, the number of deaths and DALYs has recently demonstrated an upward trend. Furthermore, our decomposition analysis revealed a positive impact of epidemiological changes on the reduction of disease burden. Finally, the frontier analysis indicated that countries or regions with a higher SDI posed greater potential for further reducing their health burden.

The results presented in this study highlighted the significant impact of dietary risks, including diet low in vegetables and fruits, on the global burden of EC. Notably, the deaths and DALYs of diets low in fruits were significantly higher than those of diets low in vegetables. This finding suggested that the lack of fruits in the diet might pose a greater risk for EC than the lack of vegetables. From a biological perspective, fruits are rich in antioxidants such as vitamin C, polyphenols, and flavonoids, which help neutralize reactive oxygen species (ROS) and reduce oxidative damage to cells, thereby providing significant protective effects against the development of EC. In contrast, the health benefits of vegetables are primarily derived from dietary fiber, which promotes gut health. This functional difference may partly explain the higher burden associated with inadequate fruit intake compared to insufficient vegetable intake. Additionally, cultural and dietary patterns exacerbate this disparity in different regions. For example, in low-SDI regions, fruit intake is limited due to high costs, seasonal availability, and inadequate storage conditions, whereas vegetables, being a staple part of daily diets, are relatively more accessible. In high-SDI regions, despite greater fruit availability, the increased consumption of processed fruit products (e.g., candied fruits and fruit juices) may diminish their health benefits. Based on these findings, this study proposes targeted public health strategies to address dietary risks in regions with different SDI levels. In low-SDI regions, governments should subsidize fruit and vegetable production to promote local cultivation and improve food accessibility. In high-SDI regions, efforts should focus on enhancing health education about balanced diets and regulating processed food consumption to reduce the negative health impacts of high-sugar fruit products (19, 20). However, it was important to recognize that both vegetables and fruits were essential components of a healthy diet, and their combined intake was likely to have a synergistic effect on reducing the risk of EC (21, 22). These results underscored the need for targeted interventions to address the dietary risks associated with EC. These interventions should focus on promoting the consumption of vegetables and fruits, which are rich in nutrients that may help protect against EC. Additionally, further research was needed to better understand the relationship between dietary factors and EC risk, as well as to identify effective strategies for reducing the global burden of this disease.

Between 1990 and 2019, EC deaths and DALYs of dietary risks exhibited complex trends. Initially, an increase was observed, followed by a decline, yet recently, numbers have resumed an upward trend. Notably, despite these fluctuations, age-standardized rates decreased, indicating a reduction in risk per capita. Interestingly, trends in EC of low fruit intake mirrored overall dietary risks, suggesting a significant role in fruit consumption. Conversely, trends for EC of low vegetable intake were more variable. This divergence highlighted the need for a nuanced understanding of dietary components contributing to EC risk, emphasizing the importance of continued vigilance and enhanced efforts to mitigate dietary impacts.

The findings presented in this study demonstrated a significant gender disparity in the burden of dietary risks-related EC, with males experiencing a notably higher burden compared to females. This observation was particularly noteworthy given the consistency of the gender disparity across both diets low in vegetables and fruits. The consistency suggested that dietary patterns might play a significant role in explaining these differences. Males might have less access to, or consume less, fruits and vegetables than females, which might contribute to their increased risk of EC (23, 24). Alternatively, there might be biological or hormonal differences between males and females that influence their susceptibility to the harmful effects of poor dietary habits (25, 26). Gender differences may also be influenced by cultural factors. For instance, in certain regions, men are more likely to engage in behaviors such as smoking and alcohol consumption, which may compound the effects of dietary risks and further exacerbate the disease burden. Public health strategies should take this into account by developing targeted interventions for male populations, combining health education on balanced diets with behavioral management programs. Future studies should aim to explore the potential reasons for this disparity in more detail.

The study revealed the differences in disease burden across age groups, necessitating deeper exploration. The initial rise in deaths and DALYs cases with age was likely due to the cumulative effects of long-term exposure to dietary risks (27). As individuals age, they might have had longer durations of exposure to diets low in vegetables and fruits, which were associated with an increased risk of EC. This cumulative effect might explain the gradual increase in cases observed in younger and middle-aged adults. The peak at a specific age group implied a critical period of vulnerability to EC, possibly due to aging effects on the esophageal mucosa, dietary habit changes, or comorbidities that enhance cancer risk (28–30). The subsequent decline in cases among the oldest might be attributed to prior mortality from EC or other health conditions, as well as diagnostic challenges due to comorbidities or frailty. These findings underscore the importance of early interventions. Dietary education and targeted interventions for adolescents and young adults can significantly reduce the risk of related diseases over the long term. Future research should further investigate the dietary exposure characteristics of different age groups and evaluate the potential of early dietary improvements in preventing EC.

The analysis of SDI regions reveals the complexity of the EC disease burden associated with dietary risks. Overall, age-standardized rates (ASR) for deaths and DALYs are significantly negatively correlated with SDI, with a higher burden observed in low-SDI regions. This disparity likely reflects challenges in accessing healthy foods and a lack of basic healthcare resources (31–33). However, the absolute burden peaks in middle-SDI regions, potentially due to high population density and increased exposure to risks during dietary transition phases. Residents in middle-SDI regions may be transitioning from traditional healthy diets to patterns characterized by high sugar and fat intake, which further exacerbate the risk of related diseases. Agricultural policies and the stability of food supply chains likely play critical roles in burden disparities across SDI regions. In low-SDI regions, weak agricultural infrastructure and the scarcity of fresh vegetables and fruits intensify dietary risks, while middle- and high-SDI regions benefit from well-developed agricultural and healthcare infrastructures, leading to comparatively lower burdens. EC related to low vegetable intake exhibits a “U-shaped” relationship, indicating higher relative risks at both ends of the SDI spectrum. This pattern may result from the interplay between healthcare infrastructure and dietary habits. In contrast, EC linked to low fruit intake is more concentrated and closely associated with high population density and dietary changes in middle-SDI regions (34, 35). From 1990 to 2019, the burden of EC related to dietary risks has risen significantly in both high-SDI and low-SDI regions. This trend reflects the complex interplay of dietary habits, food accessibility, and socioeconomic factors, highlighting that dietary risks remain an ongoing challenge across diverse socioeconomic contexts (36–38). These findings underscore the necessity of tailoring interventions based on SDI levels. In low-SDI regions, priority should be given to improving agricultural production and access to healthy foods. In high-SDI regions, health education and policy interventions should focus on curbing unhealthy dietary behaviors. Meanwhile, in middle-SDI regions, public health policies should address new risks arising from dietary transitions, providing targeted support to mitigate the burden. These region-specific strategies will contribute to effectively reducing the EC burden and promoting health equity. Our analysis further revealed disparities in disease burden across regions. In high-SDI regions, although aging is the primary driver of increased disease burden, these regions benefit from more robust health infrastructure, allowing for more effective implementation of screening and early treatment. In contrast, in low-SDI regions, population growth and insufficient infrastructure have led to a significant rise in disease burden. These regions urgently need to take measures to improve public health, particularly in the area of diet. Additionally, middle and high-SDI regions have made significant progress in epidemiological improvements, highlighting the crucial role of health policy interventions and resource allocation in reducing disease burden.

Across the 45 GBD regions and 204 countries studied, our analysis revealed substantial variation in the disease burden. This heterogeneity underscored the importance of regional and country-specific considerations when devising public health strategies. Hierarchical clustering analysis provided valuable insights into the patterns of variation in EC burden. These disparities could be attributed to various factors, including differences in dietary habits, cultural practices, access to healthy foods, and the implementation of preventive health measures (39, 40). The clustering analysis also revealed distinct patterns for the more specific dietary risks of low vegetable and fruit intake. This suggested that the impact of these dietary factors on EC burden may vary across regions. Understanding these regional patterns was crucial for developing targeted interventions that were responsive to the unique needs and challenges of each region. Turning our attention to the 204 countries and territories examined, we observed some countries experienced a marked increase in this burden, while others demonstrated a decrease. These temporal changes might reflect improvements or deteriorations in dietary habits, access to healthy foods, and the effectiveness of public health interventions over time (41, 42).

Over the past 30 years, there has been a significant decline in EC deaths and DALYs attributable to dietary risks globally, reflecting progress in dietary improvements, enhanced healthcare services, and widespread health education. However, these achievements still face certain challenges. Epidemiological changes have played a key role in reducing the burden of EC, reflecting the positive effects of recent improvements in dietary habits, healthcare services, and health education. Nonetheless, population growth and aging have exacerbated the health burden in low-SDI regions, partially offsetting these gains. Resource constraints in low and middle-SDI regions have placed greater pressure on these areas in reducing disease burden, while high-SDI regions, despite progress through advanced health systems, still require further policy interventions, especially in controlling unhealthy dietary behaviors and processed food consumption. Nevertheless, frontier analysis suggests that optimizing dietary interventions can significantly reduce the disease burden across SDI regions. For instance, in high-SDI countries, improvements in dietary policies and resource allocation could further narrow the gap between observed and potential health outcomes. Meanwhile, low and middle-SDI regions should prioritize basic public health interventions, such as nutritional supplementation programs and early disease diagnosis, to address the challenges posed by population growth and aging. Future interventions should integrate dietary education, behavioral management, and health screening, particularly by using predictive models to identify high-risk populations, providing scientific support for dietary improvements, and further enhancing cancer treatment outcomes (43, 44). This study has several limitations. First, the assessment of disease burden was primarily conducted at the national and regional levels, which may not fully capture the spatial heterogeneity within individual countries. Such spatial differences could manifest in disease risk exposure and the effectiveness of interventions, but this study did not delve into finer-scale analyses. Future research should leverage higher-resolution regional data to reveal the spatial distribution characteristics of disease burden. Second, the quality of data in the GBD database may affect the accuracy of the results. While the GBD database provides robust estimates through population surveys, health registries, and published literature, data limitations in low-income or data-scarce regions may increase uncertainty in burden estimates. There is a need to further improve data collection and validation processes, particularly in regions with limited data coverage (15, 45). Third, this study primarily attributed the EC burden to dietary risks, but other important behavioral and environmental confounding factors were not fully considered. For instance, smoking and alcohol consumption are significant risk factors for EC and may interact with dietary habits in complex ways. Additionally, socioeconomic factors (e.g., income level, healthcare accessibility) and cultural contexts may indirectly influence disease burden through various pathways. However, the independent attribution model employed by GBD does not fully integrate these interaction effects, potentially underestimating their combined impact. In the analysis of temporal trends, while linear regression models effectively quantify trends and EAPC offers simplicity and comparability, the assumption of linearity may limit the ability to capture nonlinear trends, particularly in regions with complex changes in disease burden. Furthermore, the sensitivity of hierarchical clustering analysis to outliers and data distribution could affect the stability and interpretability of the grouping results. Future research should explore more flexible time-series analysis methods, such as segmented regression or nonlinear regression, and attempt more robust clustering techniques, such as Gaussian mixture model-based clustering, to validate and supplement current findings. Lastly, this study provides a global analysis of dietary-related disease burden, but the generalizability of the results may be limited by regional differences in culture and dietary habits. For example, dietary risk exposure levels may vary significantly due to economic, cultural, or policy differences, potentially affecting the universality of the findings. Future studies should integrate region-specific health data and explore the complex interactions of behavioral, environmental, and socioeconomic factors to provide a deeper understanding of their impact on disease burden.

## 5 Conclusion

This study highlights significant disparities in the burden of EC attributable to dietary risks across different SDI regions and populations, underscoring the importance of implementing targeted dietary interventions. In high-SDI regions, priority should be given to promoting healthy eating habits through evidence-based dietary guidelines, subsidies for healthy foods, and education programs. In middle-SDI regions, community health support measures, such as improving fresh food supply chains and expanding health education on nutrition, should be strengthened. For low-SDI regions, efforts should focus on addressing nutritional deficiencies by implementing supplementation programs and improving access to healthy foods, thereby reducing dietary risk exposure. In regions with a high EC burden, integrated management of behaviors such as smoking and alcohol consumption should complement dietary interventions to minimize the additive effects of multiple risk factors. Public health strategies must account for regional cultural, economic, and social contexts to tailor interventions that are both feasible and effective. Future research should further validate the effectiveness of these strategies and explore the synergies between dietary interventions and other health behavior management approaches. These efforts will provide a scientific basis for globally controlling the EC burden effectively and equitably.

## Data Availability

The original contributions presented in the study are included in the article/Supplementary material, further inquiries can be directed to the corresponding author.
