# Supplementary material for: Global, regional, and national esophageal cancer deaths and DALYs attributable to diet low in vegetables and fruits, 1990–2019: analysis for the global burden of disease study
Source: Front Nutr. 2025 Jan 7;11:1478325. doi: 10.3389/fnut.2024.1478325 (PMC11747376; doi:10.3389/fnut.2024.1478325)
Supplement: Supplementary file 1 [file Table_1.docx]

**Supplementary Information**

**Global, regional, and national esophageal cancer deaths and DALYs attributable to diet low in vegetables and fruits, 1990-2019: analysis for the Global Burden of Disease Study.**

Bing Cui^1^, Aqin Chen^1^, Chengcheng Xu^2^, Chaoming Mao^2^, yuehua Chen^2^

^1^ Department of Blood Transfusion, Affiliated Hospital of Jiangsu University, Zhenjiang 212001, Jiangsu Province, China

^2^ Department of Nuclear Medicine, Affiliated Hospital of Jiangsu University, Zhenjiang 212001, Jiangsu Province, China;

**Table S1.** The number of deaths cases and the age-standardized deaths rate of esophageal cancer attributable to dietary risks in 1990 and 2019, and its trends from 1990 to 2019 globally.

**Table S2.** The number of DALYs cases and the age-standardized DALYs rate of esophageal cancer attributable to dietary risks in 1990 and 2019, and its trends from 1990 to 2019 globally. Abbreviations: DALYs, disability-adjusted life years.

**Table S3.** The number of deaths cases and the age-standardized deaths rate of esophageal cancer attributable to diet low in vegetables in 1990 and 2019, and its trends from 1990 to 2019 globally.

**Table S4.** The number of DALYs cases and the age-standardized DALYs rate of esophageal cancer attributable to diet low in vegetables in 1990 and 2019, and its trends from 1990 to 2019 globally. Abbreviations: DALYs, disability-adjusted life years.

**Table S5.** The number of deaths cases and the age-standardized deaths rate of esophageal cancer attributable to diet low in fruits in 1990 and 2019, and its trends from 1990 to 2019 globally.

**Table S6.** The number of DALYs cases and the age-standardized DALYs rate of esophageal cancer attributable to diet low in fruits in 1990 and 2019, and its trends from 1990 to 2019 globally. Abbreviations: DALYs, disability-adjusted life years.

**Table S7.** Changes in deaths numbers of esophageal cancer attributable to diet low in vegetables and fruits based on the population-level determinants and causes between 1990 and 2019.

**Table S8.** Changes in DALYs numbers of esophageal cancer attributable to diet low in vegetables and fruits based on the population-level determinants and causes between 1990 and 2019. Abbreviations: DALYs, disability-adjusted life years.

**Figure S1.** Numbers and age-standardized rates of esophageal cancer attributable to diet low in vegetables and fruits deaths and DALYs for different sex in 2019. Abbreviations: DALYs, disability-adjusted life years.

**Figure S2.** Numbers and age-standardized rates of esophageal cancer attributable to diet low in vegetables and fruits deaths and DALYs for different age groups in 2019. Abbreviations: DALYs, disability-adjusted life years.

**Figure S3.** Numbers and age-standardized rates of esophageal cancer attributable to diet low in vegetables and fruits deaths and DALYs for different SDI regions in 2019. Abbreviations: DALYs, disability-adjusted life years.

**Figure S4.** Numbers and age-standardized rates of esophageal cancer attributable to diet low in vegetables and fruits deaths and DALYs for different GBD regions in 2019. Abbreviations: DALYs, disability-adjusted life years.

**Figure S5.** Numbers and age-standardized rates of esophageal cancer attributable to dietary risks deaths and DALYs across countries and territories in 2019. Abbreviations: DALYs, disability-adjusted life years.

**Figure S6.** Numbers and age-standardized rates of esophageal cancer attributable to diet low in vegetables deaths and DALYs across countries and territories in 2019. Abbreviations: DALYs, disability-adjusted life years.

**Figure S7.** Numbers and age-standardized rates of esophageal cancer attributable to diet low in fruits deaths and DALYs across countries and territories in 2019. Abbreviations: DALYs, disability-adjusted life years.

**Figure S8.** Trends in the numbers and age-standardized rates of esophageal cancer attributable to diet low in vegetables and fruits deaths and DALYs globally by sex from 1990 to 2019. Abbreviations: DALYs, disability-adjusted-life-years.

**Figure S9.** Trends in the numbers and age-standardized rates of esophageal cancer attributable to diet low in vegetables and fruits deaths and DALYs globally by age groups from 1990 to 2019. Abbreviations: DALYs, disability-adjusted-life-years.

**Figure S10.** Trends in the numbers and age-standardized rates of esophageal cancer attributable to diet low in vegetables and fruits deaths and DALYs globally by SDI regions from 1990 to 2019. Abbreviations: DALYs, disability-adjusted-life-years.

**Figure S11.** Frontier analysis based on SDI and age-standardized diet low in vegetables and fruits-related esophageal cancer deaths and DALYs rate in 2019. The frontier is delineated in solid black color; countries and territories are represented as dots. Abbreviations: SDI, Socio-demographic index; DALYs, Disability-Adjusted Life Years.

**Figure S12.** Frontier analysis based on SDI and age-standardized diet low in vegetables and fruits-related esophageal cancer deaths and DALYs rate in 2019. The frontier is delineated in solid black color; countries and territories are represented as dots. The top 15 countries with the largest effective difference (largest smoking-related bladder cancer DALYs gap from the frontier) are labeled in black; examples of frontier countries with low SDI (<0.5) and low effective difference are labeled in blue, and examples of countries and territories with high SDI (>0.85) and relatively high effective difference for their level of development are labeled in red. Red dots indicate an increase in age-standardized smoking-related bladder cancer DALYs rate from 1990 to 2019; blue dots indicate a decrease in age-standardized smoking-related bladder cancer DALYs rate between 1990 and 2019. Abbreviations: SDI, Socio-demographic index; DALYs, Disability-Adjusted Life Years.

**Table S1.** The number of deaths cases and the age-standardized deaths rate of esophageal cancer attributable to dietary risks in 1990 and 2019, and its trends from 1990 to 2019 globally.

|  | Number of deaths  cases (95% UI) in 1990 | The age-standardized deaths rate/100000 (95% UI) in 1990 | Number of deaths  cases (95% UI) in 2019 | The age-standardized deaths rate/100000 (95% UI) in 2019 | EAPC (95% CI) |
| --- | --- | --- | --- | --- | --- |
| Global | 69798 (32099-113506) | 1.78 (0.82-2.9) | 65919 (26635-121827) | 0.81 (0.33-1.5) | -3.08 (-3.3--2.86) |
| **Sex** |  |  |  |  |  |
| Female | 23963 (11363-38633) | 1.14 (0.54-1.84) | 20187 (9054-34986) | 0.46 (0.21-0.8) | -3.57 (-3.82--3.31) |
| Male | 45835 (20663-74447) | 2.54 (1.14-4.13) | 45732 (17539-89677) | 1.21 (0.46-2.38) | -2.85 (-3.06--2.64) |
| **Age** |  |  |  |  |  |
| <24 years | 0 (0-0) | 0 (0-0) | 0 (0-0) | 0 (0-0) | 0 (0-0) |
| 25-29 years | 183 (100-267) | 0.04 (0.02-0.06) | 162 (86-245) | 0.03 (0.01-0.04) | -2.12 (-2.42--1.83) |
| 30-34 years | 357 (183-527) | 0.09 (0.05-0.14) | 351 (176-542) | 0.06 (0.03-0.09) | -2.15 (-2.48--1.83) |
| 35-39 years | 906 (456-1346) | 0.26 (0.13-0.38) | 750 (368-1182) | 0.14 (0.07-0.22) | -2.43 (-2.73--2.13) |
| 40-44 years | 2145 (1073-3334) | 0.75 (0.37-1.16) | 1656 (797-2687) | 0.34 (0.16-0.54) | -3.36 (-3.61--3.12) |
| 45-49 years | 3403 (1652-5317) | 1.46 (0.71-2.29) | 3183 (1426-5439) | 0.67 (0.3-1.15) | -3.22 (-3.52--2.93) |
| 50-54 years | 6050 (2921-9483) | 2.85 (1.37-4.46) | 5443 (2391-9653) | 1.25 (0.55-2.21) | -3.29 (-3.61--2.96) |
| 55-59 years | 8816 (3985-14301) | 4.75 (2.15-7.71) | 7310 (3102-13085) | 1.97 (0.84-3.53) | -3.26 (-3.44--3.08) |
| 60-64 years | 10708 (4760-17616) | 6.67 (2.96-10.97) | 8304 (3315-16065) | 2.66 (1.06-5.14) | -3.42 (-3.55--3.28) |
| 65-69 years | 11350 (4998-18827) | 9.19 (4.05-15.25) | 9435 (3597-18564) | 3.65 (1.39-7.18) | -3.52 (-3.7--3.34) |
| 70-74 years | 10560 (4539-17269) | 12.49 (5.37-20.43) | 9334 (3503-18594) | 4.99 (1.87-9.94) | -3.55 (-3.8--3.31) |
| 75-79 years | 8035 (3718-13097) | 13.11 (6.06-21.36) | 7704 (2925-15062) | 6.06 (2.3-11.85) | -3.26 (-3.55--2.97) |
| 80-84 years | 4764 (2267-7686) | 13.53 (6.44-21.82) | 6422 (2349-12603) | 7.61 (2.78-14.93) | -2.3 (-2.64--1.96) |
| 85-89 years | 1938 (846-3204) | 12.86 (5.61-21.26) | 4105 (1402-8023) | 9.44 (3.22-18.45) | -1.24 (-1.56--0.92) |
| 90-94 years | 481 (202-787) | 10.93 (4.57-17.86) | 1381 (522-2540) | 8.19 (3.1-15.07) | -1.11 (-1.3--0.91) |
| 95+ years | 101 (42-171) | 9.79 (4.12-16.63) | 378 (132-709) | 7.93 (2.76-14.86) | -0.61 (-0.69--0.54) |
| **SDI regions** |  |  |  |  |  |
| High SDI | 7400 (2406-14118) | 0.71 (0.23-1.36) | 10491 (3138-20798) | 0.55 (0.17-1.09) | -0.9 (-0.96--0.85) |
| High-middle SDI | 17173 (7400-28731) | 1.62 (0.7-2.71) | 12150 (3691-27665) | 0.59 (0.18-1.35) | -3.92 (-4.19--3.65) |
| Middle SDI | 32511 (14282-53089) | 3.32 (1.47-5.4) | 21312 (7501-44618) | 0.9 (0.32-1.88) | -4.92 (-5.31--4.53) |
| Low-middle SDI | 8991 (4985-13181) | 1.55 (0.85-2.28) | 14874 (7911-23037) | 1.11 (0.59-1.71) | -1.38 (-1.48--1.27) |
| Low SDI | 3710 (1917-5531) | 1.61 (0.83-2.41) | 7070 (3593-10780) | 1.4 (0.7-2.13) | -0.61 (-0.7--0.52) |
| **GBD regions** |  |  |  |  |  |
| Africa | 4427 (2207-6806) | 1.6 (0.79-2.45) | 8053 (3911-12311) | 1.33 (0.65-2.05) | -0.9 (-1.11--0.69) |
| African Region | 4147 (2091-6319) | 1.92 (0.97-2.91) | 7548 (3690-11581) | 1.61 (0.8-2.47) | -0.88 (-1.11--0.65) |
| America | 3701 (1015-7213) | 0.61 (0.17-1.19) | 5921 (1520-11518) | 0.46 (0.12-0.9) | -0.99 (-1.05--0.93) |
| Andean Latin America | 71 (26-122) | 0.37 (0.14-0.63) | 132 (37-255) | 0.24 (0.07-0.47) | -1.46 (-1.54--1.38) |
| Asia | 54784 (25671-87241) | 2.83 (1.32-4.52) | 44613 (17496-86671) | 0.95 (0.37-1.85) | -4.14 (-4.43--3.85) |
| Australasia | 170 (51-325) | 0.72 (0.22-1.39) | 296 (71-604) | 0.58 (0.14-1.19) | -0.86 (-0.92--0.8) |
| Caribbean | 161 (50-298) | 0.64 (0.2-1.17) | 223 (61-439) | 0.43 (0.12-0.85) | -1.52 (-1.78--1.26) |
| Central Asia | 1358 (580-2218) | 3 (1.29-4.87) | 570 (181-1244) | 0.85 (0.28-1.82) | -5.22 (-5.52--4.91) |
| Central Europe | 655 (207-1279) | 0.45 (0.15-0.88) | 697 (187-1524) | 0.34 (0.09-0.74) | -1.28 (-1.46--1.1) |
| Central Latin America | 302 (84-566) | 0.39 (0.11-0.73) | 551 (142-1046) | 0.24 (0.06-0.45) | -1.92 (-2.06--1.79) |
| Central Sub-Saharan Africa | 548 (197-992) | 2.51 (0.9-4.57) | 1109 (463-1877) | 2.2 (0.9-3.71) | -0.74 (-0.97--0.51) |
| Commonwealth High Income | 1715 (697-2965) | 1.13 (0.46-1.95) | 2406 (818-4376) | 0.91 (0.31-1.66) | -0.95 (-1.04--0.87) |
| Commonwealth Low Income | 2174 (1175-3191) | 2.55 (1.38-3.74) | 3800 (1717-6188) | 1.77 (0.81-2.89) | -1.29 (-1.37--1.2) |
| Commonwealth Middle Income | 9014 (5280-12429) | 1.53 (0.89-2.12) | 17896 (9890-26059) | 1.23 (0.68-1.79) | -1.08 (-1.29--0.88) |
| East Asia | 40820 (17961-67136) | 4.91 (2.17-8.1) | 21132 (4611-53435) | 1.06 (0.24-2.69) | -5.67 (-6.14--5.19) |
| East Asia & Pacific - WB | 44398 (19778-73213) | 3.4 (1.52-5.63) | 26118 (6642-62350) | 0.85 (0.22-2.03) | -5.16 (-5.56--4.77) |
| Eastern Europe | 2458 (979-4138) | 0.87 (0.35-1.46) | 1737 (589-3240) | 0.51 (0.17-0.95) | -2.5 (-2.77--2.23) |
| Eastern Mediterranean Region | 1708 (804-2744) | 0.97 (0.45-1.55) | 3458 (1664-5583) | 0.81 (0.39-1.33) | -0.7 (-0.81--0.58) |
| Eastern Sub-Saharan Africa | 2123 (1077-3225) | 2.9 (1.48-4.4) | 3751 (1764-5919) | 2.37 (1.12-3.75) | -0.84 (-0.91--0.76) |
| Europe | 6824 (2481-12464) | 0.66 (0.24-1.21) | 7283 (2298-13833) | 0.47 (0.15-0.9) | -1.49 (-1.6--1.38) |
| Europe & Central Asia - WB | 8008 (3030-14317) | 0.76 (0.29-1.35) | 7637 (2399-14565) | 0.48 (0.15-0.93) | -1.92 (-2.05--1.8) |
| European Region | 8012 (3031-14333) | 0.75 (0.29-1.35) | 7645 (2400-14589) | 0.48 (0.15-0.92) | -1.93 (-2.06--1.81) |
| High-income Asia Pacific | 1596 (534-3038) | 0.8 (0.27-1.52) | 2057 (635-4265) | 0.45 (0.14-0.93) | -1.76 (-1.93--1.59) |
| High-income North America | 1692 (405-3556) | 0.49 (0.12-1.03) | 3076 (779-6291) | 0.49 (0.12-1) | 0.16 (0.1-0.21) |
| Latin America & Caribbean - WB | 2057 (612-3750) | 0.81 (0.24-1.47) | 2873 (697-5363) | 0.43 (0.11-0.81) | -2.32 (-2.44--2.19) |
| Middle East & North Africa - WB | 291 (80-571) | 0.25 (0.07-0.49) | 495 (152-1032) | 0.16 (0.05-0.34) | -1.81 (-2.07--1.55) |
| North Africa and Middle East | 518 (161-1000) | 0.31 (0.1-0.59) | 842 (272-1697) | 0.2 (0.06-0.41) | -1.9 (-2.07--1.74) |
| North America | 1692 (405-3555) | 0.49 (0.12-1.03) | 3076 (779-6291) | 0.49 (0.12-1) | 0.16 (0.1-0.21) |
| Oceania | 15 (7-27) | 0.57 (0.25-0.97) | 34 (14-58) | 0.52 (0.22-0.9) | -0.24 (-0.28--0.21) |
| Region of the Americas | 3701 (1015-7213) | 0.61 (0.17-1.19) | 5921 (1520-11518) | 0.46 (0.12-0.9) | -0.99 (-1.05--0.93) |
| South-East Asia Region | 9217 (5342-12930) | 1.37 (0.79-1.92) | 17286 (9456-25832) | 1.03 (0.56-1.53) | -1.23 (-1.35--1.11) |
| South Asia | 8724 (5107-11949) | 1.61 (0.94-2.22) | 17252 (9519-25121) | 1.25 (0.69-1.82) | -1.1 (-1.23--0.97) |
| South Asia - WB | 9007 (5236-12404) | 1.61 (0.94-2.22) | 17758 (9784-25762) | 1.25 (0.69-1.82) | -1.08 (-1.21--0.94) |
| Southeast Asia | 1854 (921-2828) | 0.74 (0.37-1.14) | 2752 (1056-4853) | 0.47 (0.18-0.82) | -1.71 (-1.76--1.66) |
| Southern Latin America | 548 (138-1067) | 1.23 (0.31-2.38) | 436 (87-881) | 0.52 (0.1-1.04) | -3.39 (-3.57--3.21) |
| Southern Sub-Saharan Africa | 1124 (592-1648) | 4.13 (2.19-6.04) | 1799 (961-2593) | 3.33 (1.78-4.77) | -1.32 (-1.87--0.77) |
| Sub-Saharan Africa - WB | 4331 (2184-6617) | 2 (1.01-3.06) | 7939 (3874-12149) | 1.73 (0.85-2.64) | -0.77 (-0.99--0.55) |
| Tropical Latin America | 980 (313-1762) | 1.1 (0.35-1.97) | 1539 (360-2847) | 0.63 (0.15-1.17) | -2 (-2.09--1.9) |
| Western Europe | 3623 (1195-6800) | 0.64 (0.21-1.2) | 4781 (1393-9019) | 0.53 (0.15-1) | -0.8 (-0.88--0.73) |
| Western Pacific Region | 42877 (18969-70704) | 3.78 (1.68-6.24) | 23926 (5755-58882) | 0.89 (0.22-2.18) | -5.37 (-5.79--4.95) |
| Western Sub-Saharan Africa | 458 (231-707) | 0.54 (0.28-0.83) | 1156 (565-1816) | 0.65 (0.32-1.02) | 0.92 (0.81-1.04) |
| World Bank High Income | 8010 (2570-15351) | 0.63 (0.2-1.21) | 11282 (3311-22227) | 0.5 (0.14-0.98) | -0.85 (-0.91--0.78) |
| World Bank Low Income | 3100 (1521-4879) | 2.13 (1.04-3.32) | 5691 (2629-8996) | 1.87 (0.86-2.97) | -0.57 (-0.66--0.48) |
| World Bank Lower Middle Income | 12187 (6818-17322) | 1.2 (0.67-1.71) | 22225 (11963-32534) | 0.96 (0.52-1.41) | -1.02 (-1.12--0.91) |
| World Bank Upper Middle Income | 46487 (20741-76865) | 3.17 (1.41-5.22) | 26701 (7326-62680) | 0.81 (0.22-1.9) | -5.13 (-5.54--4.73) |
| **Countries** |  |  |  |  |  |
| Afghanistan | 143 (43-256) | 2.05 (0.62-3.61) | 221 (63-398) | 1.83 (0.54-3.19) | -0.6 (-0.87--0.33) |
| Albania | 6 (1-14) | 0.32 (0.07-0.68) | 1 (0-4) | 0.03 (0.01-0.11) | -8.81 (-9.47--8.15) |
| Algeria | 29 (9-54) | 0.27 (0.09-0.51) | 32 (4-72) | 0.11 (0.01-0.24) | -3.71 (-3.97--3.45) |
| American Samoa | 0 (0-0) | 0.23 (0.08-0.43) | 0 (0-0) | 0.27 (0.09-0.49) | 0.58 (0.41-0.75) |
| Andorra | 0 (0-0) | 0.38 (0.06-0.88) | 0 (0-1) | 0.31 (0.05-0.72) | -1.02 (-1.17--0.87) |
| Angola | 129 (52-215) | 3.35 (1.4-5.59) | 170 (55-330) | 1.65 (0.54-3.07) | -3.21 (-3.52--2.9) |
| Antigua and Barbuda | 0 (0-0) | 0.42 (0.08-0.83) | 0 (0-1) | 0.4 (0.08-0.8) | -0.06 (-0.28-0.17) |
| Argentina | 341 (70-695) | 1.08 (0.22-2.2) | 261 (35-523) | 0.48 (0.06-0.96) | -3.2 (-3.41--3) |
| Armenia | 6 (1-16) | 0.22 (0.03-0.6) | 2 (0-8) | 0.06 (0.01-0.2) | -6.01 (-6.46--5.57) |
| Australia | 148 (47-276) | 0.76 (0.24-1.41) | 259 (65-526) | 0.61 (0.15-1.23) | -0.91 (-0.97--0.85) |
| Austria | 46 (8-96) | 0.41 (0.07-0.85) | 37 (4-83) | 0.22 (0.02-0.49) | -2.67 (-2.9--2.43) |
| Azerbaijan | 56 (8-134) | 1.15 (0.17-2.71) | 40 (4-143) | 0.49 (0.05-1.71) | -4.31 (-5.02--3.59) |
| Bahamas | 1 (0-3) | 0.82 (0.17-1.8) | 3 (1-6) | 0.7 (0.18-1.44) | -0.76 (-0.86--0.66) |
| Bahrain | 0 (0-1) | 0.25 (0.03-0.64) | 1 (0-2) | 0.08 (0.01-0.22) | -4.56 (-4.86--4.26) |
| Bangladesh | 1021 (586-1461) | 2.22 (1.29-3.18) | 1596 (804-2603) | 1.25 (0.63-2.04) | -1.95 (-2.09--1.81) |
| Barbados | 4 (2-7) | 1.52 (0.74-2.32) | 5 (2-9) | 1.11 (0.46-1.88) | -1.38 (-1.6--1.15) |
| Belarus | 84 (32-141) | 0.64 (0.25-1.08) | 54 (11-137) | 0.34 (0.07-0.86) | -3.46 (-3.9--3.01) |
| Belgium | 95 (26-187) | 0.63 (0.18-1.25) | 103 (16-239) | 0.45 (0.07-1.06) | -1.43 (-1.58--1.28) |
| Belize | 0 (0-0) | 0.21 (0.04-0.4) | 1 (0-1) | 0.24 (0.05-0.46) | 0.42 (0.13-0.71) |
| Benin | 21 (11-32) | 1.1 (0.57-1.63) | 62 (26-103) | 1.33 (0.57-2.22) | 1.04 (0.85-1.23) |
| Bermuda | 0 (0-1) | 0.62 (0.08-1.42) | 1 (0-2) | 0.69 (0.22-1.32) | 0.74 (0.58-0.89) |
| Bhutan | 4 (2-6) | 1.68 (0.82-2.57) | 6 (3-11) | 1.18 (0.5-2.05) | -1.21 (-1.34--1.08) |
| Bolivia (Plurinational State of) | 13 (3-27) | 0.45 (0.11-0.91) | 36 (10-70) | 0.44 (0.12-0.85) | 0.05 (-0.02-0.13) |
| Bosnia and Herzegovina | 22 (9-37) | 0.55 (0.23-0.92) | 10 (1-28) | 0.17 (0.02-0.48) | -5.16 (-5.56--4.75) |
| Botswana | 27 (13-43) | 4.83 (2.38-7.53) | 57 (29-89) | 4.25 (2.19-6.47) | -1.04 (-1.47--0.61) |
| Brazil | 971 (311-1745) | 1.11 (0.36-2) | 1510 (356-2802) | 0.64 (0.15-1.18) | -2.04 (-2.14--1.94) |
| Brunei Darussalam | 1 (0-2) | 1.04 (0.38-1.86) | 2 (1-3) | 0.69 (0.3-1.18) | -1.49 (-1.7--1.27) |
| Bulgaria | 27 (3-72) | 0.23 (0.02-0.59) | 32 (7-74) | 0.24 (0.06-0.56) | 0.55 (0.36-0.75) |
| Burkina Faso | 59 (34-84) | 1.39 (0.8-1.96) | 161 (86-237) | 1.85 (0.97-2.72) | 1.35 (1.17-1.53) |
| Burundi | 61 (16-123) | 2.62 (0.71-5.32) | 73 (17-151) | 1.63 (0.39-3.36) | -2.16 (-2.35--1.98) |
| Cabo Verde | 6 (3-10) | 2.8 (1.43-4.17) | 14 (6-24) | 3.33 (1.37-5.66) | -0.54 (-1.01--0.07) |
| Cambodia | 52 (27-79) | 1.17 (0.62-1.79) | 103 (53-158) | 0.91 (0.46-1.37) | -0.99 (-1.1--0.87) |
| Cameroon | 25 (5-49) | 0.58 (0.13-1.15) | 81 (14-183) | 0.72 (0.14-1.6) | 0.69 (0.45-0.94) |
| Canada | 136 (21-300) | 0.42 (0.07-0.93) | 303 (51-663) | 0.44 (0.07-0.96) | -0.02 (-0.15-0.11) |
| Central African Republic | 42 (16-75) | 3.58 (1.4-6.29) | 64 (27-110) | 2.96 (1.26-5.04) | -0.72 (-0.78--0.66) |
| Chad | 31 (18-46) | 1.13 (0.64-1.65) | 92 (47-141) | 1.71 (0.88-2.65) | 1.88 (1.67-2.09) |
| Chile | 136 (46-249) | 1.49 (0.5-2.73) | 125 (26-268) | 0.52 (0.11-1.11) | -4.18 (-4.4--3.96) |
| China | 40514 (17911-66618) | 5.07 (2.23-8.32) | 20509 (4338-52321) | 1.07 (0.24-2.73) | -5.74 (-6.22--5.26) |
| Colombia | 106 (25-208) | 0.66 (0.16-1.31) | 137 (29-281) | 0.26 (0.05-0.53) | -3.69 (-3.88--3.49) |
| Comoros | 5 (1-10) | 2.33 (0.54-4.77) | 10 (3-19) | 2.07 (0.62-3.93) | -0.62 (-0.78--0.47) |
| Congo | 39 (15-70) | 3.74 (1.46-6.58) | 61 (23-108) | 2.46 (0.94-4.33) | -1.69 (-1.87--1.5) |
| Cook Islands | 0 (0-0) | 0.61 (0.2-1.14) | 0 (0-0) | 0.4 (0.1-0.78) | -1.66 (-1.77--1.55) |
| Costa Rica | 7 (2-13) | 0.42 (0.12-0.78) | 12 (3-23) | 0.23 (0.05-0.46) | -2.29 (-2.55--2.03) |
| Croatia | 33 (6-72) | 0.51 (0.09-1.1) | 25 (4-56) | 0.29 (0.04-0.67) | -2.28 (-2.44--2.11) |
| Cuba | 54 (11-110) | 0.53 (0.11-1.07) | 82 (11-190) | 0.43 (0.06-1) | -0.87 (-1.17--0.57) |
| Cyprus | 1 (0-2) | 0.13 (0.01-0.27) | 3 (0-6) | 0.15 (0.02-0.31) | 0.83 (0.59-1.08) |
| Czechia | 62 (16-123) | 0.46 (0.12-0.91) | 106 (32-211) | 0.53 (0.16-1.04) | 0.74 (0.55-0.92) |
| Côte d'Ivoire | 26 (7-52) | 0.67 (0.18-1.37) | 95 (27-191) | 0.94 (0.28-1.86) | 1.56 (1.37-1.76) |
| Democratic People's Republic of Korea | 200 (33-493) | 1.28 (0.21-3.12) | 440 (102-918) | 1.37 (0.32-2.86) | 0.18 (-0.03-0.38) |
| Democratic Republic of the Congo | 324 (98-641) | 2.17 (0.65-4.24) | 795 (343-1333) | 2.34 (1.01-3.98) | 0.04 (-0.24-0.33) |
| Denmark | 73 (24-132) | 0.92 (0.31-1.67) | 47 (6-104) | 0.4 (0.05-0.9) | -3.25 (-3.67--2.82) |
| Djibouti | 7 (3-11) | 4.78 (2.43-7.44) | 23 (11-40) | 3.91 (1.99-6.64) | -0.95 (-1.1--0.8) |
| Dominica | 0 (0-1) | 0.4 (0.04-0.8) | 0 (0-1) | 0.38 (0.03-0.8) | -0.01 (-0.25-0.23) |
| Dominican Republic | 7 (2-14) | 0.2 (0.04-0.39) | 21 (3-44) | 0.23 (0.03-0.48) | 0.82 (0.68-0.97) |
| Ecuador | 13 (3-23) | 0.26 (0.06-0.48) | 28 (7-54) | 0.2 (0.05-0.38) | -0.8 (-0.99--0.61) |
| Egypt | 25 (3-78) | 0.09 (0.01-0.27) | 22 (4-90) | 0.04 (0.01-0.14) | -3.33 (-3.71--2.95) |
| El Salvador | 9 (3-16) | 0.31 (0.12-0.55) | 18 (5-34) | 0.29 (0.08-0.56) | -0.79 (-1.15--0.43) |
| Equatorial Guinea | 6 (2-11) | 3.09 (1.19-5.32) | 6 (2-13) | 1.36 (0.41-2.94) | -3.63 (-4.17--3.09) |
| Eritrea | 43 (19-74) | 4.2 (1.85-7.12) | 92 (41-160) | 3.5 (1.54-6.11) | -0.8 (-0.94--0.65) |
| Estonia | 14 (6-24) | 0.7 (0.3-1.16) | 11 (3-22) | 0.45 (0.12-0.92) | -1.78 (-2.05--1.51) |
| Eswatini | 13 (6-21) | 4.53 (2.04-7.27) | 22 (9-39) | 3.9 (1.64-6.78) | -0.78 (-1.43--0.12) |
| Ethiopia | 338 (186-556) | 1.69 (0.93-2.7) | 396 (220-613) | 1.02 (0.57-1.58) | -2.02 (-2.14--1.9) |
| Fiji | 3 (1-4) | 0.82 (0.44-1.22) | 5 (2-9) | 0.83 (0.39-1.33) | 0.41 (0.09-0.73) |
| Finland | 42 (15-75) | 0.59 (0.21-1.04) | 52 (16-100) | 0.42 (0.13-0.81) | -1.11 (-1.35--0.87) |
| France | 893 (213-1821) | 1.14 (0.27-2.32) | 869 (256-1682) | 0.66 (0.19-1.28) | -2.07 (-2.17--1.97) |
| Gabon | 8 (2-16) | 1.49 (0.31-2.91) | 14 (3-26) | 1.35 (0.33-2.61) | -0.54 (-0.71--0.38) |
| Gambia | 2 (1-3) | 0.63 (0.36-0.89) | 7 (4-11) | 0.81 (0.46-1.16) | 0.76 (0.59-0.92) |
| Georgia | 14 (2-34) | 0.24 (0.04-0.55) | 19 (5-36) | 0.32 (0.09-0.61) | 1.59 (1.38-1.79) |
| Germany | 469 (78-978) | 0.39 (0.06-0.81) | 942 (197-1949) | 0.52 (0.11-1.08) | 0.86 (0.57-1.16) |
| Ghana | 33 (10-65) | 0.54 (0.17-1.09) | 60 (12-115) | 0.4 (0.09-0.76) | -1.02 (-1.11--0.93) |
| Greece | 4 (1-12) | 0.03 (0.01-0.09) | 8 (1-23) | 0.03 (0.01-0.09) | -0.64 (-1.06--0.21) |
| Greenland | 1 (0-1) | 1.84 (0.4-3.95) | 1 (0-2) | 1.23 (0.22-2.9) | -1.8 (-2.05--1.54) |
| Grenada | 1 (0-1) | 1.01 (0.25-1.91) | 1 (0-2) | 0.9 (0.23-1.69) | -0.23 (-0.36--0.1) |
| Guam | 0 (0-0) | 0.41 (0.11-0.77) | 1 (0-1) | 0.35 (0.09-0.68) | -0.21 (-0.67-0.25) |
| Guatemala | 12 (5-21) | 0.38 (0.14-0.65) | 41 (13-81) | 0.39 (0.12-0.76) | -0.2 (-0.66-0.27) |
| Guinea | 7 (2-15) | 0.23 (0.05-0.46) | 17 (5-33) | 0.32 (0.09-0.63) | 1.4 (1.24-1.57) |
| Guinea-Bissau | 5 (2-9) | 1.36 (0.59-2.31) | 11 (4-20) | 1.58 (0.62-2.79) | 1.09 (0.86-1.31) |
| Guyana | 2 (1-3) | 0.52 (0.21-0.89) | 3 (1-5) | 0.45 (0.17-0.81) | -0.29 (-0.39--0.2) |
| Haiti | 24 (6-49) | 0.76 (0.21-1.53) | 50 (16-101) | 0.75 (0.24-1.5) | 0.06 (-0.04-0.15) |
| Honduras | 4 (1-8) | 0.22 (0.07-0.42) | 17 (4-34) | 0.3 (0.07-0.6) | 1.4 (1.22-1.58) |
| Hungary | 79 (14-171) | 0.56 (0.1-1.21) | 84 (19-188) | 0.47 (0.1-1.06) | -1.29 (-1.79--0.78) |
| Iceland | 3 (1-5) | 0.98 (0.4-1.65) | 3 (1-7) | 0.62 (0.17-1.22) | -1.84 (-2--1.67) |
| India | 6456 (3802-9246) | 1.5 (0.89-2.15) | 13044 (7251-19633) | 1.17 (0.65-1.76) | -1.18 (-1.34--1.02) |
| Indonesia | 734 (387-1073) | 0.77 (0.4-1.13) | 1016 (392-1787) | 0.51 (0.19-0.89) | -1.47 (-1.57--1.38) |
| Iran (Islamic Republic of) | 109 (16-225) | 0.49 (0.07-1) | 103 (19-244) | 0.16 (0.03-0.37) | -4.43 (-4.92--3.94) |
| Iraq | 8 (1-22) | 0.11 (0.01-0.29) | 34 (7-81) | 0.15 (0.03-0.36) | 0.53 (0.09-0.98) |
| Ireland | 65 (27-107) | 1.59 (0.66-2.61) | 59 (10-130) | 0.77 (0.13-1.72) | -2.89 (-3.12--2.65) |
| Israel | 2 (0-6) | 0.05 (0.01-0.14) | 4 (1-14) | 0.03 (0.01-0.12) | -1.15 (-1.38--0.91) |
| Italy | 129 (21-328) | 0.15 (0.02-0.38) | 180 (28-378) | 0.12 (0.02-0.26) | -0.6 (-0.76--0.44) |
| Jamaica | 7 (1-14) | 0.4 (0.07-0.8) | 11 (2-22) | 0.36 (0.08-0.75) | -0.22 (-0.52-0.09) |
| Japan | 1382 (475-2561) | 0.81 (0.28-1.51) | 1831 (580-3735) | 0.54 (0.18-1.07) | -1.14 (-1.34--0.94) |
| Jordan | 2 (0-5) | 0.17 (0.02-0.39) | 9 (2-20) | 0.15 (0.03-0.32) | -1.02 (-1.24--0.8) |
| Kazakhstan | 660 (304-987) | 5.45 (2.55-8.11) | 143 (32-345) | 0.9 (0.21-2.15) | -6.96 (-7.28--6.63) |
| Kenya | 201 (89-339) | 2.52 (1.12-4.26) | 616 (254-1062) | 2.96 (1.22-5.16) | 0.95 (0.73-1.16) |
| Kiribati | 1 (0-1) | 1.82 (0.63-3.29) | 1 (0-2) | 1.65 (0.51-3.07) | -0.55 (-0.71--0.39) |
| Kuwait | 1 (0-2) | 0.13 (0.01-0.36) | 2 (0-7) | 0.11 (0.01-0.32) | -0.58 (-0.87--0.28) |
| Kyrgyzstan | 57 (24-90) | 1.88 (0.8-2.99) | 31 (11-59) | 0.74 (0.25-1.4) | -3.91 (-4.15--3.66) |
| Lao People's Democratic Republic | 25 (12-40) | 1.19 (0.56-1.92) | 17 (4-35) | 0.4 (0.1-0.84) | -4.35 (-4.58--4.11) |
| Latvia | 21 (8-36) | 0.59 (0.21-1.01) | 24 (9-42) | 0.66 (0.24-1.15) | 0.27 (0.01-0.54) |
| Lebanon | 0 (0-0) | 0.01 (0.01-0.02) | 1 (0-4) | 0.02 (0-0.08) | 3.27 (2.87-3.68) |
| Lesotho | 40 (21-61) | 4.15 (2.13-6.21) | 64 (32-99) | 5.11 (2.61-7.84) | 1.01 (0.75-1.26) |
| Liberia | 10 (4-17) | 0.92 (0.38-1.59) | 26 (11-44) | 1.34 (0.57-2.27) | 1.77 (1.46-2.08) |
| Libya | 2 (0-4) | 0.09 (0.01-0.22) | 7 (1-16) | 0.13 (0.01-0.32) | 1.02 (0.56-1.48) |
| Lithuania | 26 (9-46) | 0.57 (0.2-1.02) | 30 (8-60) | 0.58 (0.15-1.14) | 0.08 (-0.17-0.33) |
| Luxembourg | 4 (1-7) | 0.71 (0.16-1.4) | 4 (1-9) | 0.44 (0.11-0.9) | -1.62 (-1.67--1.57) |
| Madagascar | 153 (62-266) | 3 (1.2-5.18) | 291 (125-503) | 2.7 (1.16-4.63) | -0.62 (-0.86--0.38) |
| Malawi | 219 (96-364) | 5.75 (2.51-9.51) | 406 (149-715) | 5.66 (2.11-9.74) | -0.12 (-0.34-0.1) |
| Malaysia | 62 (28-99) | 0.71 (0.32-1.13) | 99 (25-205) | 0.4 (0.11-0.82) | -2.59 (-2.86--2.32) |
| Maldives | 1 (0-2) | 0.99 (0.35-1.85) | 1 (0-2) | 0.38 (0.14-0.68) | -3.99 (-4.27--3.71) |
| Mali | 32 (16-46) | 0.78 (0.4-1.14) | 59 (27-96) | 0.7 (0.33-1.14) | -0.39 (-0.46--0.33) |
| Malta | 2 (0-3) | 0.36 (0.06-0.8) | 2 (0-5) | 0.2 (0.03-0.51) | -1.64 (-1.98--1.3) |
| Marshall Islands | 0 (0-0) | 0.79 (0.33-1.43) | 0 (0-0) | 0.67 (0.29-1.2) | -0.52 (-0.61--0.44) |
| Mauritania | 15 (9-22) | 1.56 (0.9-2.27) | 30 (15-46) | 1.53 (0.78-2.34) | 0.39 (0.17-0.62) |
| Mauritius | 7 (4-10) | 1.07 (0.62-1.49) | 11 (5-17) | 0.64 (0.3-1.02) | -1.92 (-2.05--1.79) |
| Mexico | 120 (33-222) | 0.3 (0.08-0.57) | 215 (54-400) | 0.19 (0.05-0.35) | -1.75 (-2.01--1.48) |
| Micronesia (Federated States of) | 0 (0-1) | 0.89 (0.39-1.52) | 0 (0-1) | 0.77 (0.32-1.31) | -0.69 (-0.76--0.63) |
| Monaco | 0 (0-0) | 0.17 (0.02-0.48) | 0 (0-0) | 0.1 (0.02-0.35) | -1.15 (-1.43--0.87) |
| Mongolia | 94 (53-136) | 9.99 (5.3-14.52) | 149 (82-224) | 8.21 (4.32-12.24) | -1.14 (-1.4--0.87) |
| Montenegro | 1 (0-2) | 0.08 (0.01-0.29) | 0 (0-2) | 0.05 (0.01-0.17) | -3.79 (-4.59--2.98) |
| Morocco | 29 (6-62) | 0.22 (0.05-0.47) | 24 (3-58) | 0.08 (0.01-0.2) | -4.12 (-4.76--3.48) |
| Mozambique | 126 (69-187) | 2.25 (1.2-3.33) | 265 (136-416) | 2.57 (1.31-3.98) | 0.72 (0.6-0.84) |
| Myanmar | 262 (133-411) | 1.14 (0.59-1.77) | 265 (117-436) | 0.59 (0.26-0.97) | -2.51 (-2.6--2.42) |
| Namibia | 6 (3-9) | 0.82 (0.44-1.2) | 12 (6-18) | 0.83 (0.43-1.26) | -0.28 (-0.59-0.04) |
| Nauru | 0 (0-0) | 0.67 (0.22-1.27) | 0 (0-0) | 0.64 (0.22-1.17) | -0.3 (-0.88-0.28) |
| Nepal | 147 (75-229) | 1.58 (0.79-2.45) | 247 (103-432) | 1.16 (0.49-2.02) | -0.99 (-1.36--0.63) |
| Netherlands | 127 (30-252) | 0.64 (0.15-1.27) | 308 (58-627) | 0.9 (0.17-1.84) | 1.2 (0.9-1.49) |
| New Zealand | 22 (3-47) | 0.55 (0.08-1.2) | 37 (6-77) | 0.46 (0.07-0.96) | -0.56 (-0.65--0.48) |
| Nicaragua | 5 (2-7) | 0.33 (0.16-0.51) | 16 (8-25) | 0.4 (0.21-0.62) | 0.08 (-0.27-0.43) |
| Niger | 32 (18-47) | 1.2 (0.67-1.73) | 89 (41-141) | 1.24 (0.57-1.96) | 0.41 (0.24-0.57) |
| Nigeria | 81 (29-176) | 0.19 (0.07-0.41) | 146 (52-307) | 0.19 (0.07-0.38) | -0.08 (-0.21-0.06) |
| Niue | 0 (0-0) | 0.55 (0.2-1) | 0 (0-0) | 0.46 (0.16-0.87) | -0.89 (-1.07--0.72) |
| North Macedonia | 2 (0-6) | 0.1 (0.01-0.31) | 2 (0-8) | 0.08 (0.01-0.25) | -1.54 (-1.88--1.2) |
| Northern Mariana Islands | 0 (0-0) | 0.23 (0.06-0.5) | 0 (0-0) | 0.45 (0.13-0.87) | 3.4 (2.94-3.85) |
| Norway | 28 (9-51) | 0.41 (0.13-0.76) | 31 (8-58) | 0.32 (0.08-0.6) | -0.97 (-1.05--0.9) |
| Oman | 1 (0-3) | 0.19 (0.02-0.43) | 2 (0-5) | 0.15 (0.02-0.32) | -0.77 (-0.99--0.54) |
| Pakistan | 1096 (540-1719) | 1.95 (0.95-3.06) | 2360 (1154-3798) | 2.16 (1.05-3.44) | 0.33 (0.1-0.55) |
| Palau | 0 (0-0) | 0.47 (0.17-0.87) | 0 (0-0) | 0.45 (0.15-0.82) | -0.33 (-0.43--0.24) |
| Palestine | 2 (1-4) | 0.3 (0.1-0.52) | 3 (1-7) | 0.15 (0.03-0.32) | -3.05 (-3.37--2.72) |
| Panama | 5 (2-9) | 0.34 (0.12-0.61) | 12 (4-24) | 0.3 (0.09-0.57) | -0.1 (-0.36-0.16) |
| Papua New Guinea | 8 (3-15) | 0.47 (0.2-0.88) | 19 (8-36) | 0.45 (0.18-0.84) | -0.12 (-0.19--0.04) |
| Paraguay | 9 (2-17) | 0.41 (0.09-0.79) | 29 (6-58) | 0.53 (0.11-1.08) | 0.81 (0.48-1.15) |
| Peru | 45 (17-77) | 0.4 (0.15-0.68) | 68 (18-138) | 0.21 (0.06-0.43) | -2.3 (-2.44--2.16) |
| Philippines | 78 (27-140) | 0.27 (0.09-0.49) | 154 (47-302) | 0.2 (0.06-0.39) | -1.22 (-1.35--1.08) |
| Poland | 294 (109-519) | 0.68 (0.26-1.2) | 287 (79-610) | 0.42 (0.12-0.9) | -1.93 (-2.1--1.77) |
| Portugal | 55 (6-145) | 0.41 (0.05-1.08) | 46 (5-130) | 0.21 (0.02-0.59) | -1.68 (-1.97--1.39) |
| Puerto Rico | 47 (17-84) | 1.31 (0.48-2.33) | 26 (7-51) | 0.36 (0.09-0.72) | -5.18 (-5.55--4.8) |
| Qatar | 0 (0-0) | 0.12 (0.02-0.35) | 0 (0-1) | 0.07 (0.02-0.23) | -3.09 (-3.55--2.64) |
| Republic of Korea | 198 (48-459) | 0.69 (0.18-1.54) | 215 (38-542) | 0.24 (0.04-0.61) | -4.24 (-4.55--3.92) |
| Republic of Moldova | 14 (3-32) | 0.32 (0.07-0.72) | 18 (5-35) | 0.31 (0.09-0.61) | -0.2 (-0.42-0.03) |
| Romania | 47 (8-116) | 0.17 (0.03-0.41) | 61 (7-192) | 0.18 (0.02-0.56) | -0.38 (-0.75--0.01) |
| Russian Federation | 1834 (763-3056) | 1.01 (0.42-1.68) | 1250 (408-2330) | 0.53 (0.18-0.99) | -2.67 (-2.95--2.38) |
| Rwanda | 64 (12-124) | 2.23 (0.41-4.3) | 67 (13-136) | 1.18 (0.24-2.36) | -3.15 (-3.51--2.8) |
| Saint Kitts and Nevis | 1 (0-1) | 1.59 (0.85-2.26) | 1 (0-1) | 1.15 (0.55-1.75) | -1.13 (-1.47--0.79) |
| Saint Lucia | 1 (0-1) | 0.68 (0.17-1.23) | 2 (0-3) | 0.71 (0.2-1.33) | 0.21 (0.01-0.42) |
| Saint Vincent and the Grenadines | 0 (0-1) | 0.48 (0.18-0.85) | 0 (0-1) | 0.32 (0.07-0.61) | -1.77 (-2.26--1.28) |
| Samoa | 0 (0-1) | 0.52 (0.23-0.87) | 1 (0-1) | 0.37 (0.16-0.64) | -1.46 (-1.56--1.36) |
| San Marino | 0 (0-0) | 0.16 (0.02-0.35) | 0 (0-0) | 0.14 (0.02-0.35) | -0.48 (-0.92--0.04) |
| Sao Tome and Principe | 0 (0-1) | 0.53 (0.17-1.02) | 1 (0-1) | 0.65 (0.17-1.3) | 0.93 (0.69-1.17) |
| Saudi Arabia | 9 (1-22) | 0.17 (0.02-0.4) | 35 (6-78) | 0.21 (0.03-0.46) | 0.54 (0.07-1.01) |
| Senegal | 38 (20-57) | 1.22 (0.65-1.83) | 102 (48-161) | 1.43 (0.67-2.24) | 1.15 (0.81-1.5) |
| Serbia | 30 (4-70) | 0.26 (0.03-0.6) | 29 (3-70) | 0.19 (0.02-0.47) | -1.65 (-1.95--1.36) |
| Seychelles | 1 (0-1) | 1.27 (0.55-2.07) | 1 (0-2) | 1.02 (0.35-1.87) | -0.9 (-1.04--0.76) |
| Sierra Leone | 18 (8-28) | 0.96 (0.45-1.48) | 42 (18-70) | 1.22 (0.52-2.04) | 1.38 (1.15-1.61) |
| Singapore | 14 (3-31) | 0.7 (0.13-1.53) | 9 (1-23) | 0.12 (0.01-0.31) | -6.08 (-6.42--5.73) |
| Slovakia | 41 (15-74) | 0.7 (0.25-1.25) | 47 (14-97) | 0.52 (0.15-1.09) | -1.34 (-1.58--1.1) |
| Slovenia | 11 (1-24) | 0.44 (0.06-0.98) | 11 (2-25) | 0.27 (0.04-0.6) | -2.22 (-2.48--1.97) |
| Solomon Islands | 1 (1-2) | 0.98 (0.46-1.67) | 3 (1-5) | 0.87 (0.38-1.5) | -0.57 (-0.72--0.41) |
| Somalia | 136 (60-224) | 5.28 (2.37-8.52) | 284 (143-475) | 4.36 (2.2-7.29) | -0.66 (-0.75--0.57) |
| South Africa | 822 (420-1264) | 3.94 (2.01-6.11) | 1263 (667-1883) | 2.96 (1.56-4.39) | -1.69 (-2.34--1.03) |
| South Sudan | 61 (20-117) | 2.61 (0.86-5.05) | 71 (23-146) | 1.92 (0.61-3.85) | -1.3 (-1.4--1.21) |
| Spain | 69 (9-218) | 0.13 (0.02-0.42) | 150 (15-348) | 0.16 (0.02-0.37) | 0.9 (0.77-1.04) |
| Sri Lanka | 138 (68-212) | 1.35 (0.65-2.1) | 284 (131-483) | 1.14 (0.53-1.94) | 0.04 (-0.45-0.53) |
| Sudan | 77 (19-157) | 0.86 (0.21-1.76) | 140 (29-308) | 0.8 (0.16-1.76) | -0.42 (-0.54--0.3) |
| Suriname | 1 (0-1) | 0.26 (0.08-0.49) | 1 (0-3) | 0.23 (0.06-0.46) | -0.33 (-0.61--0.04) |
| Sweden | 70 (22-132) | 0.46 (0.15-0.86) | 79 (20-155) | 0.36 (0.09-0.72) | -1.08 (-1.19--0.96) |
| Switzerland | 63 (11-129) | 0.61 (0.11-1.27) | 74 (13-153) | 0.42 (0.07-0.88) | -1.19 (-1.25--1.12) |
| Syrian Arab Republic | 3 (0-8) | 0.06 (0.01-0.16) | 12 (2-27) | 0.11 (0.01-0.23) | 1.07 (0.77-1.37) |
| Taiwan (Province of China) | 106 (16-227) | 0.67 (0.1-1.43) | 183 (19-493) | 0.48 (0.05-1.29) | -0.81 (-1--0.62) |
| Tajikistan | 67 (25-115) | 2.43 (0.9-4.16) | 60 (19-142) | 1.38 (0.44-3.38) | -2.24 (-3.03--1.45) |
| Thailand | 251 (77-482) | 0.72 (0.22-1.4) | 381 (84-807) | 0.37 (0.08-0.78) | -2.93 (-3.21--2.66) |
| Timor-Leste | 2 (1-4) | 0.88 (0.48-1.33) | 6 (3-10) | 0.8 (0.4-1.3) | -0.38 (-0.61--0.16) |
| Togo | 16 (9-22) | 1.32 (0.76-1.87) | 61 (31-97) | 1.74 (0.87-2.7) | 1.37 (1.2-1.54) |
| Tokelau | 0 (0-0) | 0.58 (0.25-0.97) | 0 (0-0) | 0.41 (0.16-0.75) | -1.27 (-1.3--1.23) |
| Tonga | 0 (0-0) | 0.52 (0.23-0.89) | 0 (0-1) | 0.48 (0.2-0.85) | -0.53 (-0.84--0.21) |
| Trinidad and Tobago | 5 (2-8) | 0.6 (0.27-0.95) | 6 (3-11) | 0.35 (0.14-0.61) | -2.71 (-3.1--2.31) |
| Tunisia | 5 (1-11) | 0.1 (0.01-0.24) | 6 (1-18) | 0.05 (0.01-0.15) | -3.13 (-3.37--2.89) |
| Turkey | 16 (3-65) | 0.04 (0.01-0.17) | 14 (5-49) | 0.02 (0.01-0.06) | -3.8 (-4.06--3.55) |
| Turkmenistan | 91 (22-190) | 4.88 (1.19-10.22) | 24 (3-76) | 0.64 (0.07-2.03) | -7.96 (-8.49--7.42) |
| Tuvalu | 0 (0-0) | 0.8 (0.37-1.29) | 0 (0-0) | 0.58 (0.25-1.03) | -1.25 (-1.33--1.18) |
| Uganda | 100 (24-190) | 1.62 (0.39-3.07) | 275 (66-510) | 2.02 (0.48-3.77) | 0.81 (0.65-0.97) |
| Ukraine | 465 (167-819) | 0.65 (0.24-1.14) | 349 (117-664) | 0.48 (0.16-0.91) | -2.32 (-2.8--1.83) |
| United Arab Emirates | 0 (0-2) | 0.09 (0.02-0.33) | 30 (3-80) | 0.66 (0.06-1.74) | 7.8 (6.87-8.74) |
| United Kingdom | 1379 (603-2279) | 1.51 (0.66-2.49) | 1775 (662-3060) | 1.37 (0.51-2.39) | -0.58 (-0.68--0.47) |
| United Republic of Tanzania | 426 (184-736) | 4 (1.77-6.78) | 533 (166-1013) | 2.25 (0.71-4.24) | -2.39 (-2.54--2.24) |
| United States of America | 1556 (382-3248) | 0.5 (0.12-1.04) | 2772 (704-5613) | 0.5 (0.13-1.01) | 0.17 (0.11-0.24) |
| United States Virgin Islands | 0 (0-1) | 0.48 (0.13-0.91) | 1 (0-2) | 0.44 (0.07-0.85) | 0.12 (-0.24-0.48) |
| Uruguay | 71 (23-128) | 1.82 (0.6-3.29) | 51 (12-98) | 0.91 (0.22-1.78) | -2.46 (-2.6--2.31) |
| Uzbekistan | 313 (103-579) | 2.82 (0.92-5.25) | 100 (12-301) | 0.55 (0.07-1.61) | -7.13 (-7.73--6.53) |
| Vanuatu | 0 (0-1) | 0.44 (0.11-0.91) | 1 (0-2) | 0.49 (0.16-1) | 0.29 (0.2-0.39) |
| Venezuela (Bolivarian Republic of) | 34 (9-64) | 0.37 (0.1-0.71) | 82 (18-167) | 0.29 (0.06-0.59) | -1.2 (-1.41--0.99) |
| Viet Nam | 238 (110-388) | 0.6 (0.28-0.97) | 409 (100-867) | 0.43 (0.11-0.9) | -1.04 (-1.1--0.97) |
| Yemen | 54 (16-96) | 1.17 (0.35-2.08) | 142 (39-253) | 1.14 (0.31-2.04) | -0.41 (-0.53--0.28) |
| Zambia | 184 (99-266) | 6.56 (3.55-9.45) | 344 (187-543) | 5.24 (2.85-8.06) | -1.19 (-1.45--0.93) |
| Zimbabwe | 216 (126-299) | 5.51 (3.22-7.57) | 381 (210-548) | 5.62 (3.15-8.02) | 0 (-0.24-0.23) |

**Table S2.** The number of DALYs cases and the age-standardized DALYs rate of esophageal cancer attributable to dietary risks in 1990 and 2019, and its trends from 1990 to 2019 globally. Abbreviations: DALYs, disability-adjusted life years.

|  | Number of DALYs  cases (95% UI) in 1990 | The age-standardized DALYs rate/100000 (95% UI) in 1990 | Number of DALYs  cases (95% UI) in 2019 | The age-standardized DALYs rate/100000 (95% UI) in 2019 | EAPC (95% CI) |
| --- | --- | --- | --- | --- | --- |
| Global | 1814044 (844389-2918905) | 43.94 (20.34-70.71) | 1606568 (668474-2913673) | 19.24 (7.99-34.88) | -3.22 (-3.44--3) |
| **Sex** |  |  |  |  |  |
| Female | 574238 (273020-919564) | 26.62 (12.69-42.65) | 465581 (220388-771555) | 10.73 (5.09-17.73) | -3.61 (-3.86--3.36) |
| Male | 1239806 (567580-2005075) | 62.89 (28.77-101.7) | 1140987 (446881-2199604) | 28.54 (11.16-55.18) | -3.04 (-3.25--2.83) |
| **Age** |  |  |  |  |  |
| <24 years | 0 (0-0) | 0 (0-0) | 0 (0-0) | 0 (0-0) | 0 (0-0) |
| 25-29 years | 11345 (6195-16576) | 2.56 (1.4-3.74) | 10073 (5336-15210) | 1.66 (0.88-2.51) | -2.12 (-2.42--1.83) |
| 30-34 years | 20347 (10437-30056) | 5.28 (2.71-7.79) | 20038 (10030-30968) | 3.33 (1.67-5.15) | -2.15 (-2.47--1.83) |
| 35-39 years | 47112 (23751-70016) | 13.36 (6.73-19.85) | 39050 (19160-61531) | 7.22 (3.54-11.37) | -2.43 (-2.73--2.13) |
| 40-44 years | 101010 (50518-156992) | 35.28 (17.65-54.84) | 78085 (37598-126571) | 15.82 (7.62-25.65) | -3.36 (-3.61--3.12) |
| 45-49 years | 143703 (69732-224611) | 61.83 (30-96.64) | 134532 (60261-229673) | 28.39 (12.72-48.47) | -3.22 (-3.52--2.92) |
| 50-54 years | 226589 (109420-355593) | 106.58 (51.47-167.27) | 204071 (89694-361771) | 46.72 (20.53-82.82) | -3.28 (-3.61--2.95) |
| 55-59 years | 288841 (130526-468895) | 155.78 (70.4-252.89) | 239750 (101654-430304) | 64.62 (27.4-115.98) | -3.25 (-3.44--3.07) |
| 60-64 years | 301349 (133723-496028) | 187.59 (83.24-308.78) | 233956 (93096-452651) | 74.86 (29.79-144.83) | -3.41 (-3.55--3.27) |
| 65-69 years | 268141 (118082-444260) | 217.14 (95.62-359.76) | 223217 (84913-438811) | 86.32 (32.84-169.7) | -3.51 (-3.69--3.33) |
| 70-74 years | 204342 (87986-333779) | 241.79 (104.11-394.94) | 180886 (67982-360372) | 96.69 (36.34-192.62) | -3.55 (-3.79--3.3) |
| 75-79 years | 123347 (57175-201162) | 201.19 (93.26-328.11) | 118167 (44829-231349) | 93.01 (35.28-182.09) | -3.26 (-3.55--2.97) |
| 80-84 years | 56401 (26737-91000) | 160.14 (75.92-258.38) | 75907 (27704-148705) | 89.91 (32.82-176.14) | -2.31 (-2.65--1.97) |
| 85-89 years | 17584 (7648-29090) | 116.69 (50.75-193.05) | 37145 (12630-72579) | 85.43 (29.05-166.92) | -1.25 (-1.57--0.93) |
| 90-94 years | 3390 (1416-5542) | 76.93 (32.14-125.78) | 9699 (3662-17916) | 57.53 (21.72-106.28) | -1.11 (-1.31--0.91) |
| 95+ years | 543 (228-920) | 52.71 (22.14-89.33) | 1993 (696-3729) | 41.76 (14.59-78.12) | -0.69 (-0.77--0.62) |
| **SDI regions** |  |  |  |  |  |
| High SDI | 171890 (55377-331820) | 17.11 (5.48-33.03) | 216350 (63803-426442) | 12.55 (3.74-24.82) | -1.1 (-1.16--1.04) |
| High-middle SDI | 438205 (188987-730034) | 39.81 (17.16-66.29) | 284294 (88079-640007) | 13.92 (4.34-31.24) | -4.1 (-4.38--3.81) |
| Middle SDI | 843811 (375628-1364160) | 77.84 (34.66-126.38) | 504333 (180363-1040821) | 19.65 (7.04-40.54) | -5.2 (-5.58--4.81) |
| Low-middle SDI | 253284 (141144-372674) | 38.6 (21.42-56.76) | 401828 (214114-615170) | 27.59 (14.7-42.26) | -1.35 (-1.45--1.25) |
| Low SDI | 106509 (55341-159359) | 40.59 (21.06-60.44) | 199245 (102378-305090) | 34.8 (17.79-52.98) | -0.66 (-0.75--0.57) |
| **GBD regions** |  |  |  |  |  |
| Africa | 125725 (63084-193587) | 40.11 (20.03-61.94) | 222579 (109228-341789) | 32.14 (15.65-49.18) | -1.07 (-1.28--0.85) |
| African Region | 117496 (59367-178710) | 47.94 (24.19-72.98) | 208092 (101701-320980) | 38.68 (18.86-59.38) | -1.05 (-1.29--0.82) |
| America | 90597 (25179-178116) | 14.95 (4.14-29.4) | 134335 (34686-261277) | 10.71 (2.77-20.81) | -1.19 (-1.26--1.11) |
| Andean Latin America | 1715 (631-2937) | 8.09 (2.95-13.89) | 2756 (772-5433) | 4.89 (1.37-9.58) | -1.76 (-1.85--1.67) |
| Asia | 1429910 (679173-2264057) | 66.68 (31.48-105.82) | 1089063 (440799-2069011) | 21.96 (8.85-41.86) | -4.22 (-4.49--3.94) |
| Australasia | 3690 (1108-7043) | 15.85 (4.73-30.31) | 5724 (1345-11849) | 12.26 (2.88-25.34) | -0.94 (-0.99--0.9) |
| Caribbean | 3838 (1215-7075) | 14.58 (4.58-26.78) | 5461 (1475-10858) | 10.5 (2.85-20.82) | -1.27 (-1.53--1.01) |
| Central Asia | 34577 (14713-56822) | 71.78 (30.67-117.42) | 14729 (4536-32242) | 19.05 (6.08-41.6) | -5.47 (-5.79--5.15) |
| Central Europe | 17392 (5454-34056) | 11.86 (3.75-23.09) | 16936 (4582-37571) | 8.86 (2.43-19.64) | -1.37 (-1.59--1.16) |
| Central Latin America | 7292 (2089-13508) | 8.41 (2.37-15.66) | 12405 (3257-23722) | 5.19 (1.36-9.9) | -1.88 (-2.02--1.73) |
| Central Sub-Saharan Africa | 15899 (5766-28827) | 62.34 (22.49-112.11) | 31548 (13266-53668) | 52.95 (22.16-89.61) | -0.88 (-1.11--0.65) |
| Commonwealth High Income | 35619 (14416-61856) | 24.35 (9.82-42.29) | 45914 (15550-84923) | 18.91 (6.35-34.79) | -1.1 (-1.19--1.01) |
| Commonwealth Low Income | 60795 (32889-88805) | 64.32 (34.71-94.2) | 102815 (45651-168909) | 43.52 (19.39-71.08) | -1.39 (-1.45--1.33) |
| Commonwealth Middle Income | 259355 (151452-356874) | 37.77 (22.12-52.04) | 486699 (269308-705548) | 30.46 (16.88-44.22) | -1.04 (-1.23--0.85) |
| East Asia | 1042998 (451943-1713656) | 113.28 (49.56-185.91) | 471627 (100155-1199137) | 22.22 (4.85-55.88) | -6.04 (-6.53--5.55) |
| East Asia & Pacific - WB | 1135381 (506733-1862270) | 79.99 (35.75-131.42) | 586416 (148313-1392294) | 18.38 (4.66-43.57) | -5.47 (-5.88--5.05) |
| Eastern Europe | 64956 (25817-109410) | 22.81 (9.07-38.26) | 45309 (15430-84751) | 13.76 (4.78-25.53) | -2.42 (-2.71--2.13) |
| Eastern Mediterranean Region | 47295 (22184-75485) | 23.92 (11.13-38.23) | 100201 (48357-161615) | 20.48 (9.86-33.12) | -0.64 (-0.78--0.49) |
| Eastern Sub-Saharan Africa | 60891 (30801-92817) | 73.31 (37.08-111.48) | 106368 (50877-167924) | 58.37 (27.47-92.13) | -0.95 (-1.03--0.87) |
| Europe | 166396 (60194-303713) | 16.48 (6-30.09) | 159490 (49982-306264) | 11.3 (3.54-21.72) | -1.69 (-1.82--1.56) |
| Europe & Central Asia - WB | 196676 (72896-353474) | 18.9 (7.03-33.93) | 168936 (52133-329237) | 11.54 (3.58-22.52) | -2.13 (-2.27--1.99) |
| European Region | 196779 (72908-353824) | 18.78 (6.98-33.74) | 169080 (52157-329461) | 11.44 (3.55-22.33) | -2.14 (-2.29--2) |
| High-income Asia Pacific | 38070 (12914-72986) | 18.39 (6.23-35.18) | 39529 (12595-81813) | 10.2 (3.38-20.46) | -1.89 (-2.08--1.69) |
| High-income North America | 39538 (9733-83268) | 12.03 (2.94-25.33) | 66409 (17292-136529) | 11.27 (2.96-23.16) | -0.08 (-0.14--0.03) |
| Latin America & Caribbean - WB | 52130 (15977-95081) | 18.74 (5.69-34.23) | 68485 (16445-129479) | 10.09 (2.42-19.02) | -2.32 (-2.45--2.2) |
| Middle East & North Africa - WB | 7932 (2217-15803) | 5.89 (1.63-11.61) | 13208 (4115-27611) | 3.68 (1.14-7.68) | -1.98 (-2.26--1.69) |
| North Africa and Middle East | 14250 (4505-27789) | 7.63 (2.39-14.75) | 23167 (7624-46330) | 4.78 (1.57-9.62) | -1.99 (-2.17--1.81) |
| North America | 39527 (9730-83249) | 12.03 (2.94-25.33) | 66406 (17291-136516) | 11.27 (2.96-23.15) | -0.08 (-0.14--0.03) |
| Oceania | 449 (204-765) | 13.52 (6.11-23.16) | 971 (423-1695) | 12.25 (5.25-21.22) | -0.28 (-0.33--0.23) |
| Region of the Americas | 90597 (25179-178116) | 14.95 (4.14-29.4) | 134335 (34686-261277) | 10.71 (2.77-20.81) | -1.19 (-1.26--1.11) |
| South-East Asia Region | 266417 (155372-373844) | 34.25 (19.8-48.07) | 462458 (254255-687946) | 25.28 (13.87-37.5) | -1.24 (-1.35--1.14) |
| South Asia | 251224 (147981-346357) | 39.79 (23.27-54.4) | 469409 (256992-678615) | 31.28 (17.18-45.33) | -1.02 (-1.14--0.91) |
| South Asia - WB | 258921 (151519-356834) | 39.79 (23.12-54.62) | 482950 (265213-701005) | 31.31 (17.22-45.55) | -1.01 (-1.13--0.9) |
| Southeast Asia | 52061 (26182-78467) | 18.54 (9.23-28.2) | 72259 (27674-126811) | 11.07 (4.25-19.47) | -1.89 (-1.94--1.84) |
| Southern Latin America | 12345 (3139-24059) | 26.65 (6.79-51.88) | 8591 (1634-17496) | 10.43 (1.98-21.25) | -3.66 (-3.85--3.47) |
| Southern Sub-Saharan Africa | 31970 (17262-46844) | 106.55 (56.94-156.95) | 47441 (25423-68451) | 79.05 (42.23-113.62) | -1.64 (-2.2--1.07) |
| Sub-Saharan Africa - WB | 123078 (62349-188111) | 50.2 (25.31-77.15) | 219587 (108379-335921) | 41.47 (20.3-63.49) | -0.95 (-1.18--0.72) |
| Tropical Latin America | 27077 (8577-48866) | 27.37 (8.67-49.39) | 39477 (9248-74668) | 15.76 (3.68-29.71) | -2.04 (-2.13--1.96) |
| Western Europe | 81535 (26066-154983) | 15.2 (4.81-28.96) | 95416 (27424-181645) | 11.94 (3.42-22.8) | -1.01 (-1.1--0.92) |
| Western Pacific Region | 1092208 (480132-1794549) | 88.85 (39.55-145.89) | 529557 (125234-1299116) | 19.16 (4.56-46.85) | -5.69 (-6.14--5.25) |
| Western Sub-Saharan Africa | 12277 (6205-18929) | 13.09 (6.58-20.18) | 31037 (15191-48815) | 15.25 (7.45-24.12) | 0.78 (0.68-0.89) |
| World Bank High Income | 186212 (59379-359466) | 15.1 (4.8-29.17) | 233158 (67758-461125) | 11.36 (3.33-22.49) | -1.03 (-1.1--0.96) |
| World Bank Low Income | 88106 (42697-139070) | 53.82 (26.34-85.24) | 159063 (73998-250928) | 46.13 (21.43-72.85) | -0.66 (-0.75--0.57) |
| World Bank Lower Middle Income | 346928 (194866-487928) | 30.39 (17.02-42.97) | 604279 (328090-877847) | 23.7 (12.86-34.44) | -1.08 (-1.18--0.97) |
| World Bank Upper Middle Income | 1192450 (532055-1963944) | 75.13 (33.31-123.86) | 609545 (161783-1439780) | 17.68 (4.79-41.27) | -5.45 (-5.87--5.03) |
| **Countries** |  |  |  |  |  |
| Afghanistan | 3990 (1158-7297) | 52.75 (15.39-95.41) | 6678 (1858-12313) | 44.76 (12.77-80.55) | -0.8 (-1.09--0.5) |
| Albania | 170 (40-354) | 7.62 (1.73-16.07) | 27 (6-102) | 0.72 (0.14-2.66) | -9.32 (-10.04--8.59) |
| Algeria | 743 (248-1393) | 5.9 (1.95-11.11) | 749 (89-1691) | 2.18 (0.28-4.9) | -4.13 (-4.41--3.86) |
| American Samoa | 1 (0-2) | 5.12 (1.75-9.38) | 3 (1-5) | 5.99 (2.09-10.96) | 0.62 (0.45-0.79) |
| Andorra | 5 (1-13) | 9.38 (1.51-21.85) | 11 (2-25) | 7.57 (1.09-17.83) | -1.06 (-1.21--0.91) |
| Angola | 3852 (1557-6529) | 85.43 (34.65-142.81) | 4888 (1575-9411) | 38.71 (12.48-74.72) | -3.53 (-3.85--3.21) |
| Antigua and Barbuda | 5 (1-10) | 9.67 (1.66-19.22) | 9 (2-18) | 8.72 (1.82-17.32) | -0.23 (-0.46--0.01) |
| Argentina | 7945 (1679-16233) | 24.55 (5.3-49.79) | 5439 (714-11048) | 10.3 (1.36-21.08) | -3.42 (-3.64--3.2) |
| Armenia | 145 (20-401) | 5.14 (0.71-14.12) | 50 (7-182) | 1.24 (0.16-4.45) | -6.27 (-6.72--5.81) |
| Australia | 3241 (1037-6077) | 16.7 (5.38-31.29) | 5017 (1201-10288) | 12.79 (3.05-26.3) | -0.99 (-1.04--0.94) |
| Austria | 1118 (187-2330) | 10.53 (1.76-21.93) | 811 (81-1832) | 5.21 (0.52-11.89) | -2.96 (-3.2--2.72) |
| Azerbaijan | 1551 (236-3723) | 29.1 (4.39-69.73) | 1074 (115-3873) | 10.82 (1.18-38.18) | -5 (-5.74--4.27) |
| Bahamas | 35 (7-77) | 21.09 (4.16-46.46) | 74 (18-151) | 17.5 (4.27-35.71) | -0.88 (-0.98--0.78) |
| Bahrain | 10 (1-24) | 5.22 (0.64-13.25) | 17 (3-44) | 1.56 (0.24-4.06) | -4.99 (-5.31--4.66) |
| Bangladesh | 28713 (16260-41156) | 56.68 (32.41-81.38) | 41319 (20593-68288) | 30.27 (15.18-49.96) | -2.1 (-2.21--1.98) |
| Barbados | 95 (47-144) | 35.89 (17.53-54.61) | 120 (50-207) | 25.21 (10.41-43.38) | -1.47 (-1.69--1.24) |
| Belarus | 2302 (884-3917) | 17.8 (7-29.97) | 1430 (272-3667) | 9.36 (1.86-23.73) | -3.54 (-4.01--3.07) |
| Belgium | 2168 (604-4306) | 15.4 (4.32-30.45) | 2068 (284-4950) | 10.31 (1.39-25.02) | -1.75 (-1.89--1.61) |
| Belize | 5 (1-9) | 4.96 (0.96-9.25) | 18 (3-35) | 6 (1.1-11.43) | 0.63 (0.34-0.91) |
| Benin | 549 (282-822) | 26.61 (13.74-40.03) | 1657 (711-2813) | 31.56 (13.55-53.04) | 0.93 (0.75-1.11) |
| Bermuda | 9 (1-21) | 13.93 (1.49-33.1) | 19 (6-37) | 15.78 (4.97-30.71) | 0.86 (0.69-1.03) |
| Bhutan | 119 (56-188) | 42.59 (20.7-66.61) | 159 (66-284) | 27.13 (11.15-48.02) | -1.58 (-1.72--1.44) |
| Bolivia (Plurinational State of) | 333 (87-689) | 9.96 (2.5-20.47) | 800 (224-1549) | 8.96 (2.51-17.3) | -0.23 (-0.3--0.15) |
| Bosnia and Herzegovina | 587 (238-980) | 13.22 (5.34-22.09) | 234 (29-676) | 4.08 (0.52-11.74) | -5.24 (-5.66--4.82) |
| Botswana | 757 (361-1228) | 121.8 (58.74-194.8) | 1648 (807-2628) | 105.73 (53.05-165.42) | -1.17 (-1.66--0.68) |
| Brazil | 26872 (8531-48478) | 27.82 (8.83-50.12) | 38781 (9096-73282) | 15.85 (3.7-29.88) | -2.08 (-2.17--2) |
| Brunei Darussalam | 22 (9-40) | 22.11 (8.12-39.52) | 45 (19-77) | 14.33 (6.18-24.4) | -1.58 (-1.85--1.31) |
| Bulgaria | 717 (70-1908) | 5.96 (0.65-15.74) | 803 (180-1912) | 6.68 (1.54-15.73) | 0.77 (0.53-1) |
| Burkina Faso | 1549 (884-2205) | 33.36 (19.07-47.26) | 4345 (2299-6469) | 44 (23.51-65.04) | 1.29 (1.11-1.48) |
| Burundi | 1738 (451-3543) | 68.82 (18.11-141.01) | 2132 (499-4395) | 40.52 (9.36-84.17) | -2.4 (-2.61--2.19) |
| Cabo Verde | 143 (73-214) | 65.61 (33.76-97.88) | 342 (133-591) | 78.18 (30.52-135.66) | -0.39 (-0.79-0) |
| Cambodia | 1479 (759-2322) | 29.77 (15.67-46.2) | 2742 (1394-4226) | 21.51 (11.06-33.05) | -1.23 (-1.35--1.11) |
| Cameroon | 680 (142-1346) | 14.02 (2.91-27.72) | 2254 (372-5121) | 17.14 (2.85-38.68) | 0.6 (0.33-0.87) |
| Canada | 3010 (451-6767) | 9.47 (1.44-21.3) | 6192 (1038-13616) | 9.6 (1.65-21.23) | -0.06 (-0.21-0.08) |
| Central African Republic | 1260 (475-2289) | 93.74 (35.27-167.24) | 1946 (818-3330) | 76.02 (32.11-131.32) | -0.81 (-0.88--0.73) |
| Chad | 801 (450-1176) | 27.53 (15.43-40.36) | 2459 (1268-3806) | 40.96 (20.93-63.23) | 1.81 (1.59-2.02) |
| Chile | 2845 (942-5264) | 28.85 (9.48-53.29) | 2146 (436-4745) | 8.92 (1.82-19.75) | -4.61 (-4.83--4.39) |
| China | 1034489 (449013-1696655) | 116.66 (51.12-190.9) | 455090 (90540-1163817) | 22.22 (4.54-56.38) | -6.15 (-6.65--5.65) |
| Colombia | 2526 (594-4918) | 14.03 (3.36-27.41) | 2757 (550-5708) | 5.23 (1.04-10.85) | -3.81 (-4.01--3.61) |
| Comoros | 135 (30-281) | 57.87 (13.01-120.11) | 260 (77-504) | 50.44 (14.89-97.54) | -0.73 (-0.92--0.54) |
| Congo | 1128 (432-2058) | 95.84 (37.12-173.64) | 1728 (663-3068) | 58.45 (22.44-104.43) | -1.98 (-2.18--1.78) |
| Cook Islands | 2 (1-3) | 14 (4.77-26.35) | 2 (1-4) | 8.98 (2.36-18.09) | -1.61 (-1.69--1.53) |
| Costa Rica | 159 (44-299) | 8.97 (2.49-16.86) | 245 (56-496) | 4.77 (1.09-9.67) | -2.44 (-2.73--2.15) |
| Croatia | 893 (158-1944) | 13.65 (2.51-29.49) | 569 (77-1319) | 7.41 (1.05-17.38) | -2.58 (-2.78--2.38) |
| Cuba | 1249 (258-2526) | 12.17 (2.49-24.68) | 1973 (230-4709) | 10.68 (1.27-25.42) | -0.58 (-0.87--0.28) |
| Cyprus | 21 (2-46) | 2.65 (0.27-5.68) | 59 (9-126) | 3.11 (0.5-6.62) | 0.8 (0.6-1) |
| Czechia | 1604 (416-3153) | 12.23 (3.22-24.09) | 2528 (743-4942) | 13.7 (4.14-26.65) | 0.59 (0.38-0.8) |
| Côte d'Ivoire | 758 (210-1537) | 16.39 (4.35-33.12) | 2703 (786-5530) | 22.47 (6.51-45.43) | 1.44 (1.26-1.63) |
| Democratic People's Republic of Korea | 5638 (969-13859) | 31.82 (5.47-78.44) | 11400 (2669-23791) | 34.2 (8.01-71.46) | 0.18 (-0.03-0.38) |
| Democratic Republic of the Congo | 9264 (2858-18032) | 52.79 (16.03-103.33) | 22461 (9593-37742) | 56.54 (24.33-94.63) | 0.01 (-0.29-0.3) |
| Denmark | 1638 (553-2955) | 22.37 (7.55-40.47) | 911 (102-2072) | 8.58 (0.98-19.48) | -3.8 (-4.27--3.33) |
| Djibouti | 199 (98-331) | 118.64 (59.81-190.95) | 664 (333-1210) | 94.86 (47.76-165.28) | -1.05 (-1.21--0.88) |
| Dominica | 6 (1-12) | 9.04 (0.75-18.46) | 8 (1-16) | 8.6 (0.68-18.1) | -0.04 (-0.3-0.21) |
| Dominican Republic | 190 (42-368) | 4.73 (1.01-9.22) | 528 (60-1141) | 5.48 (0.63-11.73) | 0.75 (0.59-0.91) |
| Ecuador | 299 (70-543) | 5.48 (1.28-9.92) | 565 (131-1132) | 3.76 (0.87-7.49) | -1.24 (-1.43--1.05) |
| Egypt | 763 (94-2289) | 2.26 (0.26-6.94) | 691 (103-2692) | 0.92 (0.14-3.65) | -3.34 (-3.73--2.96) |
| El Salvador | 219 (85-383) | 7.2 (2.76-12.7) | 367 (105-710) | 6.24 (1.79-12.07) | -1 (-1.33--0.67) |
| Equatorial Guinea | 177 (67-315) | 80.26 (30.96-140.49) | 158 (47-349) | 30.43 (9.09-65.48) | -4.18 (-4.77--3.59) |
| Eritrea | 1382 (610-2372) | 112.77 (50.52-193.56) | 2808 (1282-4871) | 88.23 (39-154.33) | -1.01 (-1.15--0.87) |
| Estonia | 389 (167-649) | 19.17 (8.29-31.86) | 260 (67-533) | 11.73 (3.06-24.08) | -2.11 (-2.39--1.84) |
| Eswatini | 377 (168-618) | 115.56 (51.24-187.14) | 650 (266-1151) | 100.9 (41.22-178.51) | -0.72 (-1.44-0) |
| Ethiopia | 10108 (5334-16914) | 44.37 (24.19-73.15) | 10512 (5762-16224) | 23.75 (13.11-36.96) | -2.47 (-2.59--2.34) |
| Fiji | 74 (39-111) | 18.52 (9.83-27.94) | 144 (66-233) | 18.33 (8.45-29.37) | 0.33 (0.01-0.65) |
| Finland | 891 (315-1577) | 12.87 (4.55-22.7) | 1007 (311-1938) | 9.29 (2.92-17.71) | -1.05 (-1.29--0.81) |
| France | 22066 (5340-45113) | 30.17 (7.31-61.37) | 18200 (5300-35035) | 15.88 (4.68-30.29) | -2.44 (-2.59--2.3) |
| Gabon | 217 (47-432) | 36.95 (7.9-73.1) | 366 (89-724) | 32.1 (7.81-62.41) | -0.69 (-0.88--0.51) |
| Gambia | 58 (33-85) | 15.25 (8.64-22.01) | 197 (109-289) | 19.51 (10.99-28.28) | 0.73 (0.54-0.91) |
| Georgia | 390 (56-908) | 6.2 (0.94-14.32) | 457 (135-880) | 8.34 (2.55-15.88) | 1.49 (1.3-1.68) |
| Germany | 11936 (1890-25126) | 10.41 (1.67-21.88) | 20315 (4083-42317) | 12.6 (2.53-26.45) | 0.43 (0.17-0.7) |
| Ghana | 902 (281-1779) | 13.16 (4-26.08) | 1590 (326-3070) | 9.22 (1.91-17.74) | -1.18 (-1.27--1.09) |
| Greece | 72 (29-250) | 0.53 (0.19-1.85) | 129 (28-433) | 0.62 (0.14-2.19) | -0.25 (-0.67-0.18) |
| Greenland | 19 (4-40) | 46.6 (10.53-99.32) | 22 (4-53) | 28.84 (5.2-68.77) | -1.97 (-2.18--1.76) |
| Grenada | 16 (4-31) | 25.01 (6.5-46.84) | 26 (7-49) | 21.95 (5.59-41.47) | -0.15 (-0.28--0.03) |
| Guam | 7 (2-13) | 8.55 (2.27-16.34) | 16 (4-32) | 8.58 (2.16-16.95) | 0.39 (-0.05-0.83) |
| Guatemala | 328 (123-560) | 8.41 (3.1-14.4) | 970 (299-1891) | 8.39 (2.55-16.44) | -0.18 (-0.58-0.23) |
| Guinea | 192 (41-381) | 5.58 (1.23-11.05) | 458 (131-910) | 7.81 (2.24-15.42) | 1.49 (1.32-1.66) |
| Guinea-Bissau | 157 (66-271) | 35.23 (14.71-60.69) | 320 (127-571) | 39.53 (15.48-70.14) | 0.98 (0.76-1.2) |
| Guyana | 53 (21-89) | 12.92 (5.13-21.97) | 80 (31-144) | 11.62 (4.37-20.9) | -0.12 (-0.22--0.02) |
| Haiti | 666 (186-1383) | 18.78 (5.18-38.98) | 1383 (457-2863) | 18.04 (5.85-37.02) | -0.01 (-0.12-0.11) |
| Honduras | 112 (34-217) | 5.12 (1.52-9.97) | 394 (103-813) | 6.41 (1.64-13.07) | 1.04 (0.89-1.18) |
| Hungary | 2340 (405-5002) | 17.18 (3.01-36.61) | 2126 (454-4801) | 12.87 (2.75-28.93) | -1.82 (-2.34--1.29) |
| Iceland | 63 (26-105) | 23.25 (9.68-38.86) | 74 (20-146) | 14.5 (3.96-28.48) | -1.91 (-2.12--1.7) |
| India | 188118 (110965-268078) | 36.96 (21.78-52.8) | 352566 (194826-526531) | 29.09 (16.12-43.5) | -1.09 (-1.24--0.95) |
| Indonesia | 21069 (11079-30829) | 19.13 (10.09-27.92) | 26803 (10419-47327) | 11.51 (4.47-20.19) | -1.8 (-1.88--1.72) |
| Iran (Islamic Republic of) | 2933 (447-6127) | 10.66 (1.6-22.04) | 2198 (422-5417) | 2.95 (0.56-7.13) | -5.05 (-5.6--4.5) |
| Iraq | 215 (21-605) | 2.57 (0.23-7.18) | 974 (214-2257) | 3.72 (0.78-8.76) | 0.54 (0.07-1.01) |
| Ireland | 1375 (568-2248) | 34.35 (14.26-56.01) | 1128 (184-2576) | 15.51 (2.51-35.65) | -3.18 (-3.42--2.94) |
| Israel | 36 (8-111) | 0.78 (0.18-2.44) | 70 (15-252) | 0.63 (0.13-2.33) | -0.5 (-0.69--0.31) |
| Italy | 2737 (449-7270) | 3.29 (0.55-8.64) | 3316 (513-7171) | 2.66 (0.4-5.86) | -0.63 (-0.8--0.45) |
| Jamaica | 152 (27-309) | 8.7 (1.51-17.78) | 237 (44-497) | 8.02 (1.47-16.87) | -0.17 (-0.5-0.16) |
| Japan | 32132 (11241-59480) | 18.57 (6.49-34.33) | 35053 (11794-69611) | 12.82 (4.59-24.27) | -1.07 (-1.3--0.84) |
| Jordan | 57 (8-130) | 3.93 (0.53-8.95) | 245 (46-529) | 3.33 (0.58-7.3) | -1.04 (-1.27--0.81) |
| Kazakhstan | 16397 (7506-24524) | 126.35 (57.98-189.26) | 3410 (729-8386) | 19.34 (4.27-47) | -7.26 (-7.6--6.92) |
| Kenya | 5482 (2453-9303) | 61.46 (27.47-104.07) | 17127 (7121-29711) | 69.9 (28.81-120.49) | 0.78 (0.57-1) |
| Kiribati | 20 (7-36) | 46.7 (16.3-85.61) | 32 (10-62) | 40.54 (12.84-76.88) | -0.72 (-0.9--0.55) |
| Kuwait | 20 (2-56) | 2.73 (0.25-7.97) | 60 (9-173) | 2.07 (0.26-5.98) | -0.85 (-1.12--0.58) |
| Kyrgyzstan | 1469 (625-2353) | 47.13 (20-75.44) | 753 (253-1444) | 16.15 (5.4-30.7) | -4.49 (-4.75--4.23) |
| Lao People's Democratic Republic | 720 (332-1171) | 31.28 (14.37-50.64) | 457 (113-960) | 9.5 (2.34-20.09) | -4.72 (-4.95--4.48) |
| Latvia | 566 (202-979) | 16 (5.74-27.57) | 591 (213-1042) | 17.8 (6.45-31.7) | 0.11 (-0.17-0.4) |
| Lebanon | 5 (3-12) | 0.21 (0.11-0.52) | 26 (5-97) | 0.49 (0.1-1.83) | 3.78 (3.34-4.22) |
| Lesotho | 1090 (550-1667) | 104.71 (52.92-159.18) | 1825 (889-2906) | 131.91 (65.69-206.54) | 1.13 (0.85-1.42) |
| Liberia | 257 (104-445) | 22.2 (8.97-38.53) | 718 (304-1223) | 31.54 (13.26-53.85) | 1.67 (1.34-2) |
| Libya | 40 (3-106) | 1.99 (0.17-5.29) | 175 (17-434) | 3.09 (0.32-7.75) | 0.95 (0.46-1.44) |
| Lithuania | 707 (248-1276) | 15.81 (5.6-28.43) | 758 (203-1507) | 15.8 (4.31-31.3) | -0.03 (-0.32-0.27) |
| Luxembourg | 91 (21-182) | 17.62 (4.08-34.98) | 98 (24-204) | 10.39 (2.56-21.62) | -1.86 (-1.91--1.8) |
| Madagascar | 4429 (1806-7602) | 77.64 (31.16-134.53) | 8772 (3824-14990) | 67.76 (28.98-116.97) | -0.71 (-0.94--0.49) |
| Malawi | 6420 (2924-10553) | 148.05 (66.05-243.49) | 11645 (4274-20657) | 143.49 (51.94-252.98) | -0.21 (-0.46-0.04) |
| Malaysia | 1599 (726-2556) | 16.56 (7.51-26.57) | 2372 (582-4919) | 8.65 (2.17-17.78) | -2.86 (-3.2--2.52) |
| Maldives | 26 (9-49) | 24.81 (8.54-46.81) | 26 (9-47) | 7.95 (2.78-14.25) | -4.64 (-4.96--4.32) |
| Mali | 891 (456-1300) | 19.76 (10.15-28.76) | 1620 (753-2707) | 17.26 (8.08-28.32) | -0.5 (-0.58--0.41) |
| Malta | 35 (5-80) | 8.22 (1.28-18.73) | 37 (6-96) | 4.6 (0.75-11.88) | -1.54 (-1.94--1.13) |
| Marshall Islands | 3 (1-6) | 18.83 (7.98-33.7) | 6 (3-11) | 15.96 (6.82-28.92) | -0.56 (-0.64--0.48) |
| Mauritania | 401 (231-593) | 38.36 (22.01-57.02) | 734 (375-1156) | 34.2 (17.28-53.34) | 0.08 (-0.15-0.31) |
| Mauritius | 192 (112-267) | 24.88 (14.39-34.53) | 264 (121-420) | 14.77 (6.86-23.47) | -1.97 (-2.11--1.83) |
| Mexico | 2890 (797-5319) | 6.46 (1.76-11.94) | 5081 (1305-9571) | 4.23 (1.08-7.96) | -1.55 (-1.83--1.27) |
| Micronesia (Federated States of) | 11 (5-19) | 21.86 (9.52-37.13) | 14 (6-26) | 18.36 (7.59-32.61) | -0.77 (-0.83--0.72) |
| Monaco | 2 (0-7) | 3.83 (0.57-11.66) | 2 (0-6) | 2.35 (0.49-7.89) | -1.24 (-1.48--1) |
| Mongolia | 2127 (1256-3038) | 206.81 (120.79-296.54) | 3606 (1966-5529) | 161.89 (90.16-242.63) | -1.34 (-1.6--1.07) |
| Montenegro | 15 (2-52) | 2.26 (0.26-7.94) | 11 (2-40) | 1.2 (0.27-4.29) | -4.17 (-5.02--3.32) |
| Morocco | 783 (170-1638) | 5.37 (1.14-11.19) | 582 (65-1462) | 1.81 (0.21-4.47) | -4.58 (-5.21--3.94) |
| Mozambique | 3316 (1826-4912) | 52.24 (29.3-77.73) | 7233 (3698-11437) | 61.54 (31.44-96.65) | 0.89 (0.75-1.04) |
| Myanmar | 7571 (3785-12112) | 29.62 (15.07-46.92) | 6988 (3098-11699) | 14.14 (6.27-23.4) | -2.83 (-2.94--2.73) |
| Namibia | 160 (85-238) | 21.15 (11.17-31.46) | 321 (159-495) | 21.12 (10.64-32.51) | -0.35 (-0.71-0.01) |
| Nauru | 1 (0-1) | 16.28 (5.23-31.58) | 1 (0-2) | 15.28 (5.46-28.79) | -0.47 (-1.07-0.14) |
| Nepal | 4350 (2190-6721) | 40.81 (20.77-63.09) | 6356 (2687-10980) | 27.27 (11.59-47.15) | -1.34 (-1.73--0.95) |
| Netherlands | 2769 (676-5505) | 14.56 (3.57-28.75) | 6168 (1105-12853) | 19.45 (3.48-40.36) | 0.98 (0.65-1.31) |
| New Zealand | 448 (65-983) | 11.55 (1.69-25.17) | 707 (107-1522) | 9.49 (1.51-20.37) | -0.63 (-0.7--0.56) |
| Nicaragua | 118 (58-181) | 7.42 (3.63-11.36) | 364 (184-563) | 8.18 (4.15-12.7) | -0.22 (-0.5-0.06) |
| Niger | 905 (501-1325) | 29.33 (16.3-42.77) | 2385 (1108-3876) | 28.64 (13.25-45.7) | 0.17 (0-0.34) |
| Nigeria | 2128 (757-4695) | 4.57 (1.62-10.01) | 3759 (1362-8207) | 4.09 (1.44-8.66) | -0.4 (-0.53--0.27) |
| Niue | 0 (0-1) | 12.95 (4.78-23.79) | 0 (0-0) | 10.63 (3.77-20.2) | -1.02 (-1.2--0.84) |
| North Macedonia | 50 (5-158) | 2.54 (0.27-7.94) | 58 (7-196) | 1.87 (0.21-6.13) | -1.71 (-2.04--1.38) |
| Northern Mariana Islands | 1 (0-3) | 5.28 (1.35-11.21) | 6 (2-11) | 10.08 (2.75-19.37) | 3.33 (2.88-3.79) |
| Norway | 585 (185-1076) | 9.6 (3.04-17.63) | 602 (140-1150) | 6.74 (1.58-12.86) | -1.25 (-1.33--1.17) |
| Oman | 32 (3-75) | 4.33 (0.37-10.04) | 58 (7-137) | 2.98 (0.32-6.63) | -1.21 (-1.35--1.07) |
| Pakistan | 29923 (14638-46744) | 49.36 (24.12-77.32) | 69008 (34084-108709) | 54.66 (26.81-87.28) | 0.3 (0.06-0.54) |
| Palau | 1 (0-2) | 11.04 (4.02-20.47) | 2 (1-5) | 10.5 (3.45-19.48) | -0.28 (-0.38--0.17) |
| Palestine | 61 (22-111) | 6.76 (2.34-12.15) | 80 (17-178) | 3.16 (0.66-6.91) | -3.29 (-3.64--2.94) |
| Panama | 116 (41-207) | 7.62 (2.65-13.65) | 280 (90-542) | 6.74 (2.16-13.06) | 0.01 (-0.29-0.31) |
| Papua New Guinea | 234 (96-435) | 11.31 (4.69-21.21) | 568 (230-1051) | 10.55 (4.14-19.84) | -0.19 (-0.27--0.11) |
| Paraguay | 205 (46-397) | 9.07 (2.02-17.59) | 696 (158-1407) | 12.23 (2.79-24.54) | 0.87 (0.56-1.18) |
| Peru | 1083 (413-1869) | 8.75 (3.37-15.03) | 1391 (359-2867) | 4.3 (1.1-8.9) | -2.61 (-2.76--2.46) |
| Philippines | 2274 (808-4075) | 6.67 (2.34-11.9) | 4362 (1350-8568) | 5.04 (1.55-9.89) | -1.21 (-1.35--1.07) |
| Poland | 7454 (2783-13150) | 17.31 (6.5-30.45) | 6758 (1853-14506) | 10.66 (2.91-22.87) | -1.96 (-2.16--1.77) |
| Portugal | 1259 (125-3401) | 9.6 (1.01-25.79) | 1026 (102-2939) | 5.36 (0.53-15.49) | -1.24 (-1.55--0.93) |
| Puerto Rico | 1041 (376-1840) | 28.86 (10.43-50.9) | 518 (135-1044) | 8.01 (2.11-16.29) | -5.13 (-5.5--4.76) |
| Qatar | 3 (1-9) | 2.27 (0.41-6.72) | 10 (2-35) | 1.06 (0.3-3.43) | -3.87 (-4.29--3.46) |
| Republic of Korea | 5584 (1268-12940) | 16.76 (4.04-38.74) | 4245 (665-11281) | 4.69 (0.76-12.36) | -4.98 (-5.38--4.58) |
| Republic of Moldova | 421 (84-945) | 9.13 (1.88-20.43) | 497 (146-968) | 8.78 (2.6-17.13) | -0.19 (-0.44-0.05) |
| Romania | 1301 (239-3163) | 4.64 (0.88-11.19) | 1642 (178-5070) | 5.26 (0.58-16.21) | -0.19 (-0.61-0.22) |
| Russian Federation | 47593 (19877-79564) | 25.65 (10.76-42.84) | 31846 (10285-59723) | 13.96 (4.67-26.06) | -2.57 (-2.88--2.27) |
| Rwanda | 1857 (349-3619) | 58.26 (10.88-112.48) | 1857 (360-3762) | 28.04 (5.55-56.64) | -3.61 (-4.02--3.21) |
| Saint Kitts and Nevis | 13 (7-19) | 38.36 (20.44-54.78) | 20 (10-31) | 27.43 (13.21-42.66) | -1.39 (-1.77--1.02) |
| Saint Lucia | 14 (3-25) | 16.14 (3.83-29.41) | 38 (11-73) | 17.33 (5.07-33.11) | 0.42 (0.23-0.61) |
| Saint Vincent and the Grenadines | 8 (3-15) | 11.78 (4.42-20.69) | 11 (2-20) | 7.67 (1.74-14.7) | -1.82 (-2.29--1.35) |
| Samoa | 11 (5-19) | 12.44 (5.54-20.72) | 13 (6-23) | 8.8 (3.66-15.21) | -1.48 (-1.59--1.38) |
| San Marino | 1 (0-3) | 3.71 (0.58-8.46) | 2 (0-5) | 3.33 (0.51-8.18) | -0.57 (-1.03--0.12) |
| Sao Tome and Principe | 9 (3-16) | 12.83 (3.99-24.48) | 17 (4-34) | 14.87 (3.8-29.96) | 0.72 (0.49-0.94) |
| Saudi Arabia | 244 (24-588) | 3.79 (0.35-9.06) | 1115 (203-2450) | 4.84 (0.8-10.56) | 0.7 (0.27-1.13) |
| Senegal | 1010 (518-1513) | 29.55 (15.3-44.14) | 2632 (1226-4201) | 33.38 (15.54-52.77) | 1.01 (0.64-1.38) |
| Serbia | 830 (98-1976) | 6.95 (0.87-16.36) | 703 (64-1709) | 4.94 (0.48-12.16) | -1.86 (-2.17--1.54) |
| Seychelles | 18 (8-30) | 33.08 (14.18-54.22) | 31 (11-57) | 25.66 (8.91-47.05) | -1.06 (-1.19--0.94) |
| Sierra Leone | 455 (210-718) | 23.08 (10.65-36.39) | 1118 (469-1887) | 29.12 (12.24-48.92) | 1.34 (1.11-1.57) |
| Singapore | 332 (59-734) | 14.81 (2.64-32.62) | 186 (18-480) | 2.36 (0.23-6.05) | -6.39 (-6.71--6.07) |
| Slovakia | 1149 (410-2042) | 19.89 (7.14-35.29) | 1227 (359-2567) | 14.13 (4.18-29.68) | -1.58 (-1.84--1.32) |
| Slovenia | 282 (38-633) | 11.58 (1.56-25.83) | 249 (31-563) | 6.59 (0.82-15.05) | -2.45 (-2.72--2.18) |
| Solomon Islands | 42 (19-73) | 25.86 (11.64-45.32) | 85 (33-158) | 22.5 (9.1-40.91) | -0.62 (-0.78--0.47) |
| Somalia | 4250 (1832-7135) | 139.02 (62.24-228.6) | 8595 (4257-14299) | 112.02 (56.28-186.24) | -0.78 (-0.87--0.68) |
| South Africa | 23760 (12162-36186) | 103.5 (52.45-158.48) | 32146 (16727-49188) | 68.32 (35.56-103.83) | -2.18 (-2.84--1.51) |
| South Sudan | 1669 (549-3219) | 65.26 (21.57-125.31) | 2022 (648-4146) | 46.57 (14.67-94.66) | -1.42 (-1.52--1.33) |
| Spain | 1581 (226-5320) | 3.23 (0.46-10.8) | 3132 (329-7410) | 3.79 (0.39-9.08) | 0.78 (0.63-0.93) |
| Sri Lanka | 3682 (1804-5626) | 31.62 (15.48-48.52) | 6837 (3138-11665) | 26.11 (12.1-44.25) | -0.19 (-0.69-0.31) |
| Sudan | 2075 (494-4348) | 20.86 (5.01-43.47) | 3648 (777-8135) | 18.13 (3.76-40.43) | -0.67 (-0.78--0.55) |
| Suriname | 17 (5-32) | 6.1 (1.79-11.88) | 36 (11-72) | 5.73 (1.66-11.41) | -0.28 (-0.55-0) |
| Sweden | 1386 (443-2607) | 10.02 (3.15-19) | 1447 (344-2854) | 7.6 (1.72-15.2) | -1.21 (-1.33--1.09) |
| Switzerland | 1414 (238-2971) | 14.85 (2.48-31.5) | 1462 (242-3077) | 9.28 (1.55-19.59) | -1.62 (-1.69--1.56) |
| Syrian Arab Republic | 86 (7-224) | 1.49 (0.12-3.75) | 319 (43-727) | 2.41 (0.33-5.42) | 0.93 (0.63-1.24) |
| Taiwan (Province of China) | 2870 (441-6107) | 16.78 (2.61-35.69) | 5137 (516-14157) | 13.8 (1.37-37.78) | -0.13 (-0.38-0.12) |
| Tajikistan | 1792 (670-3064) | 61.21 (22.65-104.73) | 1704 (534-3913) | 31.05 (9.75-72.92) | -2.69 (-3.46--1.91) |
| Thailand | 7058 (2223-13461) | 17.83 (5.52-34.28) | 9847 (1967-21162) | 9.45 (1.91-20.19) | -2.83 (-3.12--2.54) |
| Timor-Leste | 73 (39-112) | 21.68 (11.59-32.84) | 157 (78-257) | 18.61 (9.36-30.4) | -0.63 (-0.89--0.36) |
| Togo | 433 (243-624) | 31.89 (18.06-46.07) | 1727 (861-2770) | 42.14 (20.85-66.91) | 1.35 (1.19-1.52) |
| Tokelau | 0 (0-0) | 13.34 (5.66-22.69) | 0 (0-0) | 9.2 (3.48-16.99) | -1.43 (-1.49--1.37) |
| Tonga | 7 (3-12) | 11.74 (5.13-20.28) | 9 (4-16) | 10.88 (4.41-19.69) | -0.5 (-0.81--0.19) |
| Trinidad and Tobago | 126 (58-197) | 14.53 (6.67-22.68) | 158 (63-285) | 8.54 (3.44-15.39) | -2.6 (-2.98--2.22) |
| Tunisia | 119 (13-280) | 2.29 (0.26-5.38) | 132 (14-431) | 1.03 (0.11-3.34) | -3.42 (-3.7--3.14) |
| Turkey | 514 (79-2075) | 1.22 (0.2-4.91) | 380 (119-1300) | 0.41 (0.13-1.41) | -4.37 (-4.7--4.05) |
| Turkmenistan | 2432 (623-5070) | 120.38 (30.58-250.42) | 683 (76-2136) | 15.97 (1.78-49.84) | -7.95 (-8.51--7.4) |
| Tuvalu | 1 (1-2) | 19.41 (8.91-31.12) | 1 (1-3) | 13.53 (5.88-24.24) | -1.33 (-1.42--1.24) |
| Uganda | 2729 (657-5124) | 39.31 (9.39-74.45) | 7806 (1879-14662) | 49.51 (11.83-91.57) | 0.76 (0.58-0.95) |
| Ukraine | 12978 (4728-22741) | 18.4 (6.76-32.07) | 9927 (3300-18804) | 14.29 (4.76-26.95) | -2.2 (-2.72--1.68) |
| United Arab Emirates | 16 (2-65) | 1.9 (0.33-7.23) | 1126 (106-3041) | 15.4 (1.41-41.04) | 8.41 (7.55-9.28) |
| United Kingdom | 28216 (12280-46743) | 32.99 (14.47-54.63) | 33258 (12486-58100) | 28.6 (10.82-50.02) | -0.76 (-0.87--0.66) |
| United Republic of Tanzania | 11822 (5048-20581) | 99.79 (42.97-172.47) | 14710 (4581-28419) | 54.42 (16.75-104.82) | -2.51 (-2.66--2.35) |
| United States of America | 36509 (9035-76374) | 12.31 (3.05-25.77) | 60195 (15745-123468) | 11.47 (3.05-23.55) | -0.08 (-0.16--0.01) |
| United States Virgin Islands | 10 (3-20) | 11.28 (2.99-21.28) | 19 (3-37) | 10.44 (1.72-20.99) | 0.18 (-0.2-0.56) |
| Uruguay | 1554 (506-2850) | 40.89 (13.44-74.97) | 1005 (234-1977) | 20.04 (4.69-39.55) | -2.58 (-2.72--2.44) |
| Uzbekistan | 8275 (2750-15246) | 69.86 (23.12-128.69) | 2990 (360-8835) | 12.65 (1.58-37.27) | -7.43 (-8.04--6.81) |
| Vanuatu | 7 (2-15) | 10.35 (2.52-21.59) | 22 (7-46) | 11.83 (3.8-24.35) | 0.32 (0.19-0.44) |
| Venezuela (Bolivarian Republic of) | 824 (202-1573) | 8.25 (2.03-15.83) | 1947 (432-4026) | 6.53 (1.45-13.51) | -1.08 (-1.29--0.87) |
| Viet Nam | 6230 (2930-10278) | 15.05 (7.06-24.71) | 11279 (2645-24301) | 10.93 (2.61-23.15) | -0.96 (-1.05--0.86) |
| Yemen | 1532 (449-2712) | 28.42 (8.44-50.18) | 3881 (1084-6942) | 26.59 (7.25-47.45) | -0.58 (-0.71--0.44) |
| Zambia | 5311 (2824-7687) | 166.1 (89.02-240.26) | 10140 (5396-16265) | 132.3 (71.48-208.77) | -1.24 (-1.53--0.95) |
| Zimbabwe | 5827 (3377-8107) | 133.55 (78.14-185.27) | 10851 (5993-15566) | 140.27 (78.05-201.96) | 0.16 (-0.11-0.42) |

**Table S3.** The number of deaths cases and the age-standardized deaths rate of esophageal cancer attributable to diet low in vegetables in 1990 and 2019, and its trends from 1990 to 2019 globally.

|  | Number of deaths cases (95% UI) in 1990 | The age-standardized deaths rate/100000 (95% UI) in 1990 | Number of deaths cases (95% UI) in 2019 | The age-standardized deaths rate/100000 (95% UI) in 2019 | EAPC (95% CI) |
| --- | --- | --- | --- | --- | --- |
| Global | 21591 (2291-44769) | 0.55 (0.06-1.15) | 17176 (2549-33958) | 0.21 (0.03-0.42) | -3.6 (-3.83--3.37) |
| **Sex** |  |  |  |  |  |
| Female | 7252 (797-14894) | 0.35 (0.04-0.71) | 5603 (868-10917) | 0.13 (0.02-0.25) | -3.71 (-3.97--3.45) |
| Male | 14339 (1421-29787) | 0.8 (0.08-1.67) | 11573 (1709-23211) | 0.31 (0.04-0.62) | -3.55 (-3.76--3.34) |
| **Age** |  |  |  |  |  |
| <24 years | 0 (0-0) | 0 (0-0) | 0 (0-0) | 0 (0-0) | 0 (0-0) |
| 25-29 years | 51 (7-100) | 0.01 (0-0.02) | 45 (7-89) | 0.01 (0-0.01) | -1.97 (-2.14--1.81) |
| 30-34 years | 79 (12-158) | 0.02 (0-0.04) | 90 (14-175) | 0.01 (0-0.03) | -1.23 (-1.36--1.09) |
| 35-39 years | 207 (28-423) | 0.06 (0.01-0.12) | 201 (32-388) | 0.04 (0.01-0.07) | -1.58 (-1.75--1.4) |
| 40-44 years | 606 (66-1254) | 0.21 (0.02-0.44) | 439 (72-858) | 0.09 (0.01-0.17) | -3.56 (-3.95--3.17) |
| 45-49 years | 1002 (115-2043) | 0.43 (0.05-0.88) | 836 (131-1632) | 0.18 (0.03-0.34) | -3.53 (-3.78--3.27) |
| 50-54 years | 1803 (198-3652) | 0.85 (0.09-1.72) | 1434 (227-2814) | 0.33 (0.05-0.64) | -3.46 (-3.72--3.2) |
| 55-59 years | 2681 (266-5559) | 1.45 (0.14-3) | 1970 (295-3908) | 0.53 (0.08-1.05) | -3.51 (-3.88--3.13) |
| 60-64 years | 3300 (334-6848) | 2.05 (0.21-4.26) | 2240 (343-4370) | 0.72 (0.11-1.4) | -3.88 (-4.21--3.54) |
| 65-69 years | 3541 (353-7453) | 2.87 (0.29-6.04) | 2414 (376-4735) | 0.93 (0.15-1.83) | -4.04 (-4.35--3.72) |
| 70-74 years | 3335 (333-6952) | 3.95 (0.39-8.23) | 2317 (360-4480) | 1.24 (0.19-2.39) | -4.25 (-4.6--3.89) |
| 75-79 years | 2566 (260-5253) | 4.19 (0.42-8.57) | 1953 (292-3892) | 1.54 (0.23-3.06) | -4.07 (-4.34--3.81) |
| 80-84 years | 1593 (162-3265) | 4.52 (0.46-9.27) | 1611 (223-3271) | 1.91 (0.26-3.88) | -3.43 (-3.63--3.22) |
| 85-89 years | 628 (71-1323) | 4.17 (0.47-8.78) | 1080 (145-2264) | 2.48 (0.33-5.21) | -1.92 (-2.19--1.66) |
| 90-94 years | 164 (18-340) | 3.72 (0.4-7.71) | 417 (48-876) | 2.48 (0.28-5.2) | -1.44 (-1.55--1.32) |
| 95+ years | 36 (4-75) | 3.45 (0.35-7.31) | 128 (14-272) | 2.67 (0.28-5.7) | -0.68 (-0.77--0.58) |
| **SDI regions** |  |  |  |  |  |
| High SDI | 2596 (148-5500) | 0.25 (0.01-0.53) | 3844 (258-8108) | 0.2 (0.01-0.43) | -0.65 (-0.7--0.6) |
| High-middle SDI | 4972 (318-10650) | 0.47 (0.03-1.01) | 2166 (368-4396) | 0.11 (0.02-0.22) | -5.5 (-5.95--5.06) |
| Middle SDI | 9725 (731-20600) | 1 (0.08-2.13) | 3853 (639-7531) | 0.16 (0.03-0.32) | -6.82 (-7.09--6.54) |
| Low-middle SDI | 2733 (438-5408) | 0.48 (0.08-0.94) | 4385 (612-8543) | 0.33 (0.05-0.64) | -1.47 (-1.55--1.38) |
| Low SDI | 1559 (271-2884) | 0.68 (0.12-1.27) | 2918 (542-5635) | 0.58 (0.11-1.11) | -0.67 (-0.74--0.6) |
| **GBD regions** |  |  |  |  |  |
| Africa | 1824 (331-3482) | 0.66 (0.12-1.26) | 3345 (624-6528) | 0.56 (0.1-1.08) | -0.82 (-0.98--0.66) |
| African Region | 1718 (312-3271) | 0.79 (0.14-1.52) | 3156 (600-6135) | 0.68 (0.13-1.31) | -0.8 (-0.97--0.62) |
| America | 1764 (189-3575) | 0.29 (0.03-0.59) | 3210 (283-6514) | 0.25 (0.02-0.51) | -0.48 (-0.53--0.44) |
| Andean Latin America | 41 (6-76) | 0.21 (0.03-0.4) | 83 (13-165) | 0.15 (0.02-0.3) | -1.08 (-1.16--1.01) |
| Asia | 15754 (1354-33097) | 0.82 (0.07-1.71) | 7874 (1359-15314) | 0.17 (0.03-0.33) | -5.93 (-6.26--5.6) |
| Australasia | 69 (3-145) | 0.29 (0.01-0.62) | 126 (5-269) | 0.25 (0.01-0.53) | -0.67 (-0.73--0.61) |
| Caribbean | 95 (15-181) | 0.37 (0.06-0.71) | 133 (18-270) | 0.26 (0.04-0.52) | -1.46 (-1.69--1.24) |
| Central Asia | 257 (24-577) | 0.58 (0.05-1.29) | 70 (15-145) | 0.11 (0.02-0.24) | -7.12 (-7.75--6.49) |
| Central Europe | 189 (10-418) | 0.13 (0.01-0.29) | 158 (13-359) | 0.08 (0.01-0.17) | -2.23 (-2.38--2.07) |
| Central Latin America | 178 (22-349) | 0.23 (0.03-0.46) | 330 (33-664) | 0.14 (0.01-0.29) | -1.91 (-2.05--1.77) |
| Central Sub-Saharan Africa | 285 (49-554) | 1.31 (0.23-2.59) | 491 (82-998) | 0.98 (0.16-2.01) | -1.24 (-1.34--1.15) |
| Commonwealth High Income | 611 (40-1276) | 0.4 (0.03-0.84) | 927 (54-1924) | 0.35 (0.02-0.72) | -0.61 (-0.68--0.55) |
| Commonwealth Low Income | 881 (157-1665) | 1.03 (0.19-1.95) | 1665 (299-3321) | 0.78 (0.14-1.54) | -1.01 (-1.12--0.91) |
| Commonwealth Middle Income | 2599 (398-5071) | 0.45 (0.07-0.88) | 4885 (562-9536) | 0.34 (0.04-0.66) | -1.29 (-1.5--1.08) |
| East Asia | 11769 (596-25367) | 1.42 (0.07-3.09) | 1293 (508-2856) | 0.07 (0.03-0.16) | -11 (-11.31--10.68) |
| East Asia & Pacific - WB | 12857 (799-27643) | 0.99 (0.06-2.13) | 2888 (711-5953) | 0.1 (0.02-0.2) | -8.68 (-9.03--8.33) |
| Eastern Europe | 536 (28-1185) | 0.19 (0.01-0.42) | 478 (26-1074) | 0.14 (0.01-0.31) | -1.25 (-1.7--0.78) |
| Eastern Mediterranean Region | 707 (117-1371) | 0.4 (0.07-0.79) | 1318 (220-2546) | 0.32 (0.05-0.61) | -1 (-1.09--0.91) |
| Eastern Sub-Saharan Africa | 926 (170-1710) | 1.28 (0.23-2.39) | 1716 (328-3380) | 1.1 (0.21-2.17) | -0.63 (-0.71--0.55) |
| Europe | 2216 (129-4729) | 0.22 (0.01-0.46) | 2718 (160-5744) | 0.17 (0.01-0.37) | -0.84 (-0.89--0.8) |
| Europe & Central Asia - WB | 2422 (141-5192) | 0.23 (0.01-0.49) | 2738 (167-5775) | 0.17 (0.01-0.36) | -1.2 (-1.26--1.13) |
| European Region | 2423 (141-5196) | 0.23 (0.01-0.49) | 2739 (168-5777) | 0.17 (0.01-0.35) | -1.21 (-1.27--1.14) |
| High-income Asia Pacific | 320 (22-730) | 0.16 (0.01-0.37) | 298 (34-764) | 0.06 (0.01-0.16) | -2.93 (-3.3--2.56) |
| High-income North America | 605 (31-1322) | 0.17 (0.01-0.38) | 1359 (103-2791) | 0.21 (0.02-0.44) | 1.08 (0.89-1.27) |
| Latin America & Caribbean - WB | 1186 (165-2321) | 0.46 (0.06-0.91) | 1870 (182-3728) | 0.28 (0.03-0.56) | -1.86 (-1.95--1.77) |
| Middle East & North Africa - WB | 142 (11-309) | 0.12 (0.01-0.27) | 202 (26-450) | 0.07 (0.01-0.15) | -2.41 (-2.69--2.12) |
| North Africa and Middle East | 232 (29-486) | 0.14 (0.02-0.3) | 352 (51-751) | 0.09 (0.01-0.19) | -2.08 (-2.27--1.89) |
| North America | 605 (31-1321) | 0.17 (0.01-0.38) | 1359 (103-2790) | 0.21 (0.02-0.44) | 1.08 (0.89-1.27) |
| Oceania | 6 (1-13) | 0.23 (0.04-0.46) | 14 (2-29) | 0.22 (0.04-0.46) | -0.09 (-0.12--0.06) |
| Region of the Americas | 1764 (189-3575) | 0.29 (0.03-0.59) | 3210 (283-6514) | 0.25 (0.02-0.51) | -0.48 (-0.53--0.44) |
| South-East Asia Region | 2615 (416-5087) | 0.4 (0.06-0.77) | 4794 (545-9530) | 0.29 (0.03-0.57) | -1.28 (-1.39--1.17) |
| South Asia | 2470 (402-4786) | 0.46 (0.08-0.89) | 4619 (573-9116) | 0.34 (0.04-0.66) | -1.29 (-1.42--1.17) |
| South Asia - WB | 2582 (420-4989) | 0.47 (0.08-0.9) | 4809 (616-9386) | 0.34 (0.04-0.67) | -1.27 (-1.4--1.14) |
| Southeast Asia | 721 (115-1370) | 0.29 (0.05-0.55) | 1222 (128-2450) | 0.21 (0.02-0.42) | -1.32 (-1.4--1.24) |
| Southern Latin America | 281 (24-567) | 0.63 (0.05-1.26) | 263 (14-556) | 0.31 (0.02-0.66) | -2.71 (-2.84--2.57) |
| Southern Sub-Saharan Africa | 363 (63-716) | 1.34 (0.23-2.66) | 578 (99-1119) | 1.07 (0.18-2.08) | -1.38 (-1.92--0.84) |
| Sub-Saharan Africa - WB | 1791 (324-3414) | 0.83 (0.15-1.59) | 3299 (616-6424) | 0.72 (0.13-1.39) | -0.7 (-0.87--0.53) |
| Tropical Latin America | 595 (94-1151) | 0.67 (0.11-1.29) | 1065 (97-2140) | 0.44 (0.04-0.88) | -1.51 (-1.58--1.45) |
| Western Europe | 1468 (88-3065) | 0.26 (0.02-0.54) | 2072 (115-4384) | 0.23 (0.01-0.48) | -0.5 (-0.58--0.42) |
| Western Pacific Region | 12294 (663-26515) | 1.09 (0.06-2.36) | 1918 (606-4201) | 0.07 (0.02-0.16) | -9.95 (-10.29--9.61) |
| Western Sub-Saharan Africa | 185 (29-354) | 0.22 (0.03-0.42) | 455 (75-904) | 0.26 (0.04-0.51) | 0.81 (0.71-0.92) |
| World Bank High Income | 2797 (172-5924) | 0.22 (0.01-0.46) | 4212 (283-8913) | 0.18 (0.01-0.39) | -0.52 (-0.58--0.47) |
| World Bank Low Income | 1334 (240-2502) | 0.92 (0.17-1.73) | 2467 (446-4705) | 0.81 (0.15-1.56) | -0.53 (-0.6--0.47) |
| World Bank Lower Middle Income | 3564 (577-6907) | 0.36 (0.06-0.69) | 6437 (812-12784) | 0.28 (0.04-0.55) | -1.03 (-1.13--0.93) |
| World Bank Upper Middle Income | 13891 (901-29615) | 0.95 (0.06-2.03) | 4051 (821-7981) | 0.12 (0.02-0.25) | -7.62 (-7.95--7.29) |
| **Countries** |  |  |  |  |  |
| Afghanistan | 59 (8-126) | 0.84 (0.11-1.77) | 92 (13-197) | 0.77 (0.11-1.63) | -0.39 (-0.54--0.25) |
| Albania | 2 (0-5) | 0.12 (0.01-0.27) | 0 (0-1) | 0.01 (0-0.02) | -11.24 (-12.03--10.45) |
| Algeria | 12 (1-25) | 0.11 (0.01-0.23) | 18 (1-43) | 0.06 (0-0.14) | -2.51 (-2.69--2.32) |
| American Samoa | 0 (0-0) | 0.13 (0.02-0.26) | 0 (0-0) | 0.15 (0.02-0.28) | 0.44 (0.28-0.61) |
| Andorra | 0 (0-0) | 0.18 (0.01-0.46) | 0 (0-1) | 0.15 (0.01-0.37) | -0.89 (-1.05--0.73) |
| Angola | 50 (8-104) | 1.32 (0.21-2.66) | 80 (9-169) | 0.79 (0.09-1.65) | -2.33 (-2.53--2.13) |
| Antigua and Barbuda | 0 (0-0) | 0.29 (0.03-0.58) | 0 (0-1) | 0.27 (0.03-0.53) | -0.29 (-0.51--0.06) |
| Argentina | 195 (15-394) | 0.62 (0.05-1.24) | 185 (8-392) | 0.34 (0.01-0.72) | -2.33 (-2.47--2.19) |
| Armenia | 1 (0-2) | 0.03 (0-0.08) | 0 (0-0) | 0 (0-0.01) | -10.28 (-11.57--8.97) |
| Australia | 59 (3-123) | 0.3 (0.01-0.63) | 107 (5-229) | 0.25 (0.01-0.53) | -0.71 (-0.78--0.64) |
| Austria | 28 (2-57) | 0.25 (0.01-0.5) | 24 (1-52) | 0.14 (0.01-0.3) | -2.41 (-2.67--2.16) |
| Azerbaijan | 17 (1-43) | 0.36 (0.02-0.89) | 3 (1-9) | 0.05 (0.02-0.15) | -9.33 (-10.89--7.74) |
| Bahamas | 0 (0-1) | 0.29 (0.01-0.66) | 1 (0-3) | 0.33 (0.01-0.71) | 0.55 (0.47-0.63) |
| Bahrain | 0 (0-0) | 0.14 (0.01-0.35) | 0 (0-1) | 0.04 (0-0.11) | -4.86 (-5.17--4.55) |
| Bangladesh | 331 (63-630) | 0.72 (0.13-1.37) | 528 (91-1118) | 0.42 (0.07-0.87) | -1.88 (-2.02--1.74) |
| Barbados | 1 (0-3) | 0.51 (0.05-1.01) | 2 (0-4) | 0.41 (0.03-0.85) | -1.06 (-1.25--0.87) |
| Belarus | 23 (1-51) | 0.18 (0.01-0.39) | 6 (1-20) | 0.04 (0.01-0.13) | -6.83 (-7.71--5.94) |
| Belgium | 35 (1-74) | 0.23 (0.01-0.49) | 37 (2-88) | 0.16 (0.01-0.38) | -1.44 (-1.57--1.3) |
| Belize | 0 (0-0) | 0.2 (0.03-0.39) | 1 (0-1) | 0.23 (0.03-0.45) | 0.41 (0.11-0.71) |
| Benin | 7 (1-13) | 0.35 (0.04-0.7) | 20 (2-42) | 0.43 (0.04-0.91) | 1.24 (1.05-1.42) |
| Bermuda | 0 (0-0) | 0.33 (0.02-0.75) | 0 (0-1) | 0.26 (0.01-0.57) | -0.61 (-0.68--0.54) |
| Bhutan | 1 (0-3) | 0.58 (0.1-1.12) | 2 (0-5) | 0.44 (0.05-0.92) | -0.96 (-1.1--0.83) |
| Bolivia (Plurinational State of) | 8 (1-16) | 0.27 (0.04-0.54) | 21 (3-42) | 0.25 (0.04-0.52) | -0.24 (-0.33--0.16) |
| Bosnia and Herzegovina | 6 (0-13) | 0.15 (0.01-0.32) | 3 (0-7) | 0.04 (0-0.12) | -5.08 (-5.46--4.71) |
| Botswana | 9 (1-17) | 1.53 (0.24-3.07) | 17 (3-35) | 1.3 (0.2-2.6) | -1.18 (-1.6--0.76) |
| Brazil | 588 (93-1139) | 0.68 (0.11-1.31) | 1045 (94-2097) | 0.44 (0.04-0.88) | -1.55 (-1.61--1.48) |
| Brunei Darussalam | 0 (0-1) | 0.43 (0.04-0.87) | 1 (0-1) | 0.26 (0.03-0.53) | -1.91 (-2.15--1.68) |
| Bulgaria | 8 (1-22) | 0.07 (0.01-0.17) | 2 (0-6) | 0.02 (0-0.04) | -6.07 (-6.56--5.57) |
| Burkina Faso | 17 (3-32) | 0.41 (0.07-0.77) | 48 (7-93) | 0.55 (0.08-1.07) | 1.38 (1.19-1.57) |
| Burundi | 45 (7-90) | 1.94 (0.31-3.9) | 58 (10-120) | 1.3 (0.23-2.65) | -1.82 (-2--1.65) |
| Cabo Verde | 2 (0-4) | 0.97 (0.16-1.88) | 4 (0-8) | 0.91 (0.05-1.99) | -1.75 (-2.36--1.14) |
| Cambodia | 17 (3-34) | 0.39 (0.06-0.77) | 36 (6-70) | 0.31 (0.05-0.63) | -0.9 (-1.02--0.78) |
| Cameroon | 17 (2-35) | 0.41 (0.06-0.82) | 46 (2-106) | 0.41 (0.02-0.93) | -0.02 (-0.1-0.06) |
| Canada | 61 (3-133) | 0.19 (0.01-0.41) | 142 (6-310) | 0.2 (0.01-0.44) | 0.55 (0.36-0.75) |
| Central African Republic | 19 (3-38) | 1.64 (0.3-3.24) | 27 (5-55) | 1.28 (0.23-2.54) | -0.98 (-1.06--0.89) |
| Chad | 11 (2-20) | 0.38 (0.07-0.72) | 30 (5-59) | 0.57 (0.09-1.11) | 1.82 (1.63-2.02) |
| Chile | 50 (2-105) | 0.55 (0.02-1.16) | 48 (2-109) | 0.2 (0.01-0.45) | -3.84 (-4--3.69) |
| China | 11664 (590-25110) | 1.46 (0.07-3.19) | 1049 (479-2347) | 0.06 (0.02-0.14) | -11.61 (-11.96--11.26) |
| Colombia | 61 (5-124) | 0.39 (0.04-0.77) | 91 (9-188) | 0.17 (0.02-0.35) | -3.26 (-3.45--3.08) |
| Comoros | 4 (1-8) | 1.75 (0.3-3.57) | 6 (1-13) | 1.36 (0.26-2.64) | -1.17 (-1.35--1) |
| Congo | 18 (3-35) | 1.73 (0.27-3.43) | 29 (5-57) | 1.19 (0.19-2.36) | -1.56 (-1.74--1.39) |
| Cook Islands | 0 (0-0) | 0.34 (0.06-0.67) | 0 (0-0) | 0.24 (0.03-0.48) | -1.35 (-1.47--1.23) |
| Costa Rica | 5 (1-10) | 0.31 (0.05-0.58) | 8 (1-17) | 0.17 (0.02-0.34) | -2.54 (-2.8--2.27) |
| Croatia | 13 (1-29) | 0.21 (0.01-0.45) | 13 (1-30) | 0.16 (0.01-0.36) | -1.08 (-1.2--0.95) |
| Cuba | 31 (2-63) | 0.31 (0.02-0.62) | 45 (2-106) | 0.24 (0.01-0.55) | -1.15 (-1.42--0.89) |
| Cyprus | 1 (0-1) | 0.09 (0-0.19) | 2 (0-4) | 0.09 (0-0.19) | 0.3 (0.02-0.58) |
| Czechia | 27 (1-56) | 0.2 (0.01-0.42) | 42 (2-93) | 0.21 (0.01-0.47) | 0.26 (0.11-0.4) |
| Côte d'Ivoire | 15 (2-30) | 0.4 (0.06-0.78) | 51 (7-106) | 0.51 (0.07-1.04) | 1.1 (0.95-1.24) |
| Democratic People's Republic of Korea | 51 (3-141) | 0.35 (0.02-0.92) | 162 (7-366) | 0.5 (0.02-1.14) | 1.18 (0.8-1.57) |
| Democratic Republic of the Congo | 187 (33-379) | 1.25 (0.22-2.55) | 339 (56-706) | 1.01 (0.17-2.08) | -0.94 (-1.02--0.87) |
| Denmark | 29 (1-60) | 0.36 (0.02-0.75) | 26 (1-59) | 0.23 (0.01-0.51) | -1.91 (-2.05--1.77) |
| Djibouti | 2 (0-4) | 1.37 (0.2-2.83) | 6 (1-13) | 1.09 (0.12-2.3) | -1.14 (-1.32--0.96) |
| Dominica | 0 (0-1) | 0.38 (0.02-0.79) | 0 (0-1) | 0.37 (0.02-0.79) | 0 (-0.24-0.25) |
| Dominican Republic | 6 (1-12) | 0.17 (0.03-0.32) | 19 (1-41) | 0.2 (0.02-0.45) | 1.22 (1.03-1.41) |
| Ecuador | 12 (2-22) | 0.24 (0.04-0.45) | 24 (4-49) | 0.17 (0.03-0.35) | -1.01 (-1.21--0.8) |
| Egypt | 3 (1-8) | 0.01 (0-0.03) | 2 (1-3) | 0 (0-0.01) | -3.63 (-4.5--2.75) |
| El Salvador | 4 (1-8) | 0.15 (0.03-0.29) | 9 (1-18) | 0.14 (0.01-0.29) | -0.84 (-1.2--0.48) |
| Equatorial Guinea | 3 (0-6) | 1.38 (0.22-2.76) | 4 (1-9) | 0.94 (0.15-2.05) | -1.65 (-1.94--1.36) |
| Eritrea | 17 (3-35) | 1.7 (0.32-3.46) | 39 (6-80) | 1.5 (0.25-3.05) | -0.68 (-0.89--0.47) |
| Estonia | 5 (0-10) | 0.25 (0.01-0.51) | 4 (0-8) | 0.14 (0.01-0.33) | -2.27 (-2.45--2.09) |
| Eswatini | 5 (1-10) | 1.76 (0.29-3.54) | 10 (2-19) | 1.71 (0.29-3.36) | -0.25 (-0.86-0.37) |
| Ethiopia | 104 (19-210) | 0.52 (0.1-1.04) | 119 (23-245) | 0.31 (0.06-0.63) | -2.1 (-2.23--1.97) |
| Fiji | 1 (0-2) | 0.27 (0.05-0.52) | 2 (0-4) | 0.27 (0.04-0.56) | 0.33 (0.01-0.65) |
| Finland | 18 (1-36) | 0.25 (0.02-0.5) | 21 (1-44) | 0.17 (0.01-0.35) | -1.08 (-1.24--0.92) |
| France | 358 (14-770) | 0.45 (0.02-0.98) | 366 (18-770) | 0.28 (0.01-0.58) | -1.88 (-1.95--1.81) |
| Gabon | 7 (1-15) | 1.36 (0.21-2.76) | 11 (2-23) | 1.13 (0.18-2.28) | -0.87 (-1--0.74) |
| Gambia | 1 (0-1) | 0.19 (0.03-0.35) | 2 (0-4) | 0.24 (0.04-0.46) | 0.7 (0.54-0.86) |
| Georgia | 5 (0-11) | 0.08 (0-0.18) | 6 (0-14) | 0.1 (0-0.24) | 1.27 (1.1-1.43) |
| Germany | 263 (12-548) | 0.22 (0.01-0.45) | 435 (18-927) | 0.24 (0.01-0.51) | 0.23 (-0.02-0.48) |
| Ghana | 19 (3-37) | 0.31 (0.05-0.61) | 48 (6-97) | 0.31 (0.04-0.64) | 0.22 (0.08-0.36) |
| Greece | 1 (1-1) | 0.01 (0-0.01) | 3 (1-9) | 0.01 (0-0.03) | 1.51 (1.07-1.95) |
| Greenland | 0 (0-1) | 0.73 (0.04-1.64) | 0 (0-1) | 0.48 (0.03-1.2) | -1.79 (-2.06--1.52) |
| Grenada | 1 (0-1) | 0.8 (0.14-1.5) | 1 (0-1) | 0.67 (0.11-1.29) | -0.48 (-0.6--0.36) |
| Guam | 0 (0-0) | 0.26 (0.03-0.5) | 0 (0-1) | 0.22 (0.02-0.46) | -0.22 (-0.7-0.26) |
| Guatemala | 6 (1-11) | 0.17 (0.03-0.33) | 20 (2-41) | 0.19 (0.02-0.4) | -0.09 (-0.6-0.43) |
| Guinea | 5 (1-10) | 0.16 (0.02-0.32) | 10 (2-21) | 0.2 (0.03-0.39) | 0.96 (0.82-1.11) |
| Guinea-Bissau | 2 (0-5) | 0.62 (0.1-1.23) | 5 (1-10) | 0.72 (0.11-1.44) | 0.98 (0.79-1.17) |
| Guyana | 1 (0-2) | 0.25 (0.04-0.47) | 1 (0-2) | 0.18 (0.02-0.38) | -1.11 (-1.3--0.92) |
| Haiti | 16 (3-32) | 0.51 (0.09-1.02) | 28 (5-58) | 0.42 (0.07-0.88) | -0.53 (-0.68--0.38) |
| Honduras | 3 (0-5) | 0.13 (0.02-0.24) | 9 (1-18) | 0.16 (0.02-0.32) | 1.04 (0.84-1.24) |
| Hungary | 35 (1-74) | 0.24 (0.01-0.52) | 18 (1-50) | 0.1 (0.01-0.27) | -4 (-4.36--3.63) |
| Iceland | 1 (0-2) | 0.41 (0.05-0.81) | 2 (0-3) | 0.29 (0.02-0.59) | -1.46 (-1.61--1.32) |
| India | 1650 (206-3290) | 0.39 (0.05-0.77) | 3114 (288-6500) | 0.28 (0.03-0.58) | -1.37 (-1.53--1.21) |
| Indonesia | 253 (41-484) | 0.27 (0.04-0.51) | 442 (52-898) | 0.22 (0.03-0.45) | -0.6 (-0.66--0.55) |
| Iran (Islamic Republic of) | 74 (3-167) | 0.33 (0.02-0.75) | 49 (7-123) | 0.08 (0.01-0.2) | -5.73 (-6.2--5.27) |
| Iraq | 3 (0-7) | 0.04 (0-0.1) | 6 (1-16) | 0.03 (0-0.08) | -2.25 (-2.81--1.69) |
| Ireland | 22 (1-46) | 0.54 (0.03-1.12) | 24 (1-56) | 0.32 (0.01-0.74) | -1.95 (-2.09--1.81) |
| Israel | 1 (0-3) | 0.02 (0-0.07) | 1 (0-3) | 0.01 (0-0.02) | -3.89 (-4.81--2.95) |
| Italy | 50 (6-123) | 0.06 (0.01-0.14) | 110 (6-250) | 0.07 (0-0.17) | 1.35 (1.15-1.55) |
| Jamaica | 5 (0-9) | 0.26 (0.02-0.52) | 6 (0-13) | 0.21 (0.01-0.44) | -0.93 (-1.24--0.62) |
| Japan | 302 (18-688) | 0.18 (0.01-0.4) | 241 (29-633) | 0.06 (0.01-0.17) | -3.05 (-3.45--2.65) |
| Jordan | 1 (0-2) | 0.08 (0-0.18) | 3 (0-8) | 0.06 (0-0.14) | -1.69 (-1.92--1.46) |
| Kazakhstan | 138 (6-307) | 1.15 (0.05-2.56) | 9 (2-24) | 0.07 (0.02-0.18) | -11.39 (-12.33--10.45) |
| Kenya | 79 (12-162) | 1 (0.15-2.05) | 241 (27-513) | 1.19 (0.13-2.54) | 1.03 (0.8-1.27) |
| Kiribati | 0 (0-1) | 0.85 (0.14-1.72) | 1 (0-1) | 0.81 (0.13-1.66) | -0.33 (-0.45--0.21) |
| Kuwait | 0 (0-1) | 0.04 (0-0.11) | 0 (0-1) | 0.02 (0-0.06) | -3.14 (-4.77--1.49) |
| Kyrgyzstan | 8 (1-21) | 0.28 (0.02-0.69) | 3 (0-8) | 0.07 (0.01-0.21) | -6.13 (-6.81--5.45) |
| Lao People's Democratic Republic | 9 (2-18) | 0.45 (0.07-0.86) | 8 (1-18) | 0.21 (0.02-0.43) | -3.17 (-3.38--2.96) |
| Latvia | 5 (0-12) | 0.15 (0.01-0.34) | 6 (0-14) | 0.15 (0.01-0.36) | -0.31 (-0.65-0.03) |
| Lebanon | 0 (0-0) | 0 (0-0.01) | 0 (0-1) | 0 (0-0.01) | 0.92 (0.54-1.31) |
| Lesotho | 13 (2-25) | 1.32 (0.24-2.56) | 20 (3-41) | 1.64 (0.25-3.26) | 1.07 (0.83-1.31) |
| Liberia | 5 (1-9) | 0.42 (0.07-0.8) | 11 (2-21) | 0.56 (0.09-1.09) | 1.49 (1.19-1.79) |
| Libya | 1 (0-2) | 0.04 (0-0.11) | 3 (0-8) | 0.07 (0-0.16) | 1.38 (0.97-1.8) |
| Lithuania | 9 (0-19) | 0.2 (0.01-0.42) | 9 (0-20) | 0.16 (0.01-0.39) | -0.83 (-1.1--0.56) |
| Luxembourg | 2 (0-4) | 0.33 (0.01-0.69) | 2 (0-4) | 0.18 (0.01-0.4) | -2.34 (-2.45--2.23) |
| Madagascar | 69 (13-137) | 1.36 (0.26-2.7) | 122 (25-235) | 1.14 (0.23-2.21) | -0.85 (-1.03--0.67) |
| Malawi | 92 (18-175) | 2.44 (0.47-4.62) | 194 (39-384) | 2.71 (0.53-5.3) | 0.43 (0.11-0.75) |
| Malaysia | 26 (4-49) | 0.3 (0.05-0.57) | 55 (5-115) | 0.22 (0.02-0.46) | -1.48 (-1.67--1.29) |
| Maldives | 0 (0-1) | 0.37 (0.04-0.8) | 0 (0-1) | 0.14 (0.01-0.3) | -4.12 (-4.4--3.84) |
| Mali | 12 (2-22) | 0.28 (0.05-0.53) | 23 (4-44) | 0.28 (0.05-0.53) | -0.06 (-0.11-0) |
| Malta | 1 (0-1) | 0.15 (0.01-0.33) | 0 (0-1) | 0.04 (0-0.11) | -4.34 (-4.58--4.1) |
| Marshall Islands | 0 (0-0) | 0.35 (0.06-0.68) | 0 (0-0) | 0.3 (0.05-0.61) | -0.42 (-0.51--0.34) |
| Mauritania | 5 (1-9) | 0.51 (0.09-0.97) | 9 (1-18) | 0.47 (0.07-0.93) | 0.16 (-0.06-0.37) |
| Mauritius | 2 (0-4) | 0.3 (0.04-0.6) | 3 (0-7) | 0.18 (0.01-0.39) | -1.81 (-1.99--1.63) |
| Mexico | 71 (7-141) | 0.18 (0.02-0.36) | 132 (10-270) | 0.12 (0.01-0.24) | -1.65 (-1.89--1.42) |
| Micronesia (Federated States of) | 0 (0-0) | 0.38 (0.07-0.75) | 0 (0-0) | 0.34 (0.06-0.69) | -0.61 (-0.67--0.55) |
| Monaco | 0 (0-0) | 0.06 (0.01-0.17) | 0 (0-0) | 0.03 (0.01-0.09) | -1.43 (-1.81--1.05) |
| Mongolia | 28 (5-55) | 3 (0.56-5.82) | 40 (5-80) | 2.21 (0.29-4.47) | -1.57 (-1.87--1.28) |
| Montenegro | 0 (0-0) | 0.01 (0-0.04) | 0 (0-0) | 0.01 (0-0.02) | -5.26 (-6.73--3.76) |
| Morocco | 14 (1-30) | 0.11 (0-0.23) | 14 (1-36) | 0.05 (0-0.13) | -3.29 (-3.81--2.77) |
| Mozambique | 44 (8-83) | 0.79 (0.15-1.49) | 93 (15-177) | 0.9 (0.15-1.72) | 0.73 (0.63-0.84) |
| Myanmar | 83 (14-173) | 0.36 (0.06-0.75) | 81 (5-173) | 0.18 (0.01-0.39) | -2.68 (-2.8--2.56) |
| Namibia | 2 (0-4) | 0.29 (0.06-0.55) | 4 (1-7) | 0.27 (0.04-0.54) | -0.53 (-0.81--0.25) |
| Nauru | 0 (0-0) | 0.37 (0.06-0.71) | 0 (0-0) | 0.33 (0.05-0.66) | -0.62 (-0.98--0.26) |
| Nepal | 51 (9-99) | 0.55 (0.1-1.06) | 92 (9-185) | 0.43 (0.04-0.87) | -0.8 (-1.15--0.44) |
| Netherlands | 63 (3-131) | 0.32 (0.02-0.66) | 169 (8-350) | 0.49 (0.02-1.01) | 1.64 (1.29-1.99) |
| New Zealand | 11 (0-23) | 0.27 (0.01-0.58) | 19 (1-40) | 0.23 (0.01-0.5) | -0.45 (-0.55--0.35) |
| Nicaragua | 2 (0-4) | 0.14 (0.03-0.26) | 6 (1-11) | 0.15 (0.02-0.28) | -0.41 (-0.7--0.12) |
| Niger | 8 (1-17) | 0.32 (0.04-0.63) | 24 (2-51) | 0.33 (0.03-0.7) | 0.49 (0.27-0.71) |
| Nigeria | 35 (4-84) | 0.08 (0.01-0.2) | 59 (4-148) | 0.08 (0.01-0.18) | -0.33 (-0.45--0.22) |
| Niue | 0 (0-0) | 0.27 (0.05-0.54) | 0 (0-0) | 0.25 (0.04-0.49) | -0.61 (-0.75--0.47) |
| North Macedonia | 0 (0-1) | 0.01 (0-0.04) | 0 (0-1) | 0.01 (0-0.03) | -2.66 (-3.64--1.66) |
| Northern Mariana Islands | 0 (0-0) | 0.15 (0.02-0.34) | 0 (0-0) | 0.28 (0.04-0.56) | 3.19 (2.76-3.63) |
| Norway | 14 (1-28) | 0.21 (0.02-0.41) | 18 (1-37) | 0.18 (0.01-0.38) | -0.26 (-0.34--0.17) |
| Oman | 1 (0-2) | 0.13 (0.01-0.29) | 1 (0-3) | 0.1 (0.01-0.24) | -0.6 (-0.71--0.49) |
| Pakistan | 436 (81-832) | 0.78 (0.14-1.47) | 883 (137-1742) | 0.82 (0.13-1.58) | 0.05 (-0.13-0.24) |
| Palau | 0 (0-0) | 0.25 (0.04-0.5) | 0 (0-0) | 0.24 (0.04-0.48) | -0.22 (-0.33--0.12) |
| Palestine | 1 (0-3) | 0.16 (0.03-0.31) | 1 (0-3) | 0.06 (0-0.13) | -4.78 (-5.47--4.09) |
| Panama | 3 (0-5) | 0.18 (0.03-0.35) | 7 (1-13) | 0.16 (0.02-0.32) | -0.15 (-0.37-0.07) |
| Papua New Guinea | 3 (0-7) | 0.18 (0.02-0.41) | 8 (1-17) | 0.19 (0.03-0.41) | 0.14 (0.07-0.21) |
| Paraguay | 7 (1-13) | 0.32 (0.05-0.63) | 20 (3-41) | 0.37 (0.05-0.75) | 0.34 (0.18-0.5) |
| Peru | 21 (3-39) | 0.18 (0.03-0.35) | 38 (4-80) | 0.12 (0.01-0.25) | -1.48 (-1.61--1.35) |
| Philippines | 44 (7-86) | 0.16 (0.02-0.3) | 85 (7-175) | 0.11 (0.01-0.23) | -1.35 (-1.57--1.13) |
| Poland | 56 (3-129) | 0.13 (0.01-0.3) | 38 (5-96) | 0.05 (0.01-0.13) | -3.18 (-3.41--2.96) |
| Portugal | 18 (1-46) | 0.13 (0.01-0.34) | 14 (1-37) | 0.06 (0.01-0.16) | -1.91 (-2.36--1.45) |
| Puerto Rico | 26 (4-49) | 0.73 (0.12-1.37) | 18 (3-37) | 0.25 (0.04-0.5) | -4.22 (-4.49--3.94) |
| Qatar | 0 (0-0) | 0.05 (0.01-0.16) | 0 (0-0) | 0.02 (0.01-0.08) | -4.58 (-5.24--3.91) |
| Republic of Korea | 11 (3-31) | 0.06 (0.01-0.14) | 52 (5-137) | 0.06 (0.01-0.16) | -0.07 (-0.26-0.12) |
| Republic of Moldova | 4 (0-10) | 0.09 (0.01-0.22) | 6 (0-14) | 0.11 (0-0.23) | 0.18 (-0.1-0.46) |
| Romania | 7 (1-19) | 0.02 (0-0.07) | 2 (1-3) | 0.01 (0-0.01) | -6.89 (-8.13--5.64) |
| Russian Federation | 398 (20-882) | 0.22 (0.01-0.49) | 416 (20-921) | 0.18 (0.01-0.39) | -0.29 (-0.93-0.36) |
| Rwanda | 63 (11-123) | 2.19 (0.37-4.26) | 66 (12-134) | 1.16 (0.21-2.34) | -3.16 (-3.52--2.81) |
| Saint Kitts and Nevis | 0 (0-0) | 0.51 (0.08-1) | 0 (0-0) | 0.38 (0.04-0.74) | -1.07 (-1.41--0.72) |
| Saint Lucia | 0 (0-1) | 0.57 (0.1-1.08) | 1 (0-2) | 0.44 (0.07-0.84) | -1.06 (-1.39--0.72) |
| Saint Vincent and the Grenadines | 0 (0-0) | 0.25 (0.04-0.47) | 0 (0-1) | 0.23 (0.03-0.45) | -0.38 (-0.76-0) |
| Samoa | 0 (0-0) | 0.25 (0.05-0.47) | 0 (0-0) | 0.19 (0.04-0.35) | -1.34 (-1.43--1.25) |
| San Marino | 0 (0-0) | 0.08 (0-0.18) | 0 (0-0) | 0.07 (0-0.18) | -0.38 (-0.82-0.06) |
| Sao Tome and Principe | 0 (0-0) | 0.33 (0.06-0.64) | 1 (0-1) | 0.52 (0.08-1.03) | 1.99 (1.86-2.12) |
| Saudi Arabia | 5 (0-13) | 0.1 (0-0.24) | 16 (1-37) | 0.1 (0-0.23) | -0.29 (-0.67-0.09) |
| Senegal | 12 (2-24) | 0.39 (0.06-0.75) | 31 (3-64) | 0.44 (0.05-0.9) | 1.06 (0.65-1.48) |
| Serbia | 15 (1-33) | 0.13 (0.01-0.29) | 18 (1-43) | 0.12 (0.01-0.28) | -0.8 (-1.08--0.52) |
| Seychelles | 0 (0-1) | 0.59 (0.1-1.12) | 1 (0-1) | 0.54 (0.09-1.05) | -0.48 (-0.62--0.33) |
| Sierra Leone | 7 (1-13) | 0.35 (0.06-0.68) | 15 (2-31) | 0.45 (0.07-0.92) | 1.37 (1.14-1.6) |
| Singapore | 6 (0-14) | 0.31 (0.01-0.68) | 5 (0-11) | 0.06 (0-0.15) | -5.65 (-5.94--5.35) |
| Slovakia | 13 (1-29) | 0.23 (0.01-0.49) | 14 (1-34) | 0.15 (0.01-0.37) | -1.47 (-1.74--1.2) |
| Slovenia | 6 (0-14) | 0.26 (0.01-0.57) | 7 (0-15) | 0.16 (0.01-0.36) | -2.1 (-2.28--1.93) |
| Solomon Islands | 1 (0-1) | 0.42 (0.07-0.82) | 1 (0-2) | 0.4 (0.07-0.77) | -0.17 (-0.29--0.05) |
| Somalia | 52 (10-104) | 2.04 (0.37-4.02) | 100 (18-204) | 1.54 (0.27-3.14) | -1.01 (-1.07--0.94) |
| South Africa | 264 (35-533) | 1.27 (0.17-2.57) | 402 (51-796) | 0.95 (0.12-1.86) | -1.78 (-2.43--1.13) |
| South Sudan | 34 (5-69) | 1.49 (0.25-3.02) | 40 (6-82) | 1.09 (0.18-2.26) | -1.3 (-1.38--1.23) |
| Spain | 28 (4-76) | 0.05 (0.01-0.14) | 94 (5-224) | 0.1 (0.01-0.24) | 2.77 (2.32-3.22) |
| Sri Lanka | 53 (9-101) | 0.52 (0.09-1) | 98 (11-203) | 0.4 (0.05-0.82) | -0.36 (-0.78-0.07) |
| Sudan | 32 (2-73) | 0.36 (0.03-0.83) | 62 (3-153) | 0.36 (0.02-0.88) | -0.1 (-0.22-0.01) |
| Suriname | 0 (0-1) | 0.13 (0.02-0.26) | 1 (0-2) | 0.13 (0.02-0.26) | -0.12 (-0.41-0.17) |
| Sweden | 33 (3-66) | 0.21 (0.02-0.43) | 40 (2-85) | 0.18 (0.01-0.39) | -0.57 (-0.68--0.46) |
| Switzerland | 33 (1-69) | 0.32 (0.01-0.67) | 38 (2-81) | 0.22 (0.01-0.46) | -1.12 (-1.22--1.01) |
| Syrian Arab Republic | 2 (0-4) | 0.04 (0-0.09) | 7 (0-16) | 0.06 (0-0.14) | 1.16 (0.82-1.5) |
| Taiwan (Province of China) | 54 (2-114) | 0.34 (0.01-0.72) | 82 (6-223) | 0.21 (0.02-0.57) | -1.41 (-1.59--1.24) |
| Tajikistan | 9 (1-23) | 0.34 (0.03-0.86) | 5 (1-17) | 0.15 (0.01-0.47) | -4.27 (-5.74--2.78) |
| Thailand | 141 (22-280) | 0.41 (0.07-0.82) | 273 (30-577) | 0.27 (0.03-0.56) | -2.08 (-2.34--1.81) |
| Timor-Leste | 1 (0-2) | 0.3 (0.05-0.56) | 2 (0-4) | 0.26 (0.05-0.53) | -0.48 (-0.72--0.23) |
| Togo | 5 (1-9) | 0.4 (0.08-0.76) | 19 (3-37) | 0.54 (0.09-1.05) | 1.38 (1.21-1.55) |
| Tokelau | 0 (0-0) | 0.25 (0.04-0.5) | 0 (0-0) | 0.2 (0.04-0.4) | -0.83 (-0.86--0.8) |
| Tonga | 0 (0-0) | 0.23 (0.04-0.48) | 0 (0-0) | 0.23 (0.04-0.45) | -0.38 (-0.7--0.06) |
| Trinidad and Tobago | 2 (0-4) | 0.25 (0.04-0.47) | 3 (0-6) | 0.15 (0.02-0.3) | -2.42 (-2.79--2.05) |
| Tunisia | 2 (0-5) | 0.05 (0-0.11) | 2 (0-5) | 0.02 (0-0.05) | -4.74 (-5.1--4.38) |
| Turkey | 2 (1-3) | 0.01 (0-0.01) | 3 (2-4) | 0 (0-0) | -1.43 (-1.63--1.23) |
| Turkmenistan | 21 (1-52) | 1.18 (0.07-2.89) | 1 (1-3) | 0.04 (0.02-0.08) | -13.67 (-14.64--12.68) |
| Tuvalu | 0 (0-0) | 0.33 (0.06-0.65) | 0 (0-0) | 0.26 (0.04-0.5) | -1.03 (-1.11--0.96) |
| Uganda | 94 (19-178) | 1.51 (0.3-2.85) | 237 (41-463) | 1.76 (0.31-3.41) | 0.48 (0.32-0.64) |
| Ukraine | 92 (5-218) | 0.13 (0.01-0.3) | 32 (4-94) | 0.04 (0.01-0.13) | -6.48 (-7.49--5.46) |
| United Arab Emirates | 0 (0-0) | 0.03 (0.01-0.09) | 16 (1-47) | 0.4 (0.01-1.19) | 11.01 (9.54-12.49) |
| United Kingdom | 469 (33-975) | 0.51 (0.04-1.06) | 644 (42-1340) | 0.49 (0.03-1.03) | -0.32 (-0.42--0.22) |
| United Republic of Tanzania | 171 (32-342) | 1.62 (0.31-3.27) | 290 (50-600) | 1.23 (0.22-2.5) | -1.15 (-1.25--1.06) |
| United States of America | 544 (28-1190) | 0.17 (0.01-0.38) | 1216 (97-2491) | 0.22 (0.02-0.44) | 1.13 (0.92-1.35) |
| United States Virgin Islands | 0 (0-1) | 0.31 (0.03-0.63) | 1 (0-1) | 0.32 (0.02-0.66) | 0.57 (0.26-0.87) |
| Uruguay | 37 (6-70) | 0.94 (0.15-1.8) | 30 (3-59) | 0.53 (0.05-1.06) | -2.13 (-2.26--1.99) |
| Uzbekistan | 30 (4-82) | 0.28 (0.03-0.77) | 3 (2-3) | 0.02 (0.01-0.04) | -12.43 (-13.83--11) |
| Vanuatu | 0 (0-0) | 0.28 (0.04-0.59) | 0 (0-1) | 0.28 (0.05-0.56) | -0.2 (-0.32--0.08) |
| Venezuela (Bolivarian Republic of) | 24 (4-46) | 0.26 (0.04-0.51) | 49 (4-103) | 0.17 (0.02-0.36) | -1.73 (-1.98--1.49) |
| Viet Nam | 91 (14-180) | 0.23 (0.04-0.45) | 136 (7-319) | 0.15 (0.01-0.34) | -1.9 (-2.27--1.52) |
| Yemen | 20 (2-43) | 0.43 (0.05-0.95) | 54 (7-116) | 0.44 (0.06-0.96) | -0.06 (-0.1--0.01) |
| Zambia | 55 (9-105) | 1.96 (0.32-3.78) | 103 (17-200) | 1.58 (0.26-3.04) | -1.18 (-1.45--0.9) |
| Zimbabwe | 70 (14-129) | 1.79 (0.35-3.31) | 125 (22-233) | 1.84 (0.33-3.44) | 0.04 (-0.19-0.27) |

**Table S4.** The number of DALYs cases and the age-standardized DALYs rate of esophageal cancer attributable to diet low in vegetables in 1990 and 2019, and its trends from 1990 to 2019 globally. Abbreviations: DALYs, disability-adjusted life years.

|  | Number of DALYs cases (95% UI) in 1990 | The age-standardized DALYs rate/100000 (95% UI) in 1990 | Number of DALYs cases (95% UI) in 2019 | The age-standardized DALYs rate/100000 (95% UI) in 2019 | EAPC (95% CI) |
| --- | --- | --- | --- | --- | --- |
| Global | 550996 (57581-1145982) | 13.41 (1.41-27.88) | 420309 (64154-827694) | 5.03 (0.77-9.91) | -3.64 (-3.91--3.36) |
| **Sex** |  |  |  |  |  |
| Female | 171401 (19849-350757) | 7.96 (0.92-16.31) | 131412 (20890-258144) | 3.03 (0.48-5.95) | -3.61 (-3.92--3.3) |
| Male | 379595 (37236-789676) | 19.45 (1.92-40.34) | 288897 (43912-575697) | 7.23 (1.09-14.42) | -3.66 (-3.91--3.4) |
| **Age** |  |  |  |  |  |
| <24 years | 0 (0-0) | 0 (0-0) | 0 (0-0) | 0 (0-0) | 0 (0-0) |
| 25-29 years | 3175 (463-6206) | 0.72 (0.1-1.4) | 2803 (434-5495) | 0.46 (0.07-0.91) | -1.97 (-2.14--1.81) |
| 30-34 years | 4494 (698-9021) | 1.17 (0.18-2.34) | 5145 (814-10003) | 0.86 (0.14-1.66) | -1.23 (-1.36--1.09) |
| 35-39 years | 10758 (1466-22039) | 3.05 (0.42-6.25) | 10443 (1641-20222) | 1.93 (0.3-3.74) | -1.58 (-1.75--1.4) |
| 40-44 years | 28533 (3108-59042) | 9.97 (1.09-20.62) | 20708 (3414-40403) | 4.2 (0.69-8.19) | -3.56 (-3.95--3.16) |
| 45-49 years | 42290 (4837-86381) | 18.2 (2.08-37.17) | 35332 (5552-68956) | 7.46 (1.17-14.55) | -3.52 (-3.78--3.27) |
| 50-54 years | 67533 (7403-136842) | 31.77 (3.48-64.37) | 53744 (8516-105474) | 12.3 (1.95-24.15) | -3.46 (-3.71--3.2) |
| 55-59 years | 87840 (8734-182079) | 47.37 (4.71-98.2) | 64611 (9683-127847) | 17.41 (2.61-34.46) | -3.5 (-3.88--3.13) |
| 60-64 years | 92877 (9394-192940) | 57.82 (5.85-120.1) | 63096 (9680-123180) | 20.19 (3.1-39.41) | -3.87 (-4.21--3.54) |
| 65-69 years | 83662 (8327-175920) | 67.75 (6.74-142.46) | 57132 (8890-111976) | 22.09 (3.44-43.3) | -4.03 (-4.34--3.71) |
| 70-74 years | 64538 (6458-134231) | 76.36 (7.64-158.83) | 44918 (6987-86846) | 24.01 (3.73-46.42) | -4.24 (-4.6--3.89) |
| 75-79 years | 39394 (3991-80761) | 64.25 (6.51-131.73) | 29961 (4485-59672) | 23.58 (3.53-46.97) | -4.08 (-4.34--3.81) |
| 80-84 years | 18863 (1918-38593) | 53.56 (5.45-109.58) | 19055 (2647-38572) | 22.57 (3.14-45.69) | -3.43 (-3.64--3.22) |
| 85-89 years | 5696 (640-11943) | 37.8 (4.25-79.26) | 9764 (1316-20393) | 22.46 (3.03-46.9) | -1.94 (-2.2--1.67) |
| 90-94 years | 1153 (123-2392) | 26.16 (2.79-54.3) | 2927 (332-6145) | 17.36 (1.97-36.45) | -1.45 (-1.57--1.33) |
| 95+ years | 191 (19-403) | 18.57 (1.87-39.18) | 671 (72-1427) | 14.06 (1.5-29.9) | -0.76 (-0.86--0.66) |
| **SDI regions** |  |  |  |  |  |
| High SDI | 59006 (3233-125758) | 5.85 (0.32-12.47) | 77836 (5104-166338) | 4.47 (0.29-9.56) | -0.81 (-0.87--0.76) |
| High-middle SDI | 123555 (7951-266011) | 11.27 (0.73-24.28) | 50118 (8331-100745) | 2.45 (0.41-4.93) | -5.6 (-6.11--5.1) |
| Middle SDI | 248321 (18676-527086) | 23.13 (1.74-48.91) | 93317 (15090-183132) | 3.62 (0.59-7.1) | -6.99 (-7.35--6.63) |
| Low-middle SDI | 75767 (12266-151010) | 11.67 (1.88-23.17) | 117224 (16243-228658) | 8.08 (1.12-15.7) | -1.41 (-1.49--1.33) |
| Low SDI | 44185 (7631-81517) | 16.98 (2.95-31.4) | 81561 (15172-157287) | 14.34 (2.67-27.67) | -0.71 (-0.79--0.64) |
| **GBD regions** |  |  |  |  |  |
| Africa | 51189 (9244-96922) | 16.44 (2.97-31.26) | 91703 (17149-178670) | 13.33 (2.49-26.02) | -0.98 (-1.15--0.81) |
| African Region | 48169 (8773-91456) | 19.77 (3.6-37.65) | 86439 (16464-168472) | 16.16 (3.08-31.47) | -0.96 (-1.14--0.77) |
| America | 42980 (4689-86842) | 7.11 (0.78-14.37) | 72635 (6266-147015) | 5.78 (0.5-11.69) | -0.68 (-0.72--0.64) |
| Andean Latin America | 958 (154-1810) | 4.56 (0.73-8.59) | 1716 (254-3471) | 3.06 (0.46-6.19) | -1.38 (-1.47--1.29) |
| Asia | 404017 (34735-849216) | 19.01 (1.64-39.89) | 198717 (33185-383360) | 3.99 (0.67-7.69) | -5.81 (-6.21--5.4) |
| Australasia | 1487 (65-3118) | 6.36 (0.28-13.34) | 2381 (98-5128) | 5.04 (0.21-10.84) | -0.79 (-0.85--0.74) |
| Caribbean | 2240 (362-4295) | 8.53 (1.38-16.33) | 3189 (435-6553) | 6.13 (0.84-12.61) | -1.28 (-1.51--1.05) |
| Central Asia | 6243 (578-14256) | 13.19 (1.23-29.89) | 1628 (357-3393) | 2.26 (0.48-4.68) | -7.72 (-8.4--7.02) |
| Central Europe | 4917 (256-10933) | 3.34 (0.17-7.43) | 3608 (327-8238) | 1.83 (0.17-4.21) | -2.5 (-2.69--2.32) |
| Central Latin America | 4227 (497-8311) | 4.94 (0.59-9.7) | 7313 (725-14709) | 3.07 (0.3-6.17) | -1.89 (-2.03--1.74) |
| Central Sub-Saharan Africa | 8149 (1360-15812) | 32.23 (5.5-62.58) | 13819 (2329-28063) | 23.43 (3.93-47.52) | -1.37 (-1.48--1.27) |
| Commonwealth High Income | 12509 (778-26110) | 8.51 (0.53-17.77) | 17405 (964-36447) | 7.1 (0.39-14.85) | -0.75 (-0.81--0.69) |
| Commonwealth Low Income | 24479 (4356-46487) | 26.02 (4.64-49.26) | 44897 (7934-91673) | 19.06 (3.35-38.58) | -1.12 (-1.2--1.04) |
| Commonwealth Middle Income | 73856 (11263-144858) | 10.87 (1.67-21.22) | 131824 (15083-258342) | 8.28 (0.95-16.2) | -1.21 (-1.41--1.02) |
| East Asia | 294635 (14415-639948) | 32.28 (1.61-69.66) | 26415 (11280-56355) | 1.28 (0.53-2.82) | -11.81 (-12.19--11.43) |
| East Asia & Pacific - WB | 322682 (19691-692105) | 22.91 (1.41-49.12) | 64351 (16092-129795) | 2.03 (0.5-4.1) | -9.02 (-9.47--8.57) |
| Eastern Europe | 13819 (715-30655) | 4.85 (0.25-10.78) | 12054 (685-27396) | 3.6 (0.2-8.15) | -1.17 (-1.64--0.69) |
| Eastern Mediterranean Region | 19252 (3205-37039) | 9.82 (1.63-18.96) | 37408 (6136-72453) | 7.76 (1.29-14.97) | -0.98 (-1.1--0.87) |
| Eastern Sub-Saharan Africa | 26218 (4832-47909) | 31.83 (5.86-58.56) | 48057 (9095-94123) | 26.67 (5.1-52.23) | -0.73 (-0.83--0.64) |
| Europe | 52054 (2923-111334) | 5.13 (0.29-10.97) | 56603 (3302-120555) | 3.92 (0.23-8.41) | -1.07 (-1.13--1.01) |
| Europe & Central Asia - WB | 57053 (3226-122352) | 5.46 (0.31-11.71) | 57052 (3500-121437) | 3.8 (0.24-8.14) | -1.46 (-1.55--1.38) |
| European Region | 57080 (3232-122402) | 5.43 (0.31-11.64) | 57068 (3510-121461) | 3.77 (0.23-8.06) | -1.48 (-1.56--1.39) |
| High-income Asia Pacific | 7234 (522-16545) | 3.51 (0.25-8.01) | 5129 (637-13386) | 1.26 (0.16-3.26) | -3.09 (-3.45--2.73) |
| High-income North America | 13704 (680-30233) | 4.13 (0.21-9.13) | 28527 (2101-58987) | 4.77 (0.35-9.87) | 0.86 (0.68-1.04) |
| Latin America & Caribbean - WB | 29867 (4177-58410) | 10.79 (1.51-21.09) | 44494 (4136-88681) | 6.56 (0.61-13.07) | -1.85 (-1.94--1.77) |
| Middle East & North Africa - WB | 3726 (291-8094) | 2.83 (0.22-6.17) | 5070 (650-11183) | 1.47 (0.19-3.24) | -2.73 (-3.05--2.41) |
| North Africa and Middle East | 6159 (729-12884) | 3.36 (0.41-7.05) | 9267 (1339-20001) | 1.96 (0.28-4.23) | -2.23 (-2.45--2.02) |
| North America | 13701 (680-30229) | 4.13 (0.21-9.13) | 28526 (2101-58980) | 4.77 (0.35-9.87) | 0.86 (0.68-1.04) |
| Oceania | 176 (30-359) | 5.36 (0.92-11) | 400 (66-824) | 5.11 (0.84-10.6) | -0.13 (-0.17--0.09) |
| Region of the Americas | 42980 (4689-86842) | 7.11 (0.78-14.37) | 72635 (6266-147015) | 5.78 (0.5-11.69) | -0.68 (-0.72--0.64) |
| South-East Asia Region | 74891 (11993-146997) | 9.71 (1.55-18.9) | 126491 (14222-251977) | 6.95 (0.79-13.83) | -1.3 (-1.39--1.2) |
| South Asia | 70444 (11322-136830) | 11.25 (1.83-21.82) | 124896 (15207-247032) | 8.34 (1.02-16.45) | -1.19 (-1.29--1.08) |
| South Asia - WB | 73461 (11805-143207) | 11.39 (1.85-22.02) | 129964 (16425-255012) | 8.45 (1.08-16.54) | -1.17 (-1.28--1.07) |
| Southeast Asia | 20007 (3155-38074) | 7.18 (1.14-13.63) | 31557 (3190-63451) | 4.87 (0.5-9.75) | -1.53 (-1.61--1.44) |
| Southern Latin America | 6303 (495-12688) | 13.59 (1.08-27.36) | 5247 (262-11117) | 6.36 (0.32-13.45) | -2.93 (-3.07--2.79) |
| Southern Sub-Saharan Africa | 10209 (1727-19984) | 34.2 (5.83-67.11) | 15147 (2572-29490) | 25.32 (4.3-49.15) | -1.69 (-2.24--1.13) |
| Sub-Saharan Africa - WB | 50339 (9125-95202) | 20.66 (3.75-39.34) | 90594 (16945-176298) | 17.22 (3.22-33.55) | -0.88 (-1.05--0.7) |
| Tropical Latin America | 16219 (2454-31369) | 16.53 (2.54-31.96) | 27144 (2430-54321) | 10.84 (0.97-21.7) | -1.54 (-1.59--1.48) |
| Western Europe | 32716 (1854-68705) | 6.07 (0.34-12.76) | 40691 (2106-86235) | 5.02 (0.25-10.73) | -0.73 (-0.83--0.63) |
| Western Pacific Region | 306975 (16338-664129) | 25.15 (1.35-54.28) | 39446 (13388-84470) | 1.45 (0.48-3.1) | -10.6 (-11.03--10.17) |
| Western Sub-Saharan Africa | 4931 (770-9476) | 5.27 (0.82-10.09) | 12126 (1976-24302) | 6 (0.99-11.88) | 0.66 (0.57-0.75) |
| World Bank High Income | 63341 (3782-135575) | 5.11 (0.3-10.95) | 85075 (5623-182742) | 4.1 (0.27-8.81) | -0.67 (-0.73--0.61) |
| World Bank Low Income | 37513 (6716-70234) | 23.06 (4.14-43.33) | 68432 (12383-132322) | 19.97 (3.61-38.21) | -0.61 (-0.68--0.54) |
| World Bank Lower Middle Income | 100253 (16040-195104) | 8.85 (1.43-17.16) | 172961 (21423-341117) | 6.82 (0.85-13.51) | -1.09 (-1.18--1) |
| World Bank Upper Middle Income | 349725 (22541-747987) | 22.2 (1.44-47.29) | 93588 (18911-180697) | 2.72 (0.54-5.32) | -7.88 (-8.3--7.45) |
| **Countries** |  |  |  |  |  |
| Afghanistan | 1620 (220-3585) | 21.41 (2.91-46.7) | 2743 (396-5886) | 18.74 (2.65-40.36) | -0.59 (-0.76--0.41) |
| Albania | 62 (3-139) | 2.83 (0.11-6.31) | 5 (2-10) | 0.11 (0.05-0.25) | -12.82 (-13.83--11.79) |
| Algeria | 300 (19-634) | 2.42 (0.16-5.08) | 422 (19-1005) | 1.24 (0.06-2.93) | -2.78 (-2.97--2.59) |
| American Samoa | 1 (0-1) | 2.89 (0.45-5.63) | 2 (0-3) | 3.21 (0.53-6.13) | 0.47 (0.31-0.64) |
| Andorra | 2 (0-6) | 4.19 (0.22-10.9) | 5 (0-12) | 3.49 (0.19-8.86) | -0.95 (-1.11--0.79) |
| Angola | 1501 (251-3099) | 33.55 (5.5-69.31) | 2271 (242-4815) | 18.27 (2.11-38.65) | -2.66 (-2.87--2.45) |
| Antigua and Barbuda | 3 (0-7) | 6.72 (0.62-13.36) | 6 (1-12) | 5.81 (0.57-11.7) | -0.5 (-0.72--0.27) |
| Argentina | 4478 (315-9070) | 13.81 (0.98-27.95) | 3866 (163-8232) | 7.3 (0.3-15.55) | -2.5 (-2.64--2.36) |
| Armenia | 13 (3-39) | 0.52 (0.11-1.56) | 3 (3-4) | 0.08 (0.06-0.11) | -10.22 (-11.63--8.78) |
| Australia | 1266 (56-2651) | 6.5 (0.29-13.65) | 2030 (84-4374) | 5.11 (0.21-11) | -0.83 (-0.88--0.77) |
| Austria | 676 (37-1372) | 6.33 (0.35-12.88) | 514 (21-1118) | 3.27 (0.14-7.11) | -2.72 (-2.98--2.46) |
| Azerbaijan | 454 (28-1139) | 8.71 (0.53-21.73) | 72 (36-176) | 0.85 (0.36-2.19) | -10.98 (-12.74--9.18) |
| Bahamas | 11 (1-27) | 6.91 (0.36-16.39) | 34 (1-73) | 7.98 (0.34-17.3) | 0.55 (0.48-0.63) |
| Bahrain | 5 (0-12) | 2.72 (0.2-7.02) | 7 (1-19) | 0.72 (0.08-2.03) | -5.4 (-5.73--5.07) |
| Bangladesh | 9289 (1796-17709) | 18.37 (3.52-34.66) | 13594 (2300-28496) | 9.98 (1.7-20.96) | -2.04 (-2.16--1.92) |
| Barbados | 32 (3-63) | 11.84 (1.16-23.72) | 44 (3-92) | 9.18 (0.65-19.2) | -1.16 (-1.35--0.97) |
| Belarus | 636 (25-1377) | 4.89 (0.19-10.64) | 149 (23-478) | 0.96 (0.15-3.13) | -7.45 (-8.43--6.45) |
| Belgium | 768 (30-1668) | 5.4 (0.21-11.77) | 695 (45-1739) | 3.36 (0.22-8.55) | -1.86 (-1.97--1.76) |
| Belize | 4 (1-8) | 4.74 (0.79-8.99) | 17 (2-33) | 5.66 (0.8-11.11) | 0.59 (0.3-0.88) |
| Benin | 170 (20-341) | 8.3 (1-16.65) | 529 (41-1127) | 10.16 (0.79-21.75) | 1.14 (0.97-1.31) |
| Bermuda | 4 (0-10) | 7.09 (0.36-16.58) | 7 (0-16) | 5.63 (0.24-12.6) | -0.58 (-0.67--0.49) |
| Bhutan | 41 (7-79) | 14.64 (2.45-28.26) | 58 (6-125) | 9.95 (1.09-21.37) | -1.36 (-1.51--1.22) |
| Bolivia (Plurinational State of) | 198 (28-399) | 5.98 (0.86-12.04) | 457 (74-926) | 5.15 (0.83-10.45) | -0.56 (-0.64--0.47) |
| Bosnia and Herzegovina | 150 (6-326) | 3.42 (0.14-7.41) | 56 (6-162) | 0.96 (0.09-2.78) | -5.39 (-5.8--4.98) |
| Botswana | 239 (37-489) | 38.52 (6.01-78.83) | 499 (75-1015) | 32.19 (4.88-64.68) | -1.32 (-1.81--0.84) |
| Brazil | 16056 (2431-31084) | 16.75 (2.58-32.43) | 26669 (2384-53397) | 10.9 (0.98-21.84) | -1.56 (-1.62--1.51) |
| Brunei Darussalam | 9 (1-18) | 9.11 (0.81-18.38) | 16 (1-33) | 5.23 (0.5-10.62) | -2.05 (-2.33--1.76) |
| Bulgaria | 208 (17-546) | 1.69 (0.14-4.46) | 41 (12-121) | 0.31 (0.1-0.88) | -7.03 (-7.65--6.39) |
| Burkina Faso | 455 (79-857) | 9.82 (1.7-18.43) | 1286 (199-2530) | 13.04 (2-25.58) | 1.33 (1.14-1.53) |
| Burundi | 1260 (191-2560) | 50.37 (7.79-102.29) | 1680 (286-3517) | 32.28 (5.63-67.04) | -2.03 (-2.22--1.84) |
| Cabo Verde | 49 (8-96) | 22.6 (3.55-43.61) | 89 (4-201) | 20.42 (0.94-46.02) | -1.83 (-2.39--1.26) |
| Cambodia | 489 (77-982) | 9.88 (1.52-19.71) | 943 (151-1840) | 7.42 (1.2-14.45) | -1.15 (-1.26--1.03) |
| Cameroon | 476 (65-952) | 9.87 (1.35-19.75) | 1235 (53-2914) | 9.58 (0.45-22.54) | -0.16 (-0.25--0.07) |
| Canada | 1312 (55-2898) | 4.11 (0.17-9.09) | 2901 (122-6331) | 4.45 (0.19-9.78) | 0.59 (0.4-0.78) |
| Central African Republic | 571 (92-1129) | 42.75 (7.26-84.32) | 828 (138-1682) | 32.63 (5.74-65.78) | -1.06 (-1.16--0.97) |
| Chad | 270 (49-510) | 9.27 (1.69-17.57) | 810 (132-1591) | 13.53 (2.2-26.68) | 1.74 (1.54-1.94) |
| Chile | 1024 (42-2147) | 10.44 (0.43-21.89) | 797 (38-1807) | 3.31 (0.16-7.51) | -4.35 (-4.51--4.19) |
| China | 291820 (14233-634123) | 33.19 (1.65-71.64) | 20176 (10483-43291) | 1.03 (0.51-2.24) | -12.68 (-13.09--12.28) |
| Colombia | 1436 (126-2876) | 8.08 (0.71-16.14) | 1821 (178-3799) | 3.46 (0.34-7.22) | -3.37 (-3.56--3.18) |
| Comoros | 100 (17-206) | 43.38 (7.23-88.74) | 168 (33-328) | 32.87 (6.45-63.67) | -1.3 (-1.51--1.09) |
| Congo | 514 (80-1004) | 43.94 (6.81-84.97) | 822 (133-1608) | 28.16 (4.45-54.71) | -1.85 (-2.04--1.66) |
| Cook Islands | 1 (0-2) | 7.62 (1.24-15) | 1 (0-3) | 5.41 (0.69-10.92) | -1.32 (-1.41--1.23) |
| Costa Rica | 116 (20-217) | 6.56 (1.12-12.32) | 174 (22-351) | 3.37 (0.44-6.82) | -2.76 (-3.05--2.47) |
| Croatia | 355 (14-789) | 5.4 (0.22-12.03) | 304 (13-717) | 3.9 (0.16-9.26) | -1.31 (-1.46--1.16) |
| Cuba | 706 (46-1439) | 6.89 (0.45-14.06) | 1057 (51-2507) | 5.68 (0.28-13.52) | -0.94 (-1.2--0.69) |
| Cyprus | 15 (1-32) | 1.81 (0.07-3.95) | 33 (1-74) | 1.73 (0.08-3.88) | 0.17 (-0.07-0.42) |
| Czechia | 679 (27-1423) | 5.14 (0.21-10.78) | 990 (44-2210) | 5.31 (0.23-11.95) | 0.1 (-0.09-0.28) |
| Côte d'Ivoire | 441 (67-866) | 9.68 (1.48-18.83) | 1422 (200-2968) | 12.04 (1.7-25.31) | 0.96 (0.83-1.1) |
| Democratic People's Republic of Korea | 1378 (85-3800) | 8.05 (0.5-21.98) | 4095 (171-9386) | 12.32 (0.51-28.22) | 1.32 (0.9-1.73) |
| Democratic Republic of the Congo | 5287 (930-10458) | 30.26 (5.41-60.89) | 9489 (1514-19563) | 24.08 (3.96-50.17) | -1 (-1.08--0.91) |
| Denmark | 637 (31-1325) | 8.65 (0.41-18.06) | 511 (24-1160) | 4.75 (0.22-10.86) | -2.38 (-2.54--2.21) |
| Djibouti | 56 (8-120) | 33.73 (4.98-71.33) | 182 (20-401) | 26.19 (2.89-56.37) | -1.25 (-1.45--1.05) |
| Dominica | 6 (0-12) | 8.73 (0.37-18.17) | 7 (0-16) | 8.3 (0.36-17.84) | -0.04 (-0.29-0.21) |
| Dominican Republic | 156 (22-300) | 3.92 (0.57-7.62) | 468 (33-1053) | 4.87 (0.34-10.94) | 1.17 (0.97-1.38) |
| Ecuador | 275 (47-518) | 5.06 (0.88-9.55) | 480 (84-989) | 3.2 (0.56-6.6) | -1.51 (-1.72--1.31) |
| Egypt | 65 (21-182) | 0.22 (0.06-0.64) | 58 (40-79) | 0.08 (0.06-0.12) | -2.77 (-3.57--1.97) |
| El Salvador | 106 (18-202) | 3.51 (0.59-6.67) | 176 (15-379) | 2.99 (0.26-6.45) | -1.11 (-1.44--0.77) |
| Equatorial Guinea | 78 (12-161) | 35.68 (5.5-72.99) | 107 (18-246) | 20.87 (3.46-47.19) | -2.22 (-2.56--1.88) |
| Eritrea | 545 (100-1102) | 45.13 (8.31-92.04) | 1169 (199-2395) | 37.41 (6.22-76.63) | -0.9 (-1.09--0.7) |
| Estonia | 135 (7-280) | 6.6 (0.36-13.7) | 79 (4-189) | 3.48 (0.16-8.47) | -2.7 (-2.92--2.49) |
| Eswatini | 145 (23-293) | 44.65 (7.27-88.96) | 281 (47-564) | 43.96 (7.45-87.77) | -0.19 (-0.87-0.49) |
| Ethiopia | 3100 (549-6409) | 13.62 (2.47-27.75) | 3147 (597-6430) | 7.13 (1.36-14.63) | -2.56 (-2.7--2.42) |
| Fiji | 24 (4-46) | 5.98 (1.07-11.73) | 45 (5-94) | 5.79 (0.69-12.06) | 0.23 (-0.09-0.54) |
| Finland | 372 (29-756) | 5.35 (0.41-10.83) | 405 (18-839) | 3.67 (0.16-7.67) | -1.08 (-1.23--0.93) |
| France | 8675 (326-18724) | 11.74 (0.44-25.43) | 7536 (344-15971) | 6.48 (0.28-13.69) | -2.26 (-2.36--2.15) |
| Gabon | 197 (30-404) | 33.47 (5.13-68.39) | 302 (46-615) | 26.63 (4.13-54.51) | -1.03 (-1.17--0.88) |
| Gambia | 17 (3-33) | 4.48 (0.84-8.71) | 57 (9-112) | 5.64 (0.91-11.15) | 0.67 (0.48-0.86) |
| Georgia | 120 (7-293) | 1.91 (0.11-4.62) | 145 (6-332) | 2.6 (0.11-5.95) | 1.22 (1.06-1.38) |
| Germany | 6636 (290-13907) | 5.74 (0.25-12.06) | 9193 (355-19665) | 5.61 (0.21-12.03) | -0.23 (-0.44--0.01) |
| Ghana | 513 (76-1007) | 7.56 (1.13-14.75) | 1266 (154-2558) | 7.38 (0.91-15) | 0.14 (0-0.28) |
| Greece | 15 (14-21) | 0.11 (0.09-0.15) | 39 (12-114) | 0.16 (0.06-0.43) | 0.93 (0.41-1.45) |
| Greenland | 7 (0-16) | 17.79 (0.86-40.77) | 8 (1-21) | 10.78 (0.67-27.03) | -2.03 (-2.27--1.78) |
| Grenada | 13 (2-24) | 19.52 (3.41-37.08) | 19 (3-37) | 16.15 (2.65-31.23) | -0.43 (-0.56--0.31) |
| Guam | 4 (0-8) | 5.44 (0.6-10.42) | 10 (1-22) | 5.37 (0.5-11.21) | 0.36 (-0.11-0.83) |
| Guatemala | 144 (22-277) | 3.76 (0.58-7.15) | 455 (43-948) | 3.99 (0.38-8.33) | -0.08 (-0.54-0.38) |
| Guinea | 132 (16-262) | 3.82 (0.47-7.65) | 275 (44-544) | 4.73 (0.75-9.42) | 1.03 (0.88-1.18) |
| Guinea-Bissau | 71 (11-142) | 16 (2.56-32.15) | 144 (23-296) | 17.96 (2.85-36.78) | 0.87 (0.68-1.06) |
| Guyana | 25 (4-47) | 6.14 (1.04-11.67) | 31 (3-66) | 4.55 (0.4-9.56) | -1.02 (-1.21--0.84) |
| Haiti | 440 (78-877) | 12.49 (2.19-24.85) | 755 (129-1608) | 9.95 (1.74-21.01) | -0.61 (-0.79--0.44) |
| Honduras | 65 (11-122) | 3 (0.5-5.68) | 210 (24-431) | 3.43 (0.4-7.06) | 0.67 (0.48-0.85) |
| Hungary | 1005 (37-2174) | 7.3 (0.27-15.86) | 440 (32-1211) | 2.59 (0.2-7.14) | -4.7 (-5.11--4.28) |
| Iceland | 26 (3-51) | 9.66 (1.26-18.99) | 34 (2-70) | 6.57 (0.36-13.62) | -1.56 (-1.76--1.37) |
| India | 47827 (5881-95380) | 9.45 (1.19-18.89) | 83426 (7353-174580) | 6.9 (0.61-14.41) | -1.29 (-1.42--1.15) |
| Indonesia | 7180 (1170-13696) | 6.56 (1.08-12.64) | 11517 (1321-23703) | 4.98 (0.58-10.16) | -0.91 (-0.96--0.87) |
| Iran (Islamic Republic of) | 1956 (90-4416) | 7.2 (0.34-16.26) | 900 (147-2306) | 1.28 (0.2-3.27) | -6.89 (-7.42--6.35) |
| Iraq | 65 (5-179) | 0.8 (0.06-2.23) | 148 (15-425) | 0.62 (0.06-1.74) | -2.72 (-3.34--2.09) |
| Ireland | 463 (23-960) | 11.5 (0.56-23.78) | 457 (21-1075) | 6.23 (0.28-14.66) | -2.27 (-2.42--2.11) |
| Israel | 16 (4-45) | 0.33 (0.08-0.93) | 12 (7-28) | 0.1 (0.06-0.23) | -3.74 (-4.59--2.87) |
| Italy | 962 (133-2408) | 1.12 (0.16-2.8) | 2006 (112-4564) | 1.58 (0.09-3.59) | 1.74 (1.49-1.98) |
| Jamaica | 101 (8-201) | 5.76 (0.46-11.54) | 133 (8-291) | 4.51 (0.26-9.87) | -0.96 (-1.3--0.62) |
| Japan | 6838 (414-15713) | 3.94 (0.24-9.05) | 4108 (530-10762) | 1.4 (0.18-3.65) | -3.11 (-3.49--2.73) |
| Jordan | 26 (1-58) | 1.8 (0.07-4.05) | 91 (4-213) | 1.28 (0.06-3.02) | -1.79 (-2.04--1.54) |
| Kazakhstan | 3351 (138-7537) | 26.02 (1.07-58.2) | 166 (59-451) | 1.06 (0.34-2.82) | -12.82 (-13.93--11.69) |
| Kenya | 2121 (303-4376) | 23.99 (3.51-49.57) | 6551 (693-13861) | 27.3 (3.07-58.06) | 0.83 (0.6-1.07) |
| Kiribati | 9 (1-18) | 21.56 (3.5-43.56) | 15 (2-32) | 19.66 (3.04-40.94) | -0.48 (-0.61--0.34) |
| Kuwait | 5 (1-13) | 0.75 (0.08-2.03) | 7 (1-19) | 0.29 (0.05-0.82) | -3.58 (-5.33--1.81) |
| Kyrgyzstan | 208 (13-520) | 6.74 (0.43-16.73) | 61 (9-184) | 1.4 (0.19-4.12) | -7.18 (-7.88--6.48) |
| Lao People's Democratic Republic | 267 (43-524) | 11.66 (1.91-22.6) | 228 (16-490) | 4.79 (0.35-10.35) | -3.56 (-3.77--3.35) |
| Latvia | 142 (6-320) | 4 (0.17-9.01) | 133 (6-321) | 3.93 (0.19-9.53) | -0.52 (-0.9--0.13) |
| Lebanon | 2 (1-2) | 0.07 (0.05-0.1) | 4 (2-8) | 0.08 (0.05-0.15) | 0.41 (0.08-0.75) |
| Lesotho | 346 (60-676) | 33.25 (5.82-64.66) | 583 (86-1160) | 42.23 (6.34-84.34) | 1.2 (0.93-1.47) |
| Liberia | 117 (20-226) | 10.15 (1.77-19.63) | 294 (46-587) | 13.08 (2.06-25.87) | 1.37 (1.06-1.69) |
| Libya | 18 (1-48) | 0.93 (0.06-2.5) | 86 (4-207) | 1.56 (0.07-3.72) | 1.39 (0.93-1.86) |
| Lithuania | 240 (9-511) | 5.34 (0.2-11.39) | 206 (10-506) | 4.2 (0.21-10.4) | -1.01 (-1.33--0.69) |
| Luxembourg | 43 (2-89) | 8.2 (0.32-17.03) | 38 (2-87) | 3.96 (0.18-9.13) | -2.72 (-2.85--2.6) |
| Madagascar | 1978 (362-3978) | 34.98 (6.53-70.02) | 3631 (733-7107) | 28.39 (5.74-54.67) | -0.94 (-1.11--0.77) |
| Malawi | 2667 (531-5023) | 62.06 (12.19-117.7) | 5484 (1087-11043) | 68.37 (13.74-137.71) | 0.36 (0.01-0.71) |
| Malaysia | 655 (105-1261) | 6.84 (1.1-13.03) | 1300 (102-2745) | 4.74 (0.39-10.01) | -1.7 (-1.9--1.49) |
| Maldives | 9 (1-21) | 9.2 (0.85-20.06) | 9 (0-20) | 2.86 (0.14-6.19) | -4.85 (-5.17--4.53) |
| Mali | 319 (57-591) | 7.1 (1.26-13.28) | 634 (108-1229) | 6.8 (1.15-13.08) | -0.16 (-0.23--0.08) |
| Malta | 14 (1-31) | 3.24 (0.14-7.3) | 6 (1-18) | 0.73 (0.1-2.08) | -5.04 (-5.34--4.73) |
| Marshall Islands | 1 (0-3) | 8.14 (1.47-16.11) | 3 (0-6) | 7.08 (1.18-14.24) | -0.47 (-0.55--0.4) |
| Mauritania | 130 (22-246) | 12.48 (2.1-23.6) | 226 (34-447) | 10.54 (1.6-20.66) | -0.17 (-0.39-0.05) |
| Mauritius | 54 (6-106) | 7.02 (0.82-13.79) | 75 (4-161) | 4.18 (0.22-9.01) | -1.87 (-2.07--1.68) |
| Mexico | 1679 (165-3323) | 3.81 (0.38-7.56) | 3050 (218-6247) | 2.55 (0.18-5.21) | -1.48 (-1.72--1.24) |
| Micronesia (Federated States of) | 5 (1-10) | 9.36 (1.63-18.68) | 6 (1-13) | 8.08 (1.25-16.93) | -0.68 (-0.74--0.63) |
| Monaco | 1 (0-2) | 1.13 (0.22-3.31) | 1 (0-2) | 0.58 (0.21-1.65) | -1.57 (-1.92--1.22) |
| Mongolia | 638 (119-1225) | 62.1 (11.67-119.58) | 947 (103-1952) | 42.97 (4.95-86.84) | -1.84 (-2.13--1.54) |
| Montenegro | 2 (1-5) | 0.28 (0.1-0.74) | 2 (1-3) | 0.18 (0.11-0.34) | -6.15 (-7.83--4.44) |
| Morocco | 357 (14-779) | 2.48 (0.1-5.43) | 326 (25-871) | 1.03 (0.08-2.71) | -3.8 (-4.32--3.28) |
| Mozambique | 1157 (215-2193) | 18.25 (3.38-34.58) | 2515 (418-4871) | 21.49 (3.59-41.23) | 0.89 (0.77-1.02) |
| Myanmar | 2372 (376-5012) | 9.31 (1.51-19.53) | 2101 (124-4483) | 4.28 (0.26-8.99) | -3.06 (-3.2--2.92) |
| Namibia | 55 (11-107) | 7.34 (1.42-14.28) | 103 (16-212) | 6.81 (1.08-13.8) | -0.63 (-0.95--0.31) |
| Nauru | 0 (0-1) | 8.93 (1.38-17.44) | 0 (0-1) | 7.75 (1.19-15.62) | -0.79 (-1.18--0.39) |
| Nepal | 1490 (238-2904) | 14.05 (2.32-27.38) | 2333 (226-4698) | 10.05 (0.99-20.34) | -1.17 (-1.54--0.79) |
| Netherlands | 1359 (65-2834) | 7.12 (0.34-14.85) | 3359 (158-6993) | 10.49 (0.48-21.9) | 1.43 (1.05-1.82) |
| New Zealand | 222 (9-478) | 5.68 (0.23-12.21) | 350 (15-755) | 4.64 (0.2-10) | -0.58 (-0.67--0.49) |
| Nicaragua | 48 (9-90) | 3.06 (0.55-5.71) | 130 (22-250) | 2.93 (0.49-5.66) | -0.74 (-0.96--0.51) |
| Niger | 236 (27-464) | 7.68 (0.89-15) | 627 (42-1366) | 7.58 (0.55-16.32) | 0.25 (0.02-0.49) |
| Nigeria | 915 (110-2232) | 1.98 (0.24-4.79) | 1488 (94-3901) | 1.65 (0.11-4.15) | -0.7 (-0.82--0.58) |
| Niue | 0 (0-0) | 6.37 (1.08-12.61) | 0 (0-0) | 5.58 (0.92-10.94) | -0.75 (-0.9--0.6) |
| North Macedonia | 4 (1-12) | 0.24 (0.08-0.66) | 5 (2-15) | 0.17 (0.07-0.46) | -2.92 (-4.02--1.8) |
| Northern Mariana Islands | 1 (0-2) | 3.27 (0.36-7.62) | 3 (0-7) | 6.02 (0.79-12.28) | 3.14 (2.7-3.57) |
| Norway | 290 (25-584) | 4.73 (0.41-9.58) | 348 (23-723) | 3.88 (0.24-8.05) | -0.52 (-0.61--0.42) |
| Oman | 20 (1-48) | 2.8 (0.12-6.55) | 39 (2-96) | 2.08 (0.11-4.98) | -1 (-1.1--0.9) |
| Pakistan | 11798 (2164-22460) | 19.57 (3.61-37.38) | 25485 (3973-51293) | 20.39 (3.17-40.44) | 0 (-0.2-0.21) |
| Palau | 1 (0-1) | 5.73 (0.96-11.71) | 1 (0-3) | 5.64 (0.81-11.28) | -0.18 (-0.29--0.07) |
| Palestine | 32 (6-64) | 3.51 (0.62-7.03) | 28 (2-66) | 1.14 (0.07-2.73) | -5.47 (-6.26--4.68) |
| Panama | 61 (10-115) | 4 (0.67-7.62) | 146 (23-297) | 3.51 (0.55-7.16) | -0.09 (-0.34-0.17) |
| Papua New Guinea | 88 (11-193) | 4.32 (0.55-9.47) | 231 (31-505) | 4.37 (0.63-9.45) | 0.07 (-0.01-0.15) |
| Paraguay | 163 (25-317) | 7.26 (1.11-14.08) | 476 (62-986) | 8.39 (1.11-17.34) | 0.36 (0.22-0.5) |
| Peru | 485 (68-945) | 3.96 (0.57-7.67) | 779 (80-1644) | 2.42 (0.25-5.13) | -1.72 (-1.86--1.57) |
| Philippines | 1263 (182-2437) | 3.77 (0.55-7.28) | 2352 (200-4817) | 2.75 (0.24-5.6) | -1.37 (-1.59--1.15) |
| Poland | 1360 (87-3176) | 3.15 (0.2-7.34) | 788 (107-2051) | 1.2 (0.17-3.11) | -3.43 (-3.69--3.17) |
| Portugal | 369 (32-972) | 2.76 (0.24-7.23) | 262 (33-741) | 1.26 (0.17-3.64) | -1.45 (-1.96--0.93) |
| Puerto Rico | 577 (94-1078) | 16.01 (2.61-29.85) | 364 (62-723) | 5.64 (0.95-11.26) | -4.11 (-4.38--3.84) |
| Qatar | 1 (0-3) | 0.9 (0.18-2.73) | 2 (1-5) | 0.31 (0.13-0.99) | -5.42 (-6.04--4.8) |
| Republic of Korea | 245 (93-716) | 0.92 (0.29-2.49) | 915 (98-2547) | 1.02 (0.11-2.83) | 0.53 (0.28-0.78) |
| Republic of Moldova | 115 (7-284) | 2.51 (0.15-6.13) | 168 (7-371) | 2.95 (0.13-6.52) | 0.26 (-0.06-0.58) |
| Romania | 162 (24-472) | 0.59 (0.09-1.7) | 49 (38-66) | 0.15 (0.12-0.2) | -6.43 (-7.72--5.11) |
| Russian Federation | 10071 (516-22207) | 5.44 (0.28-11.99) | 10501 (516-23632) | 4.56 (0.22-10.21) | -0.03 (-0.72-0.66) |
| Rwanda | 1827 (315-3587) | 57.29 (9.74-111.4) | 1819 (324-3713) | 27.47 (5.01-55.97) | -3.63 (-4.03--3.22) |
| Saint Kitts and Nevis | 4 (1-8) | 12.27 (1.77-23.63) | 7 (1-13) | 8.97 (0.89-17.74) | -1.34 (-1.73--0.96) |
| Saint Lucia | 12 (2-22) | 13.69 (2.44-25.7) | 23 (4-45) | 10.59 (1.77-20.18) | -0.95 (-1.28--0.62) |
| Saint Vincent and the Grenadines | 4 (1-8) | 6.09 (0.99-11.54) | 8 (1-15) | 5.63 (0.76-10.97) | -0.35 (-0.71-0) |
| Samoa | 5 (1-10) | 6.01 (1.22-11.26) | 7 (1-12) | 4.35 (0.85-8.19) | -1.38 (-1.47--1.29) |
| San Marino | 1 (0-1) | 1.73 (0.08-4.1) | 1 (0-2) | 1.58 (0.07-4.22) | -0.5 (-0.97--0.02) |
| Sao Tome and Principe | 5 (1-10) | 7.84 (1.44-15.39) | 14 (2-28) | 12.15 (2-24.3) | 1.82 (1.72-1.93) |
| Saudi Arabia | 138 (6-335) | 2.23 (0.1-5.37) | 495 (20-1147) | 2.28 (0.1-5.2) | -0.15 (-0.5-0.2) |
| Senegal | 316 (52-616) | 9.28 (1.54-18.04) | 801 (83-1643) | 10.21 (1.09-20.88) | 0.92 (0.47-1.37) |
| Serbia | 401 (17-893) | 3.33 (0.14-7.34) | 424 (19-1026) | 2.91 (0.13-7.07) | -1.01 (-1.31--0.7) |
| Seychelles | 8 (1-16) | 15.24 (2.55-28.74) | 16 (3-31) | 13.53 (2.19-25.84) | -0.65 (-0.78--0.52) |
| Sierra Leone | 166 (27-320) | 8.45 (1.38-16.28) | 404 (61-817) | 10.6 (1.6-21.72) | 1.33 (1.1-1.56) |
| Singapore | 143 (6-315) | 6.47 (0.27-14.15) | 90 (6-234) | 1.15 (0.08-2.95) | -6 (-6.26--5.74) |
| Slovakia | 367 (14-788) | 6.32 (0.24-13.64) | 353 (17-865) | 4.02 (0.19-9.82) | -1.76 (-2.07--1.45) |
| Slovenia | 163 (7-368) | 6.65 (0.28-15.02) | 150 (6-339) | 3.92 (0.16-8.95) | -2.34 (-2.53--2.15) |
| Solomon Islands | 17 (3-35) | 10.88 (1.87-21.69) | 38 (6-77) | 10.4 (1.79-20.43) | -0.21 (-0.34--0.09) |
| Somalia | 1624 (295-3219) | 53.51 (9.77-106.92) | 3002 (528-6073) | 39.35 (6.97-79.8) | -1.13 (-1.2--1.05) |
| South Africa | 7543 (957-15143) | 33.06 (4.24-65.93) | 10138 (1249-20258) | 21.63 (2.7-42.88) | -2.28 (-2.94--1.61) |
| South Sudan | 934 (144-1900) | 36.91 (5.79-74.27) | 1112 (177-2307) | 26 (4.16-53.57) | -1.43 (-1.5--1.35) |
| Spain | 555 (104-1586) | 1.09 (0.21-3.13) | 1912 (108-4682) | 2.28 (0.13-5.64) | 3.12 (2.55-3.7) |
| Sri Lanka | 1387 (236-2655) | 11.99 (2.01-23.03) | 2316 (247-4875) | 8.86 (0.94-18.63) | -0.64 (-1.07--0.2) |
| Sudan | 845 (57-1937) | 8.6 (0.6-19.83) | 1575 (80-3843) | 8.01 (0.42-19.67) | -0.37 (-0.48--0.25) |
| Suriname | 9 (1-17) | 3.16 (0.4-6.17) | 20 (2-40) | 3.11 (0.35-6.29) | -0.09 (-0.37-0.2) |
| Sweden | 634 (54-1267) | 4.54 (0.38-9.09) | 735 (35-1540) | 3.82 (0.18-8.01) | -0.68 (-0.82--0.53) |
| Switzerland | 740 (31-1545) | 7.74 (0.32-16.2) | 749 (31-1602) | 4.7 (0.19-10.12) | -1.55 (-1.65--1.46) |
| Syrian Arab Republic | 44 (3-117) | 0.8 (0.05-2.09) | 180 (8-419) | 1.38 (0.06-3.14) | 1.07 (0.72-1.43) |
| Taiwan (Province of China) | 1438 (56-3058) | 8.44 (0.34-17.91) | 2143 (169-5899) | 5.65 (0.45-15.65) | -0.98 (-1.21--0.76) |
| Tajikistan | 225 (19-584) | 7.86 (0.66-20.23) | 128 (16-427) | 2.74 (0.3-8.9) | -5.14 (-6.73--3.53) |
| Thailand | 3895 (599-7773) | 9.95 (1.56-19.89) | 6992 (747-15118) | 6.68 (0.72-14.4) | -1.95 (-2.22--1.68) |
| Timor-Leste | 24 (5-48) | 7.29 (1.36-14.22) | 51 (9-104) | 6.05 (1.1-12.26) | -0.74 (-1.02--0.45) |
| Togo | 132 (25-252) | 9.73 (1.82-18.58) | 528 (82-1051) | 12.91 (2.02-25.64) | 1.37 (1.21-1.53) |
| Tokelau | 0 (0-0) | 5.8 (0.96-11.62) | 0 (0-0) | 4.49 (0.79-9.01) | -1 (-1.04--0.96) |
| Tonga | 3 (1-6) | 5.23 (0.98-10.74) | 4 (1-8) | 5.1 (0.92-9.83) | -0.35 (-0.67--0.04) |
| Trinidad and Tobago | 51 (8-96) | 5.85 (0.92-11.17) | 67 (9-138) | 3.61 (0.5-7.43) | -2.35 (-2.7--1.99) |
| Tunisia | 54 (2-124) | 1.05 (0.05-2.39) | 35 (5-105) | 0.29 (0.04-0.85) | -5.65 (-6.1--5.19) |
| Turkey | 49 (34-66) | 0.13 (0.09-0.18) | 70 (53-88) | 0.08 (0.06-0.1) | -1.73 (-1.95--1.52) |
| Turkmenistan | 543 (35-1355) | 27.51 (1.71-68.08) | 28 (18-52) | 0.71 (0.42-1.42) | -14.49 (-15.61--13.36) |
| Tuvalu | 1 (0-1) | 7.94 (1.42-15.88) | 1 (0-1) | 5.98 (1.04-11.79) | -1.13 (-1.21--1.05) |
| Uganda | 2552 (501-4803) | 36.85 (7.24-69.11) | 6642 (1152-13260) | 42.65 (7.42-84) | 0.39 (0.2-0.58) |
| Ukraine | 2480 (144-5896) | 3.49 (0.2-8.38) | 819 (112-2494) | 1.16 (0.16-3.57) | -6.7 (-7.8--5.59) |
| United Arab Emirates | 2 (1-3) | 0.5 (0.15-1.07) | 599 (23-1685) | 9.18 (0.35-27.15) | 13.07 (11.41-14.75) |
| United Kingdom | 9420 (634-19558) | 10.93 (0.73-22.69) | 11804 (725-24644) | 10.03 (0.6-21.01) | -0.52 (-0.63--0.42) |
| United Republic of Tanzania | 4701 (854-9446) | 39.93 (7.35-79.97) | 7890 (1283-16763) | 29.59 (4.98-61.66) | -1.24 (-1.34--1.14) |
| United States of America | 12385 (625-27306) | 4.14 (0.21-9.15) | 25618 (1988-52818) | 4.81 (0.37-9.92) | 0.88 (0.69-1.08) |
| United States Virgin Islands | 7 (1-14) | 7.21 (0.74-14.81) | 14 (1-28) | 7.59 (0.5-15.71) | 0.62 (0.29-0.96) |
| Uruguay | 801 (127-1549) | 20.97 (3.31-40.66) | 583 (55-1164) | 11.57 (1.1-23.09) | -2.26 (-2.39--2.13) |
| Uzbekistan | 691 (100-1969) | 6.08 (0.85-17.02) | 79 (65-94) | 0.37 (0.29-0.52) | -13.17 (-14.74--11.56) |
| Vanuatu | 5 (1-10) | 6.52 (1.01-13.42) | 12 (2-25) | 6.58 (1.11-13.44) | -0.23 (-0.38--0.09) |
| Venezuela (Bolivarian Republic of) | 572 (94-1104) | 5.77 (0.95-11.15) | 1153 (99-2444) | 3.87 (0.34-8.19) | -1.66 (-1.9--1.41) |
| Viet Nam | 2376 (368-4752) | 5.74 (0.89-11.35) | 3617 (180-8796) | 3.56 (0.17-8.46) | -2 (-2.41--1.59) |
| Yemen | 551 (62-1224) | 10.33 (1.16-22.46) | 1445 (171-3155) | 10.03 (1.21-21.64) | -0.22 (-0.28--0.16) |
| Zambia | 1576 (255-3055) | 49.43 (8.06-94.9) | 3028 (484-5913) | 39.64 (6.33-77.07) | -1.23 (-1.54--0.92) |
| Zimbabwe | 1881 (371-3437) | 43.17 (8.47-79.4) | 3543 (635-6689) | 45.9 (8.25-85.88) | 0.2 (-0.06-0.46) |

**Table S5.** The number of deaths cases and the age-standardized deaths rate of esophageal cancer attributable to diet low in fruits in 1990 and 2019, and its trends from 1990 to 2019 globally.

|  | Number of deaths cases (95% UI) in 1990 | The age-standardized deaths rate/100000 (95% UI) in 1990 | Number of deaths cases (95% UI) in 2019 | The age-standardized deaths rate/100000 (95% UI) in 2019 | EAPC (95% CI) |
| --- | --- | --- | --- | --- | --- |
| Global | 51867 (17816-92689) | 1.32 (0.45-2.37) | 51210 (15227-108734) | 0.63 (0.19-1.33) | -2.96 (-3.27--2.65) |
| **Sex** |  |  |  |  |  |
| Female | 18006 (6627-32143) | 0.86 (0.31-1.53) | 15509 (5185-30419) | 0.35 (0.12-0.7) | -3.54 (-3.88--3.19) |
| Male | 33861 (11023-61135) | 1.86 (0.6-3.39) | 35701 (10018-78761) | 0.94 (0.26-2.09) | -2.68 (-2.98--2.38) |
| **Age** |  |  |  |  |  |
| <24 years | 0 (0-0) | 0 (0-0) | 0 (0-0) | 0 (0-0) | 0 (0-0) |
| 25-29 years | 143 (65-226) | 0.03 (0.01-0.05) | 127 (53-204) | 0.02 (0.01-0.03) | -2.18 (-2.52--1.85) |
| 30-34 years | 296 (127-458) | 0.08 (0.03-0.12) | 279 (110-470) | 0.05 (0.02-0.08) | -2.38 (-2.75--2.01) |
| 35-39 years | 744 (297-1170) | 0.21 (0.08-0.33) | 589 (228-1002) | 0.11 (0.04-0.19) | -2.65 (-3.01--2.29) |
| 40-44 years | 1661 (634-2783) | 0.58 (0.22-0.97) | 1299 (472-2294) | 0.26 (0.1-0.46) | -3.33 (-3.64--3.02) |
| 45-49 years | 2592 (949-4366) | 1.12 (0.41-1.88) | 2493 (875-4700) | 0.53 (0.18-0.99) | -3.16 (-3.54--2.77) |
| 50-54 years | 4570 (1650-7975) | 2.15 (0.78-3.75) | 4244 (1403-8555) | 0.97 (0.32-1.96) | -3.25 (-3.69--2.81) |
| 55-59 years | 6590 (2210-11826) | 3.55 (1.19-6.38) | 5635 (1747-11586) | 1.52 (0.47-3.12) | -3.21 (-3.48--2.94) |
| 60-64 years | 7954 (2539-14578) | 4.95 (1.58-9.07) | 6384 (1964-13979) | 2.04 (0.63-4.47) | -3.31 (-3.53--3.08) |
| 65-69 years | 8395 (2637-15441) | 6.8 (2.14-12.5) | 7359 (2014-16744) | 2.85 (0.78-6.48) | -3.39 (-3.68--3.11) |
| 70-74 years | 7781 (2462-14414) | 9.21 (2.91-17.06) | 7336 (1958-16608) | 3.92 (1.05-8.88) | -3.38 (-3.72--3.03) |
| 75-79 years | 5894 (1985-10718) | 9.61 (3.24-17.48) | 6017 (1620-13350) | 4.74 (1.28-10.51) | -3.06 (-3.44--2.67) |
| 80-84 years | 3432 (1191-6184) | 9.74 (3.38-17.56) | 5019 (1337-11208) | 5.94 (1.58-13.28) | -1.99 (-2.39--1.59) |
| 85-89 years | 1404 (440-2679) | 9.32 (2.92-17.78) | 3155 (817-6996) | 7.26 (1.88-16.09) | -1.05 (-1.39--0.71) |
| 90-94 years | 341 (114-652) | 7.74 (2.6-14.8) | 1012 (273-2207) | 6 (1.62-13.09) | -1.03 (-1.25--0.8) |
| 95+ years | 70 (23-137) | 6.8 (2.2-13.26) | 264 (71-588) | 5.53 (1.48-12.31) | -0.64 (-0.71--0.58) |
| **SDI regions** |  |  |  |  |  |
| High SDI | 5081 (1156-11701) | 0.49 (0.11-1.13) | 6990 (1411-17264) | 0.37 (0.08-0.91) | -1.05 (-1.12--0.99) |
| High-middle SDI | 12968 (3881-24354) | 1.22 (0.37-2.3) | 10205 (2269-25842) | 0.5 (0.11-1.26) | -3.6 (-3.98--3.22) |
| Middle SDI | 24518 (8095-43210) | 2.5 (0.83-4.4) | 17982 (4599-41528) | 0.76 (0.2-1.76) | -4.52 (-5.02--4.03) |
| Low-middle SDI | 6888 (3103-10731) | 1.18 (0.53-1.85) | 11400 (4969-19129) | 0.85 (0.37-1.42) | -1.38 (-1.5--1.26) |
| Low SDI | 2404 (938-4126) | 1.04 (0.4-1.78) | 4621 (1747-7820) | 0.91 (0.34-1.55) | -0.58 (-0.69--0.47) |
| **GBD regions** |  |  |  |  |  |
| Africa | 2901 (1092-5075) | 1.05 (0.39-1.83) | 5226 (1931-9214) | 0.86 (0.32-1.53) | -0.97 (-1.21--0.72) |
| African Region | 2709 (1041-4634) | 1.25 (0.48-2.15) | 4878 (1790-8561) | 1.04 (0.39-1.82) | -0.96 (-1.22--0.69) |
| America | 2077 (321-5575) | 0.34 (0.05-0.92) | 2905 (416-8484) | 0.23 (0.03-0.66) | -1.53 (-1.66--1.39) |
| Andean Latin America | 34 (7-82) | 0.18 (0.04-0.42) | 54 (7-160) | 0.1 (0.01-0.29) | -2.04 (-2.13--1.95) |
| Asia | 41986 (15008-71845) | 2.17 (0.77-3.72) | 38236 (11828-80155) | 0.82 (0.25-1.71) | -3.76 (-4.13--3.38) |
| Australasia | 108 (21-259) | 0.46 (0.09-1.1) | 182 (26-487) | 0.36 (0.05-0.96) | -1.02 (-1.08--0.96) |
| Caribbean | 74 (10-210) | 0.29 (0.04-0.83) | 98 (13-289) | 0.19 (0.03-0.56) | -1.68 (-2--1.36) |
| Central Asia | 1150 (405-1982) | 2.54 (0.9-4.35) | 514 (137-1180) | 0.76 (0.2-1.72) | -4.91 (-5.16--4.65) |
| Central Europe | 487 (117-1109) | 0.34 (0.08-0.76) | 554 (106-1352) | 0.27 (0.05-0.66) | -1.02 (-1.23--0.81) |
| Central Latin America | 136 (18-399) | 0.17 (0.02-0.51) | 240 (34-706) | 0.1 (0.01-0.31) | -1.97 (-2.1--1.84) |
| Central Sub-Saharan Africa | 296 (63-708) | 1.35 (0.29-3.21) | 695 (196-1397) | 1.37 (0.39-2.75) | -0.3 (-0.69-0.08) |
| Commonwealth High Income | 1187 (358-2380) | 0.78 (0.23-1.56) | 1580 (394-3509) | 0.6 (0.15-1.32) | -1.18 (-1.28--1.07) |
| Commonwealth Low Income | 1453 (601-2340) | 1.7 (0.7-2.74) | 2372 (804-4346) | 1.11 (0.38-2.02) | -1.53 (-1.61--1.45) |
| Commonwealth Middle Income | 7071 (3446-10422) | 1.19 (0.57-1.77) | 14199 (6407-22079) | 0.97 (0.44-1.51) | -1.03 (-1.23--0.83) |
| East Asia | 31106 (9699-56128) | 3.74 (1.17-6.74) | 19948 (3345-52997) | 1 (0.17-2.63) | -4.95 (-5.51--4.38) |
| East Asia & Pacific - WB | 33773 (10436-60991) | 2.59 (0.8-4.68) | 23512 (4348-60600) | 0.76 (0.14-1.95) | -4.59 (-5.09--4.09) |
| Eastern Europe | 2012 (645-3627) | 0.71 (0.23-1.28) | 1316 (328-2855) | 0.39 (0.1-0.84) | -2.86 (-3.13--2.59) |
| Eastern Mediterranean Region | 1113 (357-2108) | 0.63 (0.2-1.19) | 2364 (795-4347) | 0.55 (0.18-1.02) | -0.52 (-0.66--0.38) |
| Eastern Sub-Saharan Africa | 1342 (525-2302) | 1.82 (0.71-3.14) | 2266 (802-4129) | 1.42 (0.5-2.62) | -1.03 (-1.11--0.95) |
| Europe | 4872 (1323-10331) | 0.47 (0.13-1) | 4822 (1054-11534) | 0.32 (0.07-0.76) | -1.82 (-1.97--1.68) |
| Europe & Central Asia - WB | 5890 (1706-12073) | 0.56 (0.16-1.14) | 5159 (1121-12318) | 0.33 (0.07-0.79) | -2.28 (-2.43--2.12) |
| European Region | 5894 (1706-12078) | 0.55 (0.16-1.13) | 5165 (1121-12342) | 0.33 (0.07-0.78) | -2.28 (-2.44--2.13) |
| High-income Asia Pacific | 1319 (356-2770) | 0.66 (0.18-1.38) | 1794 (437-4019) | 0.4 (0.1-0.88) | -1.59 (-1.8--1.38) |
| High-income North America | 1144 (189-2962) | 0.33 (0.06-0.86) | 1823 (278-4987) | 0.29 (0.04-0.8) | -0.42 (-0.53--0.3) |
| Latin America & Caribbean - WB | 956 (137-2678) | 0.37 (0.05-1.04) | 1091 (138-3403) | 0.17 (0.02-0.51) | -3.11 (-3.28--2.93) |
| Middle East & North Africa - WB | 159 (29-425) | 0.13 (0.02-0.36) | 309 (64-805) | 0.1 (0.02-0.26) | -1.34 (-1.58--1.09) |
| North Africa and Middle East | 310 (67-761) | 0.19 (0.04-0.45) | 527 (110-1351) | 0.12 (0.03-0.32) | -1.8 (-1.96--1.65) |
| North America | 1144 (189-2961) | 0.33 (0.06-0.86) | 1823 (278-4987) | 0.29 (0.04-0.8) | -0.42 (-0.53--0.3) |
| Oceania | 10 (3-20) | 0.38 (0.12-0.72) | 22 (6-44) | 0.33 (0.09-0.68) | -0.36 (-0.4--0.31) |
| Region of the Americas | 2077 (321-5575) | 0.34 (0.05-0.92) | 2905 (416-8484) | 0.23 (0.03-0.66) | -1.53 (-1.66--1.39) |
| South-East Asia Region | 7282 (3562-10812) | 1.08 (0.53-1.6) | 13610 (6145-21695) | 0.81 (0.36-1.29) | -1.25 (-1.37--1.13) |
| South Asia | 6910 (3371-10207) | 1.27 (0.61-1.87) | 13797 (6217-21505) | 1 (0.45-1.56) | -1.06 (-1.2--0.93) |
| South Asia - WB | 7101 (3460-10478) | 1.26 (0.61-1.87) | 14147 (6347-22042) | 1 (0.45-1.55) | -1.04 (-1.18--0.9) |
| Southeast Asia | 1261 (479-2162) | 0.5 (0.19-0.86) | 1664 (425-3565) | 0.28 (0.07-0.6) | -2.07 (-2.11--2.04) |
| Southern Latin America | 290 (40-802) | 0.65 (0.09-1.78) | 185 (21-594) | 0.22 (0.02-0.7) | -4.32 (-4.57--4.06) |
| Southern Sub-Saharan Africa | 844 (358-1354) | 3.1 (1.32-4.96) | 1349 (567-2103) | 2.49 (1.03-3.88) | -1.3 (-1.85--0.75) |
| Sub-Saharan Africa - WB | 2834 (1084-4873) | 1.31 (0.5-2.25) | 5156 (1916-9025) | 1.12 (0.42-1.95) | -0.83 (-1.09--0.58) |
| Tropical Latin America | 425 (63-1201) | 0.47 (0.07-1.32) | 518 (61-1676) | 0.21 (0.03-0.7) | -2.97 (-3.13--2.82) |
| Western Europe | 2306 (527-5516) | 0.41 (0.09-0.98) | 2893 (592-7133) | 0.32 (0.06-0.79) | -1.03 (-1.11--0.94) |
| Western Pacific Region | 32721 (10106-58998) | 2.88 (0.89-5.22) | 22191 (4019-57832) | 0.82 (0.15-2.14) | -4.71 (-5.23--4.18) |
| Western Sub-Saharan Africa | 304 (121-539) | 0.36 (0.14-0.63) | 773 (294-1391) | 0.43 (0.16-0.78) | 0.97 (0.85-1.09) |
| World Bank High Income | 5508 (1245-12745) | 0.43 (0.1-1) | 7434 (1491-18521) | 0.33 (0.07-0.82) | -1.03 (-1.1--0.97) |
| World Bank Low Income | 1969 (704-3609) | 1.35 (0.48-2.48) | 3592 (1212-6586) | 1.18 (0.4-2.17) | -0.6 (-0.71--0.49) |
| World Bank Lower Middle Income | 9478 (4403-14348) | 0.93 (0.43-1.41) | 17202 (7745-26729) | 0.74 (0.33-1.15) | -1.04 (-1.15--0.93) |
| World Bank Upper Middle Income | 34904 (10773-63067) | 2.37 (0.73-4.29) | 22971 (4185-59597) | 0.7 (0.13-1.8) | -4.67 (-5.19--4.15) |
| **Countries** |  |  |  |  |  |
| Afghanistan | 95 (22-194) | 1.36 (0.32-2.75) | 145 (32-295) | 1.19 (0.28-2.4) | -0.74 (-1.1--0.39) |
| Albania | 4 (1-11) | 0.21 (0.03-0.56) | 1 (0-4) | 0.03 (0-0.11) | -7.99 (-8.6--7.37) |
| Algeria | 18 (4-44) | 0.17 (0.03-0.41) | 15 (1-51) | 0.05 (0-0.17) | -4.97 (-5.3--4.64) |
| American Samoa | 0 (0-0) | 0.11 (0.02-0.29) | 0 (0-0) | 0.14 (0.02-0.35) | 0.74 (0.56-0.92) |
| Andorra | 0 (0-0) | 0.21 (0.02-0.69) | 0 (0-1) | 0.17 (0.01-0.54) | -1.14 (-1.29--0.99) |
| Angola | 88 (27-163) | 2.28 (0.73-4.23) | 98 (18-256) | 0.95 (0.18-2.39) | -3.97 (-4.37--3.57) |
| Antigua and Barbuda | 0 (0-0) | 0.14 (0.01-0.52) | 0 (0-1) | 0.14 (0.01-0.51) | 0.46 (0.23-0.7) |
| Argentina | 160 (11-508) | 0.51 (0.04-1.61) | 81 (7-312) | 0.15 (0.01-0.57) | -4.9 (-5.29--4.51) |
| Armenia | 5 (0-15) | 0.2 (0.02-0.57) | 2 (0-8) | 0.05 (0-0.19) | -5.54 (-5.91--5.17) |
| Australia | 96 (20-222) | 0.49 (0.1-1.13) | 162 (24-428) | 0.38 (0.06-1) | -1.07 (-1.14--1.01) |
| Austria | 20 (1-67) | 0.17 (0.01-0.59) | 14 (1-55) | 0.08 (0.01-0.33) | -3.15 (-3.38--2.92) |
| Azerbaijan | 40 (4-117) | 0.81 (0.08-2.35) | 37 (2-140) | 0.45 (0.03-1.68) | -3.1 (-3.6--2.6) |
| Bahamas | 1 (0-2) | 0.56 (0.08-1.51) | 2 (0-4) | 0.4 (0.06-1.08) | -1.58 (-1.72--1.44) |
| Bahrain | 0 (0-1) | 0.12 (0.01-0.44) | 0 (0-1) | 0.04 (0-0.15) | -4.28 (-4.59--3.97) |
| Bangladesh | 780 (365-1174) | 1.69 (0.79-2.55) | 1190 (433-2070) | 0.93 (0.34-1.63) | -2.03 (-2.17--1.9) |
| Barbados | 3 (1-5) | 1.11 (0.4-1.85) | 4 (1-7) | 0.76 (0.21-1.49) | -1.58 (-1.83--1.33) |
| Belarus | 64 (19-121) | 0.49 (0.15-0.92) | 48 (7-130) | 0.3 (0.05-0.82) | -2.82 (-3.21--2.44) |
| Belgium | 64 (12-152) | 0.43 (0.08-1.02) | 69 (7-202) | 0.31 (0.03-0.91) | -1.51 (-1.69--1.33) |
| Belize | 0 (0-0) | 0.01 (0-0.04) | 0 (0-0) | 0.01 (0-0.06) | 0.71 (0.21-1.21) |
| Benin | 16 (6-26) | 0.83 (0.33-1.35) | 46 (15-84) | 0.98 (0.32-1.81) | 0.93 (0.71-1.14) |
| Bermuda | 0 (0-1) | 0.31 (0.02-1.1) | 1 (0-1) | 0.46 (0.1-1.11) | 1.82 (1.55-2.09) |
| Bhutan | 3 (1-5) | 1.23 (0.46-2.09) | 4 (1-9) | 0.81 (0.25-1.59) | -1.4 (-1.53--1.28) |
| Bolivia (Plurinational State of) | 6 (0-20) | 0.2 (0.02-0.66) | 17 (2-48) | 0.2 (0.02-0.58) | 0.48 (0.32-0.64) |
| Bosnia and Herzegovina | 17 (5-31) | 0.43 (0.13-0.78) | 8 (1-25) | 0.13 (0.01-0.43) | -5.33 (-5.76--4.9) |
| Botswana | 21 (9-35) | 3.68 (1.55-6.03) | 44 (17-74) | 3.27 (1.3-5.39) | -1 (-1.42--0.57) |
| Brazil | 424 (63-1197) | 0.48 (0.07-1.35) | 508 (60-1641) | 0.22 (0.03-0.7) | -3.04 (-3.21--2.87) |
| Brunei Darussalam | 1 (0-1) | 0.66 (0.15-1.47) | 1 (0-2) | 0.48 (0.14-0.92) | -1.21 (-1.41--1) |
| Bulgaria | 19 (1-64) | 0.16 (0.01-0.53) | 30 (5-73) | 0.23 (0.04-0.56) | 1.6 (1.37-1.83) |
| Burkina Faso | 46 (23-69) | 1.1 (0.54-1.63) | 127 (60-199) | 1.45 (0.7-2.27) | 1.34 (1.16-1.52) |
| Burundi | 18 (1-71) | 0.77 (0.04-3.01) | 17 (1-66) | 0.37 (0.02-1.43) | -3.44 (-3.92--2.96) |
| Cabo Verde | 5 (2-8) | 2.03 (0.76-3.37) | 11 (3-20) | 2.56 (0.75-4.82) | -0.21 (-0.64-0.22) |
| Cambodia | 38 (16-63) | 0.87 (0.36-1.42) | 76 (30-124) | 0.66 (0.26-1.09) | -1.04 (-1.15--0.92) |
| Cameroon | 8 (1-28) | 0.19 (0.01-0.67) | 38 (3-132) | 0.34 (0.03-1.14) | 1.7 (1.17-2.24) |
| Canada | 80 (7-242) | 0.25 (0.02-0.75) | 170 (17-508) | 0.25 (0.02-0.74) | -0.43 (-0.69--0.17) |
| Central African Republic | 26 (7-55) | 2.19 (0.57-4.61) | 41 (11-81) | 1.91 (0.57-3.75) | -0.5 (-0.55--0.46) |
| Chad | 23 (10-36) | 0.85 (0.38-1.31) | 69 (30-111) | 1.29 (0.57-2.06) | 1.91 (1.69-2.13) |
| Chile | 93 (20-207) | 1.01 (0.22-2.24) | 81 (11-225) | 0.34 (0.04-0.93) | -4.43 (-4.69--4.17) |
| China | 30898 (9680-55564) | 3.86 (1.22-6.92) | 19549 (3277-51906) | 1.02 (0.18-2.68) | -4.99 (-5.56--4.42) |
| Colombia | 49 (4-153) | 0.3 (0.02-0.94) | 51 (4-180) | 0.1 (0.01-0.34) | -4.4 (-4.62--4.17) |
| Comoros | 1 (0-6) | 0.65 (0.03-2.61) | 4 (0-13) | 0.8 (0.06-2.66) | 0.69 (0.57-0.81) |
| Congo | 24 (6-52) | 2.26 (0.56-4.91) | 35 (8-78) | 1.42 (0.32-3.21) | -1.8 (-2.01--1.6) |
| Cook Islands | 0 (0-0) | 0.31 (0.05-0.79) | 0 (0-0) | 0.17 (0.02-0.53) | -2.12 (-2.22--2.01) |
| Costa Rica | 2 (0-8) | 0.13 (0.01-0.46) | 4 (0-13) | 0.07 (0-0.26) | -1.65 (-1.96--1.33) |
| Croatia | 21 (2-59) | 0.32 (0.04-0.9) | 12 (1-41) | 0.15 (0.01-0.49) | -3.33 (-3.57--3.09) |
| Cuba | 25 (2-82) | 0.25 (0.02-0.79) | 39 (3-139) | 0.2 (0.02-0.73) | -0.65 (-1.02--0.28) |
| Cyprus | 0 (0-1) | 0.04 (0-0.17) | 1 (0-4) | 0.07 (0-0.23) | 1.69 (1.45-1.92) |
| Czechia | 38 (6-99) | 0.29 (0.05-0.74) | 69 (13-169) | 0.35 (0.07-0.85) | 1.07 (0.84-1.3) |
| Côte d'Ivoire | 12 (1-37) | 0.3 (0.03-0.96) | 49 (7-140) | 0.48 (0.06-1.38) | 2.16 (1.89-2.43) |
| Democratic People's Republic of Korea | 153 (19-439) | 0.97 (0.12-2.72) | 295 (46-775) | 0.92 (0.14-2.39) | -0.24 (-0.39--0.09) |
| Democratic Republic of the Congo | 154 (18-447) | 1.04 (0.13-2.95) | 515 (154-990) | 1.51 (0.45-2.96) | 0.95 (0.42-1.5) |
| Denmark | 47 (10-108) | 0.6 (0.12-1.36) | 22 (2-76) | 0.19 (0.02-0.66) | -4.69 (-5.45--3.93) |
| Djibouti | 5 (2-9) | 3.78 (1.61-6.22) | 18 (7-33) | 3.1 (1.28-5.52) | -0.93 (-1.07--0.79) |
| Dominica | 0 (0-0) | 0.02 (0.01-0.04) | 0 (0-0) | 0.01 (0.01-0.04) | -0.36 (-0.62--0.09) |
| Dominican Republic | 1 (0-6) | 0.04 (0-0.16) | 2 (0-10) | 0.03 (0-0.11) | -1.47 (-1.79--1.15) |
| Ecuador | 1 (0-4) | 0.02 (0-0.09) | 4 (0-17) | 0.03 (0-0.12) | 0.88 (0.47-1.29) |
| Egypt | 23 (2-74) | 0.08 (0.01-0.25) | 20 (2-87) | 0.03 (0-0.13) | -3.3 (-3.65--2.96) |
| El Salvador | 5 (1-12) | 0.18 (0.04-0.41) | 10 (1-26) | 0.16 (0.02-0.43) | -0.83 (-1.19--0.47) |
| Equatorial Guinea | 4 (1-8) | 1.92 (0.5-3.84) | 2 (0-7) | 0.47 (0.04-1.49) | -6.44 (-7.27--5.6) |
| Eritrea | 29 (9-55) | 2.81 (0.86-5.33) | 59 (17-120) | 2.23 (0.62-4.57) | -0.89 (-0.99--0.79) |
| Estonia | 10 (3-20) | 0.5 (0.15-0.96) | 8 (1-19) | 0.33 (0.06-0.8) | -1.66 (-1.99--1.33) |
| Eswatini | 9 (3-16) | 3.1 (1.03-5.62) | 14 (4-29) | 2.46 (0.65-5.05) | -1.13 (-1.82--0.44) |
| Ethiopia | 264 (122-445) | 1.32 (0.63-2.19) | 310 (143-503) | 0.79 (0.37-1.28) | -2.01 (-2.13--1.9) |
| Fiji | 2 (1-3) | 0.62 (0.26-0.98) | 4 (2-7) | 0.63 (0.23-1.08) | 0.41 (0.08-0.73) |
| Finland | 27 (6-59) | 0.37 (0.08-0.83) | 33 (6-79) | 0.27 (0.05-0.64) | -1.18 (-1.49--0.87) |
| France | 570 (87-1471) | 0.73 (0.11-1.88) | 541 (96-1355) | 0.41 (0.07-1.02) | -2.16 (-2.29--2.03) |
| Gabon | 1 (0-3) | 0.15 (0.02-0.67) | 3 (0-10) | 0.25 (0.02-0.98) | 1.53 (0.97-2.09) |
| Gambia | 2 (1-3) | 0.5 (0.25-0.76) | 6 (3-9) | 0.64 (0.31-0.96) | 0.77 (0.61-0.93) |
| Georgia | 10 (1-29) | 0.17 (0.02-0.48) | 13 (3-30) | 0.23 (0.05-0.52) | 1.78 (1.55-2.01) |
| Germany | 222 (15-737) | 0.18 (0.01-0.61) | 541 (64-1575) | 0.3 (0.04-0.87) | 1.47 (1.1-1.83) |
| Ghana | 15 (2-47) | 0.25 (0.03-0.77) | 13 (1-49) | 0.09 (0.01-0.32) | -3.99 (-4.26--3.72) |
| Greece | 3 (1-12) | 0.02 (0-0.08) | 5 (1-20) | 0.02 (0-0.08) | -1.34 (-1.78--0.9) |
| Greenland | 0 (0-1) | 1.17 (0.16-3.27) | 1 (0-2) | 0.77 (0.08-2.37) | -1.84 (-2.09--1.6) |
| Grenada | 0 (0-1) | 0.25 (0.02-1.01) | 0 (0-1) | 0.26 (0.02-0.97) | 0.51 (0.32-0.7) |
| Guam | 0 (0-0) | 0.16 (0.02-0.47) | 0 (0-1) | 0.14 (0.01-0.44) | -0.23 (-0.64-0.19) |
| Guatemala | 7 (2-16) | 0.23 (0.05-0.5) | 23 (4-61) | 0.22 (0.04-0.57) | -0.34 (-0.76-0.08) |
| Guinea | 3 (0-9) | 0.08 (0.01-0.29) | 7 (1-23) | 0.14 (0.01-0.44) | 2.21 (1.99-2.43) |
| Guinea-Bissau | 3 (1-7) | 0.84 (0.21-1.77) | 7 (2-15) | 0.97 (0.23-2.08) | 1.17 (0.92-1.43) |
| Guyana | 1 (0-2) | 0.31 (0.07-0.67) | 2 (0-4) | 0.3 (0.07-0.64) | 0.23 (0.15-0.32) |
| Haiti | 9 (1-30) | 0.28 (0.02-0.96) | 25 (4-69) | 0.37 (0.06-1) | 0.94 (0.81-1.07) |
| Honduras | 2 (0-6) | 0.1 (0.01-0.3) | 8 (1-25) | 0.15 (0.02-0.44) | 1.78 (1.52-2.04) |
| Hungary | 47 (5-137) | 0.34 (0.04-0.98) | 67 (11-170) | 0.38 (0.06-0.95) | -0.23 (-0.89-0.44) |
| Iceland | 2 (0-4) | 0.63 (0.16-1.31) | 2 (0-5) | 0.36 (0.05-0.96) | -2.21 (-2.4--2.02) |
| India | 5282 (2639-7836) | 1.22 (0.61-1.82) | 10795 (4977-17115) | 0.96 (0.44-1.53) | -1.15 (-1.31--0.98) |
| Indonesia | 537 (213-843) | 0.56 (0.22-0.88) | 629 (159-1377) | 0.31 (0.08-0.69) | -2.09 (-2.2--1.97) |
| Iran (Islamic Republic of) | 37 (4-124) | 0.16 (0.02-0.56) | 55 (8-185) | 0.08 (0.01-0.27) | -2.57 (-3.1--2.02) |
| Iraq | 6 (0-20) | 0.07 (0-0.27) | 29 (5-73) | 0.13 (0.02-0.32) | 1.51 (1.04-1.98) |
| Ireland | 46 (14-89) | 1.13 (0.35-2.18) | 36 (4-106) | 0.48 (0.05-1.39) | -3.55 (-3.91--3.2) |
| Israel | 1 (0-5) | 0.03 (0-0.11) | 3 (0-13) | 0.03 (0-0.11) | -0.03 (-0.25-0.2) |
| Italy | 80 (10-273) | 0.09 (0.01-0.32) | 73 (9-241) | 0.05 (0.01-0.17) | -2.52 (-2.85--2.19) |
| Jamaica | 3 (0-10) | 0.15 (0.01-0.53) | 5 (0-16) | 0.16 (0.01-0.54) | 0.98 (0.63-1.33) |
| Japan | 1121 (320-2293) | 0.66 (0.19-1.35) | 1620 (412-3526) | 0.49 (0.13-1.02) | -0.85 (-1.1--0.6) |
| Jordan | 1 (0-4) | 0.1 (0.01-0.32) | 6 (1-17) | 0.09 (0.01-0.27) | -0.55 (-0.79--0.31) |
| Kazakhstan | 552 (211-854) | 4.55 (1.74-7.09) | 135 (24-337) | 0.85 (0.15-2.08) | -6.44 (-6.7--6.19) |
| Kenya | 135 (44-256) | 1.69 (0.54-3.17) | 409 (122-825) | 1.95 (0.59-3.96) | 0.85 (0.65-1.06) |
| Kiribati | 0 (0-1) | 1.07 (0.21-2.48) | 1 (0-1) | 0.94 (0.15-2.36) | -0.75 (-0.94--0.56) |
| Kuwait | 1 (0-2) | 0.09 (0-0.33) | 2 (0-6) | 0.09 (0.01-0.3) | -0.01 (-0.33-0.31) |
| Kyrgyzstan | 50 (18-81) | 1.65 (0.6-2.7) | 28 (8-56) | 0.68 (0.19-1.33) | -3.66 (-3.87--3.45) |
| Lao People's Democratic Republic | 17 (6-31) | 0.84 (0.3-1.45) | 9 (1-26) | 0.22 (0.03-0.63) | -5.37 (-5.63--5.12) |
| Latvia | 17 (5-31) | 0.46 (0.14-0.88) | 19 (5-38) | 0.53 (0.15-1.03) | 0.43 (0.16-0.7) |
| Lebanon | 0 (0-0) | 0.01 (0-0.02) | 1 (0-4) | 0.02 (0-0.08) | 4.15 (3.75-4.54) |
| Lesotho | 31 (13-49) | 3.16 (1.34-4.97) | 49 (20-81) | 3.88 (1.61-6.39) | 0.98 (0.72-1.24) |
| Liberia | 6 (1-13) | 0.56 (0.13-1.22) | 17 (5-34) | 0.87 (0.26-1.74) | 1.99 (1.67-2.31) |
| Libya | 1 (0-3) | 0.04 (0-0.17) | 3 (0-13) | 0.07 (0-0.25) | 0.68 (0.15-1.22) |
| Lithuania | 18 (4-39) | 0.4 (0.1-0.85) | 23 (4-53) | 0.44 (0.09-1.01) | 0.39 (0.14-0.65) |
| Luxembourg | 2 (0-6) | 0.4 (0.06-1.09) | 3 (0-7) | 0.28 (0.04-0.73) | -1.1 (-1.21--0.98) |
| Madagascar | 94 (24-196) | 1.83 (0.47-3.85) | 190 (59-376) | 1.75 (0.54-3.44) | -0.44 (-0.72--0.15) |
| Malawi | 142 (42-271) | 3.71 (1.09-7.13) | 237 (51-524) | 3.29 (0.72-7.27) | -0.59 (-0.75--0.43) |
| Malaysia | 40 (12-76) | 0.46 (0.13-0.86) | 48 (5-148) | 0.2 (0.02-0.6) | -3.72 (-4.1--3.34) |
| Maldives | 1 (0-1) | 0.68 (0.17-1.41) | 1 (0-2) | 0.26 (0.06-0.55) | -3.99 (-4.28--3.71) |
| Mali | 23 (9-37) | 0.56 (0.21-0.9) | 40 (12-74) | 0.47 (0.15-0.87) | -0.61 (-0.69--0.54) |
| Malta | 1 (0-3) | 0.23 (0.02-0.67) | 1 (0-4) | 0.16 (0.02-0.47) | -0.57 (-0.93--0.21) |
| Marshall Islands | 0 (0-0) | 0.5 (0.14-1) | 0 (0-0) | 0.41 (0.12-0.87) | -0.61 (-0.69--0.53) |
| Mauritania | 12 (5-18) | 1.19 (0.53-1.83) | 23 (10-38) | 1.18 (0.51-1.9) | 0.47 (0.23-0.7) |
| Mauritius | 6 (3-9) | 0.84 (0.38-1.25) | 8 (3-15) | 0.49 (0.17-0.86) | -2.01 (-2.14--1.88) |
| Mexico | 53 (8-152) | 0.13 (0.02-0.39) | 90 (12-267) | 0.08 (0.01-0.23) | -1.92 (-2.25--1.59) |
| Micronesia (Federated States of) | 0 (0-0) | 0.56 (0.15-1.1) | 0 (0-1) | 0.47 (0.13-0.97) | -0.77 (-0.84--0.71) |
| Monaco | 0 (0-0) | 0.11 (0.01-0.43) | 0 (0-0) | 0.07 (0.01-0.32) | -1.04 (-1.27--0.8) |
| Mongolia | 74 (35-112) | 7.85 (3.68-11.98) | 120 (56-188) | 6.59 (2.96-10.4) | -1.05 (-1.31--0.79) |
| Montenegro | 0 (0-2) | 0.07 (0-0.28) | 0 (0-2) | 0.04 (0.01-0.16) | -3.37 (-3.99--2.75) |
| Morocco | 17 (2-48) | 0.13 (0.01-0.37) | 10 (1-41) | 0.04 (0-0.14) | -5.25 (-6.03--4.45) |
| Mozambique | 93 (39-144) | 1.66 (0.7-2.6) | 193 (73-320) | 1.87 (0.7-3.1) | 0.67 (0.54-0.8) |
| Myanmar | 200 (86-340) | 0.87 (0.38-1.46) | 199 (66-364) | 0.44 (0.15-0.81) | -2.55 (-2.63--2.46) |
| Namibia | 4 (2-7) | 0.61 (0.26-0.93) | 9 (4-14) | 0.62 (0.25-1.01) | -0.22 (-0.56-0.12) |
| Nauru | 0 (0-0) | 0.33 (0.05-0.9) | 0 (0-0) | 0.34 (0.06-0.84) | 0.04 (-0.76-0.85) |
| Nepal | 108 (41-179) | 1.15 (0.43-1.92) | 169 (47-334) | 0.8 (0.22-1.56) | -1.19 (-1.56--0.82) |
| Netherlands | 69 (9-196) | 0.35 (0.05-0.99) | 150 (14-465) | 0.44 (0.04-1.36) | 0.71 (0.46-0.96) |
| New Zealand | 11 (1-37) | 0.29 (0.02-0.94) | 19 (2-59) | 0.24 (0.02-0.74) | -0.66 (-0.74--0.58) |
| Nicaragua | 3 (1-5) | 0.22 (0.07-0.38) | 12 (5-19) | 0.29 (0.11-0.48) | 0.36 (-0.04-0.77) |
| Niger | 26 (12-40) | 0.97 (0.46-1.48) | 71 (27-122) | 0.99 (0.38-1.65) | 0.33 (0.18-0.49) |
| Nigeria | 50 (12-120) | 0.12 (0.03-0.28) | 94 (23-222) | 0.12 (0.03-0.28) | 0.05 (-0.1-0.19) |
| Niue | 0 (0-0) | 0.31 (0.07-0.72) | 0 (0-0) | 0.24 (0.04-0.62) | -1.2 (-1.41--1) |
| North Macedonia | 2 (0-6) | 0.09 (0.01-0.3) | 2 (0-8) | 0.07 (0-0.24) | -1.37 (-1.6--1.15) |
| Northern Mariana Islands | 0 (0-0) | 0.1 (0.01-0.29) | 0 (0-0) | 0.2 (0.02-0.58) | 3.72 (3.24-4.2) |
| Norway | 15 (3-38) | 0.23 (0.04-0.57) | 14 (2-39) | 0.14 (0.02-0.4) | -1.85 (-2.03--1.67) |
| Oman | 0 (0-2) | 0.07 (0-0.27) | 1 (0-3) | 0.05 (0.01-0.17) | -1.23 (-1.82--0.63) |
| Pakistan | 737 (254-1319) | 1.31 (0.44-2.34) | 1639 (591-2944) | 1.49 (0.52-2.66) | 0.48 (0.23-0.73) |
| Palau | 0 (0-0) | 0.25 (0.05-0.62) | 0 (0-0) | 0.22 (0.03-0.6) | -0.47 (-0.56--0.38) |
| Palestine | 1 (0-3) | 0.16 (0.03-0.38) | 2 (0-6) | 0.09 (0.01-0.27) | -1.98 (-2.11--1.84) |
| Panama | 3 (0-7) | 0.18 (0.03-0.45) | 6 (1-17) | 0.15 (0.02-0.42) | -0.08 (-0.4-0.24) |
| Papua New Guinea | 5 (2-11) | 0.32 (0.1-0.65) | 13 (3-27) | 0.29 (0.07-0.62) | -0.27 (-0.36--0.18) |
| Paraguay | 2 (0-8) | 0.09 (0.01-0.37) | 10 (1-36) | 0.18 (0.01-0.67) | 2.03 (1.21-2.86) |
| Peru | 27 (6-60) | 0.24 (0.05-0.53) | 33 (4-99) | 0.1 (0.01-0.31) | -3.19 (-3.34--3.03) |
| Philippines | 37 (7-96) | 0.13 (0.02-0.33) | 75 (13-202) | 0.1 (0.02-0.26) | -1.1 (-1.28--0.93) |
| Poland | 246 (76-467) | 0.57 (0.18-1.09) | 254 (58-580) | 0.38 (0.09-0.85) | -1.76 (-1.93--1.59) |
| Portugal | 39 (2-130) | 0.29 (0.02-0.96) | 33 (2-115) | 0.15 (0.01-0.53) | -1.6 (-1.85--1.35) |
| Puerto Rico | 23 (4-60) | 0.65 (0.1-1.67) | 9 (1-31) | 0.12 (0.01-0.42) | -6.9 (-7.45--6.35) |
| Qatar | 0 (0-0) | 0.07 (0.01-0.28) | 0 (0-1) | 0.05 (0.01-0.19) | -2.2 (-2.54--1.85) |
| Republic of Korea | 188 (35-450) | 0.64 (0.12-1.5) | 168 (21-495) | 0.19 (0.02-0.55) | -4.95 (-5.32--4.58) |
| Republic of Moldova | 11 (2-29) | 0.24 (0.04-0.63) | 13 (2-29) | 0.22 (0.04-0.51) | -0.32 (-0.53--0.1) |
| Romania | 41 (6-111) | 0.15 (0.02-0.4) | 59 (5-190) | 0.18 (0.01-0.55) | 0.06 (-0.36-0.48) |
| Russian Federation | 1505 (503-2685) | 0.83 (0.28-1.47) | 883 (193-1958) | 0.38 (0.08-0.83) | -3.37 (-3.63--3.11) |
| Rwanda | 1 (1-2) | 0.04 (0.02-0.06) | 2 (1-2) | 0.03 (0.02-0.04) | -2.61 (-2.94--2.28) |
| Saint Kitts and Nevis | 0 (0-1) | 1.19 (0.49-1.86) | 1 (0-1) | 0.84 (0.3-1.44) | -1.18 (-1.53--0.83) |
| Saint Lucia | 0 (0-0) | 0.12 (0.01-0.52) | 1 (0-2) | 0.29 (0.03-0.91) | 3.59 (3.24-3.94) |
| Saint Vincent and the Grenadines | 0 (0-0) | 0.26 (0.05-0.62) | 0 (0-0) | 0.09 (0.01-0.34) | -4.3 (-4.94--3.66) |
| Samoa | 0 (0-1) | 0.31 (0.08-0.63) | 0 (0-1) | 0.21 (0.05-0.45) | -1.61 (-1.72--1.49) |
| San Marino | 0 (0-0) | 0.09 (0.01-0.27) | 0 (0-0) | 0.08 (0.01-0.26) | -0.58 (-1.03--0.13) |
| Sao Tome and Principe | 0 (0-0) | 0.23 (0.02-0.69) | 0 (0-1) | 0.14 (0.01-0.55) | -2.11 (-2.68--1.53) |
| Saudi Arabia | 4 (0-15) | 0.07 (0-0.28) | 20 (2-62) | 0.12 (0.01-0.36) | 1.52 (0.93-2.12) |
| Senegal | 29 (12-47) | 0.93 (0.39-1.48) | 78 (28-129) | 1.09 (0.4-1.79) | 1.16 (0.82-1.5) |
| Serbia | 16 (1-55) | 0.14 (0.01-0.48) | 12 (1-46) | 0.08 (0.01-0.31) | -2.76 (-3.11--2.4) |
| Seychelles | 0 (0-1) | 0.75 (0.2-1.61) | 1 (0-1) | 0.53 (0.09-1.34) | -1.34 (-1.49--1.2) |
| Sierra Leone | 13 (4-22) | 0.67 (0.22-1.17) | 29 (9-55) | 0.86 (0.26-1.61) | 1.37 (1.14-1.6) |
| Singapore | 8 (1-25) | 0.41 (0.04-1.23) | 5 (0-18) | 0.06 (0-0.25) | -6.54 (-6.93--6.14) |
| Slovakia | 30 (8-62) | 0.51 (0.13-1.05) | 35 (7-82) | 0.39 (0.08-0.91) | -1.34 (-1.59--1.08) |
| Slovenia | 5 (0-17) | 0.2 (0.01-0.71) | 5 (0-16) | 0.11 (0.01-0.39) | -2.44 (-2.96--1.92) |
| Solomon Islands | 1 (0-2) | 0.64 (0.2-1.21) | 2 (0-3) | 0.53 (0.14-1.07) | -0.88 (-1.06--0.69) |
| Somalia | 95 (32-167) | 3.68 (1.26-6.45) | 209 (80-358) | 3.19 (1.22-5.42) | -0.48 (-0.59--0.37) |
| South Africa | 614 (243-1036) | 2.94 (1.15-4.97) | 945 (367-1523) | 2.21 (0.87-3.57) | -1.66 (-2.31--1) |
| South Sudan | 29 (4-81) | 1.25 (0.17-3.49) | 35 (5-99) | 0.93 (0.12-2.67) | -1.31 (-1.43--1.19) |
| Spain | 41 (4-190) | 0.08 (0.01-0.37) | 58 (5-222) | 0.06 (0.01-0.24) | -1.13 (-1.36--0.89) |
| Sri Lanka | 95 (34-163) | 0.93 (0.33-1.61) | 204 (68-385) | 0.82 (0.27-1.54) | 0.2 (-0.33-0.73) |
| Sudan | 49 (8-121) | 0.54 (0.09-1.35) | 83 (10-239) | 0.47 (0.06-1.36) | -0.71 (-0.83--0.58) |
| Suriname | 0 (0-1) | 0.13 (0.02-0.37) | 1 (0-2) | 0.12 (0.01-0.33) | -0.58 (-0.86--0.29) |
| Sweden | 41 (8-99) | 0.27 (0.05-0.65) | 41 (6-119) | 0.19 (0.03-0.54) | -1.6 (-1.76--1.44) |
| Switzerland | 32 (3-99) | 0.31 (0.03-0.98) | 38 (3-117) | 0.22 (0.02-0.67) | -1.28 (-1.32--1.23) |
| Syrian Arab Republic | 1 (0-6) | 0.03 (0-0.12) | 5 (0-19) | 0.05 (0-0.17) | 1 (0.64-1.35) |
| Taiwan (Province of China) | 55 (4-173) | 0.35 (0.03-1.1) | 103 (8-394) | 0.27 (0.02-1.01) | -0.38 (-0.59--0.16) |
| Tajikistan | 60 (19-106) | 2.14 (0.67-3.83) | 56 (16-132) | 1.26 (0.35-3.13) | -1.96 (-2.66--1.26) |
| Thailand | 122 (15-353) | 0.35 (0.04-1.01) | 119 (8-460) | 0.12 (0.01-0.44) | -4.56 (-4.91--4.2) |
| Timor-Leste | 2 (1-3) | 0.66 (0.28-1.03) | 5 (2-8) | 0.6 (0.25-1.03) | -0.35 (-0.57--0.14) |
| Togo | 12 (6-18) | 1.02 (0.47-1.51) | 48 (21-77) | 1.35 (0.59-2.16) | 1.38 (1.21-1.54) |
| Tokelau | 0 (0-0) | 0.36 (0.11-0.71) | 0 (0-0) | 0.23 (0.05-0.54) | -1.66 (-1.72--1.61) |
| Tonga | 0 (0-0) | 0.32 (0.09-0.65) | 0 (0-0) | 0.28 (0.07-0.64) | -0.66 (-0.96--0.36) |
| Trinidad and Tobago | 3 (1-6) | 0.4 (0.12-0.74) | 4 (1-9) | 0.22 (0.05-0.48) | -2.95 (-3.36--2.54) |
| Tunisia | 3 (0-9) | 0.06 (0-0.2) | 4 (0-17) | 0.03 (0-0.14) | -2.15 (-2.46--1.84) |
| Turkey | 14 (1-63) | 0.04 (0-0.17) | 11 (2-46) | 0.01 (0-0.05) | -4.24 (-4.52--3.96) |
| Turkmenistan | 72 (14-169) | 3.84 (0.73-9.03) | 23 (2-76) | 0.61 (0.05-1.99) | -7.05 (-7.64--6.44) |
| Tuvalu | 0 (0-0) | 0.53 (0.17-0.96) | 0 (0-0) | 0.36 (0.1-0.75) | -1.43 (-1.51--1.35) |
| Uganda | 7 (2-27) | 0.12 (0.03-0.45) | 43 (5-164) | 0.3 (0.03-1.19) | 4.07 (3.69-4.45) |
| Ukraine | 388 (114-733) | 0.54 (0.16-1.02) | 323 (90-636) | 0.45 (0.13-0.87) | -1.79 (-2.21--1.36) |
| United Arab Emirates | 0 (0-2) | 0.06 (0.01-0.28) | 14 (1-55) | 0.27 (0.01-1.19) | 5.1 (4.52-5.69) |
| United Kingdom | 981 (310-1848) | 1.07 (0.34-2.03) | 1210 (340-2518) | 0.94 (0.26-1.95) | -0.74 (-0.84--0.63) |
| United Republic of Tanzania | 284 (83-544) | 2.66 (0.78-5.13) | 269 (40-731) | 1.13 (0.18-3.03) | -3.54 (-3.77--3.31) |
| United States of America | 1064 (182-2719) | 0.34 (0.06-0.87) | 1653 (256-4522) | 0.3 (0.05-0.81) | -0.41 (-0.57--0.26) |
| United States Virgin Islands | 0 (0-0) | 0.18 (0.02-0.57) | 0 (0-1) | 0.12 (0.01-0.43) | -1.05 (-1.59--0.51) |
| Uruguay | 38 (6-96) | 0.97 (0.15-2.48) | 23 (2-69) | 0.42 (0.04-1.26) | -2.89 (-3.06--2.72) |
| Uzbekistan | 288 (82-559) | 2.58 (0.73-5.04) | 98 (9-299) | 0.53 (0.05-1.6) | -6.79 (-7.33--6.25) |
| Vanuatu | 0 (0-0) | 0.18 (0.01-0.61) | 0 (0-1) | 0.24 (0.03-0.7) | 1.05 (0.96-1.13) |
| Venezuela (Bolivarian Republic of) | 11 (1-41) | 0.12 (0.01-0.45) | 36 (3-120) | 0.13 (0.01-0.42) | -0.27 (-0.57-0.03) |
| Viet Nam | 162 (51-306) | 0.41 (0.13-0.76) | 288 (45-735) | 0.3 (0.05-0.77) | -0.81 (-0.94--0.67) |
| Yemen | 38 (9-75) | 0.82 (0.2-1.61) | 98 (22-195) | 0.78 (0.18-1.61) | -0.62 (-0.8--0.44) |
| Zambia | 144 (70-225) | 5.14 (2.46-7.88) | 270 (121-431) | 4.1 (1.87-6.49) | -1.19 (-1.45--0.93) |

**Table S6.** The number of DALYs cases and the age-standardized DALYs rate of esophageal cancer attributable to diet low in fruits in 1990 and 2019, and its trends from 1990 to 2019 globally. Abbreviations: DALYs, disability-adjusted life years.

|  | Number of DALYs cases (95% UI) in 1990 | The age-standardized DALYs rate/100000 (95% UI) in 1990 | Number of DALYs cases (95% UI) in 2019 | The age-standardized DALYs rate/100000 (95% UI) in 2019 | EAPC (95% CI) |
| --- | --- | --- | --- | --- | --- |
| Global | 1358518 (473229-2387195) | 32.83 (11.4-57.97) | 1249775 (384470-2595057) | 14.96 (4.6-31.05) | -3.12 (-3.43--2.81) |
| **Sex** |  |  |  |  |  |
| Female | 434380 (160567-761632) | 20.12 (7.43-35.37) | 357301 (128558-668342) | 8.23 (2.98-15.36) | -3.62 (-3.95--3.28) |
| Male | 924139 (307556-1665445) | 46.68 (15.36-83.83) | 892474 (262854-1927948) | 22.31 (6.49-48.36) | -2.9 (-3.2--2.59) |
| **Age** |  |  |  |  |  |
| <24 years | 0 (0-0) | 0 (0-0) | 0 (0-0) | 0 (0-0) | 0 (0-0) |
| 25-29 years | 8894 (4013-14017) | 2.01 (0.91-3.17) | 7870 (3321-12669) | 1.3 (0.55-2.09) | -2.18 (-2.51--1.85) |
| 30-34 years | 16843 (7270-26122) | 4.37 (1.89-6.77) | 15937 (6271-26854) | 2.65 (1.04-4.46) | -2.38 (-2.75--2.01) |
| 35-39 years | 38683 (15404-60885) | 10.97 (4.37-17.26) | 30659 (11836-52124) | 5.67 (2.19-9.64) | -2.64 (-3.01--2.28) |
| 40-44 years | 78244 (29868-131118) | 27.33 (10.43-45.8) | 61231 (22220-108171) | 12.41 (4.5-21.92) | -3.33 (-3.64--3.01) |
| 45-49 years | 109446 (40050-184305) | 47.09 (17.23-79.3) | 105382 (36917-199041) | 22.24 (7.79-42.01) | -3.15 (-3.54--2.76) |
| 50-54 years | 171164 (61757-298613) | 80.51 (29.05-140.46) | 159111 (52448-320541) | 36.43 (12.01-73.38) | -3.25 (-3.69--2.8) |
| 55-59 years | 215896 (72280-387217) | 116.44 (38.98-208.84) | 184827 (57396-379901) | 49.82 (15.47-102.4) | -3.2 (-3.48--2.93) |
| 60-64 years | 223846 (71539-410482) | 139.34 (44.53-255.52) | 179863 (55296-394820) | 57.55 (17.69-126.33) | -3.3 (-3.53--3.07) |
| 65-69 years | 198311 (62365-364771) | 160.59 (50.5-295.39) | 174090 (47620-396102) | 67.32 (18.42-153.18) | -3.38 (-3.67--3.1) |
| 70-74 years | 150568 (47580-279220) | 178.16 (56.3-330.38) | 142150 (37922-322725) | 75.98 (20.27-172.5) | -3.37 (-3.71--3.03) |
| 75-79 years | 90481 (30519-164406) | 147.58 (49.78-268.16) | 92281 (24823-205180) | 72.63 (19.54-161.49) | -3.06 (-3.44--2.68) |
| 80-84 years | 40627 (14107-73203) | 115.35 (40.05-207.85) | 59314 (15825-132429) | 70.26 (18.74-156.87) | -2 (-2.4--1.6) |
| 85-89 years | 12737 (3996-24302) | 84.52 (26.52-161.27) | 28555 (7362-63334) | 65.67 (16.93-145.66) | -1.06 (-1.4--0.72) |
| 90-94 years | 2401 (805-4587) | 54.5 (18.27-104.11) | 7111 (1910-15505) | 42.19 (11.33-91.98) | -1.03 (-1.26--0.8) |
| 95+ years | 377 (122-735) | 36.64 (11.83-71.43) | 1393 (374-3090) | 29.18 (7.84-64.74) | -0.72 (-0.79--0.65) |
| **SDI regions** |  |  |  |  |  |
| High SDI | 119145 (26529-275861) | 11.89 (2.65-27.5) | 145475 (29959-359444) | 8.48 (1.78-20.95) | -1.26 (-1.34--1.18) |
| High-middle SDI | 334042 (103539-621615) | 30.3 (9.41-56.49) | 239523 (54619-587643) | 11.73 (2.69-28.76) | -3.81 (-4.21--3.4) |
| Middle SDI | 640223 (215623-1127963) | 58.85 (19.62-103.77) | 424189 (111095-966353) | 16.53 (4.31-37.74) | -4.83 (-5.32--4.33) |
| Low-middle SDI | 195273 (89117-301896) | 29.64 (13.4-46.02) | 309350 (137100-513684) | 21.21 (9.35-35.36) | -1.36 (-1.47--1.26) |
| Low SDI | 69634 (27137-118521) | 26.39 (10.28-45.16) | 130947 (49730-221231) | 22.76 (8.59-38.53) | -0.64 (-0.75--0.54) |
| **GBD regions** |  |  |  |  |  |
| Africa | 83025 (31409-143409) | 26.37 (9.94-45.7) | 145278 (52874-255677) | 20.88 (7.68-36.96) | -1.14 (-1.39--0.89) |
| African Region | 77298 (29971-131550) | 31.41 (12.09-53.63) | 135105 (49384-238568) | 25.01 (9.11-44.03) | -1.13 (-1.4--0.86) |
| America | 51060 (8195-137075) | 8.41 (1.34-22.62) | 66054 (9909-192781) | 5.28 (0.79-15.34) | -1.71 (-1.85--1.57) |
| Andean Latin America | 837 (171-1966) | 3.9 (0.79-9.23) | 1144 (133-3444) | 2.02 (0.23-6.07) | -2.34 (-2.45--2.24) |
| Asia | 1103098 (405457-1871379) | 51.27 (18.61-87.18) | 929628 (299354-1901799) | 18.75 (5.98-38.54) | -3.87 (-4.25--3.5) |
| Australasia | 2363 (473-5661) | 10.17 (2.04-24.35) | 3557 (527-9454) | 7.68 (1.17-20.36) | -1.07 (-1.12--1.02) |
| Caribbean | 1765 (247-5006) | 6.68 (0.93-18.94) | 2461 (354-7269) | 4.73 (0.69-13.94) | -1.35 (-1.66--1.03) |
| Central Asia | 29548 (10395-50505) | 61.16 (21.62-105.01) | 13435 (3503-30916) | 17.25 (4.6-39.51) | -5.13 (-5.4--4.87) |
| Central Europe | 13019 (3080-29644) | 8.9 (2.13-20.12) | 13675 (2610-33575) | 7.21 (1.41-17.57) | -1.07 (-1.32--0.83) |
| Central Latin America | 3364 (461-9663) | 3.81 (0.51-11.14) | 5551 (814-16298) | 2.31 (0.34-6.8) | -1.89 (-2.03--1.75) |
| Central Sub-Saharan Africa | 8738 (1925-20446) | 33.95 (7.4-80.41) | 19937 (5767-39663) | 33.2 (9.38-66.73) | -0.46 (-0.85--0.07) |
| Commonwealth High Income | 24816 (7485-49982) | 17 (5.15-34.25) | 30395 (7433-68054) | 12.59 (3.11-28.08) | -1.31 (-1.42--1.21) |
| Commonwealth Low Income | 40789 (16732-65692) | 43.03 (17.71-69.22) | 64297 (21916-118584) | 27.17 (9.23-49.76) | -1.63 (-1.69--1.58) |
| Commonwealth Middle Income | 204349 (100526-301015) | 29.64 (14.46-43.58) | 387113 (175205-598855) | 24.2 (10.95-37.44) | -1 (-1.19--0.81) |
| East Asia | 800308 (251143-1433077) | 86.67 (27.29-155.25) | 447377 (72880-1182104) | 21.04 (3.55-55.34) | -5.32 (-5.91--4.73) |
| East Asia & Pacific - WB | 869328 (273945-1556230) | 61.09 (19.2-109.52) | 528419 (96731-1357300) | 16.55 (3.09-42.34) | -4.91 (-5.44--4.38) |
| Eastern Europe | 53488 (17128-96194) | 18.79 (6.06-33.71) | 34712 (8590-74607) | 10.6 (2.68-22.53) | -2.77 (-3.05--2.48) |
| Eastern Mediterranean Region | 31170 (10011-58895) | 15.67 (5-29.69) | 69363 (23829-126999) | 14.05 (4.73-25.86) | -0.44 (-0.61--0.26) |
| Eastern Sub-Saharan Africa | 38841 (15387-66162) | 46.46 (18.13-79.67) | 64945 (23263-118506) | 35.31 (12.53-64.53) | -1.15 (-1.23--1.06) |
| Europe | 120602 (32818-256028) | 11.97 (3.26-25.35) | 108325 (23927-258919) | 7.76 (1.73-18.46) | -1.99 (-2.15--1.83) |
| Europe & Central Asia - WB | 146862 (42790-299814) | 14.13 (4.14-28.77) | 117378 (25956-282746) | 8.11 (1.79-19.37) | -2.44 (-2.61--2.27) |
| European Region | 146938 (42797-300130) | 14.05 (4.11-28.6) | 117507 (26008-283185) | 8.04 (1.78-19.22) | -2.45 (-2.61--2.28) |
| High-income Asia Pacific | 31822 (8712-66560) | 15.36 (4.19-32.11) | 35021 (9216-77203) | 9.11 (2.54-19.36) | -1.72 (-1.95--1.48) |
| High-income North America | 27132 (4605-70020) | 8.29 (1.43-21.37) | 40163 (6054-110370) | 6.89 (1.08-18.68) | -0.63 (-0.76--0.51) |
| Latin America & Caribbean - WB | 24464 (3557-67598) | 8.74 (1.26-24.34) | 26083 (3332-81767) | 3.84 (0.49-12.04) | -3.12 (-3.3--2.94) |
| Middle East & North Africa - WB | 4478 (849-11963) | 3.26 (0.6-8.74) | 8598 (1798-21940) | 2.34 (0.48-6.03) | -1.42 (-1.68--1.16) |
| North Africa and Middle East | 8751 (1863-21568) | 4.62 (1-11.36) | 14959 (3203-37083) | 3.04 (0.63-7.66) | -1.84 (-2.01--1.68) |
| North America | 27123 (4603-70008) | 8.29 (1.43-21.36) | 40161 (6054-110357) | 6.89 (1.08-18.68) | -0.63 (-0.75--0.51) |
| Oceania | 303 (99-568) | 9.05 (2.95-17.1) | 632 (177-1263) | 7.89 (2.24-15.8) | -0.39 (-0.45--0.33) |
| Region of the Americas | 51060 (8195-137075) | 8.41 (1.34-22.62) | 66054 (9909-192781) | 5.28 (0.79-15.34) | -1.71 (-1.85--1.57) |
| South-East Asia Region | 211170 (103393-313497) | 27.07 (13.25-40.17) | 365789 (165673-577784) | 19.96 (9.04-31.62) | -1.26 (-1.38--1.15) |
| South Asia | 199638 (97823-294362) | 31.52 (15.37-46.41) | 376083 (171024-579225) | 25.04 (11.35-38.65) | -1 (-1.12--0.88) |
| South Asia - WB | 204879 (100190-300288) | 31.38 (15.28-46.12) | 385476 (173699-595041) | 24.97 (11.25-38.59) | -0.98 (-1.1--0.86) |
| Southeast Asia | 35663 (13484-60686) | 12.64 (4.77-21.64) | 44196 (11571-94532) | 6.74 (1.76-14.38) | -2.24 (-2.28--2.2) |
| Southern Latin America | 6549 (873-18266) | 14.15 (1.89-39.38) | 3562 (395-11776) | 4.33 (0.48-14.31) | -4.68 (-4.96--4.4) |
| Southern Sub-Saharan Africa | 24086 (10191-38618) | 80.11 (33.9-129.06) | 35712 (15241-55837) | 59.41 (25.25-92.54) | -1.62 (-2.18--1.05) |
| Sub-Saharan Africa - WB | 81136 (31158-138272) | 32.96 (12.6-56.4) | 143315 (52451-251471) | 26.94 (9.95-47.27) | -1.02 (-1.28--0.75) |
| Tropical Latin America | 12013 (1811-33339) | 12 (1.78-33.75) | 13461 (1552-43696) | 5.37 (0.62-17.36) | -3.05 (-3.2--2.9) |
| Western Europe | 52133 (11482-127153) | 9.74 (2.13-23.77) | 58338 (11811-146226) | 7.37 (1.55-18.5) | -1.21 (-1.31--1.11) |
| Western Pacific Region | 839139 (262745-1504830) | 68.11 (21.37-122.26) | 493876 (87469-1284396) | 17.85 (3.26-46.21) | -5.04 (-5.59--4.48) |
| Western Sub-Saharan Africa | 8158 (3256-14512) | 8.68 (3.46-15.44) | 20853 (7780-37679) | 10.2 (3.86-18.36) | 0.84 (0.73-0.96) |
| World Bank High Income | 129517 (29112-301460) | 10.53 (2.36-24.47) | 155459 (31881-390754) | 7.62 (1.6-19.1) | -1.22 (-1.31--1.14) |
| World Bank Low Income | 56405 (20259-102694) | 34.3 (12.25-62.66) | 100982 (34380-183660) | 29.14 (9.83-53.36) | -0.7 (-0.81--0.59) |
| World Bank Lower Middle Income | 271025 (126525-408813) | 23.67 (10.97-35.91) | 469637 (211824-725044) | 18.39 (8.29-28.38) | -1.1 (-1.2--0.99) |
| World Bank Upper Middle Income | 901368 (282393-1613847) | 56.64 (17.76-101.31) | 523407 (95165-1359971) | 15.18 (2.79-39.26) | -5.02 (-5.55--4.47) |
| **Countries** |  |  |  |  |  |
| Afghanistan | 2667 (598-5517) | 35.26 (7.95-72.41) | 4425 (967-9151) | 29.26 (6.45-60.05) | -0.94 (-1.32--0.56) |
| Albania | 114 (18-296) | 5.06 (0.76-13.31) | 23 (3-99) | 0.61 (0.07-2.56) | -8.32 (-8.96--7.67) |
| Algeria | 480 (104-1137) | 3.77 (0.81-8.92) | 344 (23-1208) | 1 (0.07-3.5) | -5.53 (-5.89--5.18) |
| American Samoa | 1 (0-2) | 2.47 (0.4-6.52) | 2 (0-4) | 3.07 (0.53-7.87) | 0.79 (0.62-0.97) |
| Andorra | 3 (0-10) | 5.42 (0.46-16.93) | 6 (0-19) | 4.25 (0.36-13.79) | -1.15 (-1.3--1.01) |
| Angola | 2647 (793-4914) | 58.4 (18.02-108.17) | 2865 (518-7407) | 22.41 (4.15-58.1) | -4.29 (-4.71--3.88) |
| Antigua and Barbuda | 2 (0-6) | 3.23 (0.19-12.32) | 3 (0-12) | 3.18 (0.21-11.38) | 0.35 (0.11-0.59) |
| Argentina | 3767 (271-12050) | 11.67 (0.86-37.19) | 1685 (146-6545) | 3.21 (0.28-12.37) | -5.19 (-5.62--4.76) |
| Armenia | 132 (13-388) | 4.66 (0.47-13.55) | 47 (4-179) | 1.17 (0.09-4.39) | -5.88 (-6.27--5.5) |
| Australia | 2122 (441-4904) | 10.96 (2.29-25.32) | 3179 (498-8347) | 8.16 (1.29-21.25) | -1.13 (-1.18--1.07) |
| Austria | 478 (31-1641) | 4.55 (0.31-15.61) | 314 (22-1253) | 2.06 (0.14-8.08) | -3.42 (-3.67--3.17) |
| Azerbaijan | 1135 (129-3240) | 21.13 (2.36-60.69) | 1006 (64-3822) | 10.01 (0.65-37.51) | -3.78 (-4.29--3.26) |
| Bahamas | 25 (4-65) | 14.79 (2.21-39.13) | 43 (7-121) | 10.15 (1.56-28.08) | -1.72 (-1.86--1.57) |
| Bahrain | 5 (0-19) | 2.58 (0.22-9.54) | 10 (1-36) | 0.86 (0.09-3.11) | -4.63 (-4.96--4.3) |
| Bangladesh | 21945 (10138-33232) | 43.28 (20.33-65.44) | 30895 (11362-54788) | 22.62 (8.28-39.93) | -2.18 (-2.29--2.07) |
| Barbados | 70 (26-116) | 26.31 (9.62-43.42) | 83 (22-164) | 17.41 (4.72-34.46) | -1.66 (-1.91--1.41) |
| Belarus | 1768 (537-3323) | 13.7 (4.18-25.63) | 1297 (183-3474) | 8.5 (1.25-22.67) | -2.86 (-3.27--2.46) |
| Belgium | 1485 (284-3520) | 10.62 (2.08-25) | 1421 (125-4290) | 7.19 (0.63-21.55) | -1.78 (-1.95--1.61) |
| Belize | 0 (0-1) | 0.24 (0.09-0.87) | 1 (0-5) | 0.37 (0.12-1.59) | 1.38 (0.86-1.9) |
| Benin | 415 (161-678) | 20.1 (7.83-32.89) | 1230 (410-2261) | 23.33 (7.63-42.91) | 0.81 (0.6-1.03) |
| Bermuda | 5 (0-16) | 7.15 (0.55-25.43) | 13 (3-31) | 10.78 (2.3-25.37) | 1.95 (1.66-2.23) |
| Bhutan | 87 (32-148) | 31.13 (11.58-53.27) | 111 (33-221) | 18.81 (5.71-37.35) | -1.76 (-1.9--1.62) |
| Bolivia (Plurinational State of) | 149 (13-488) | 4.41 (0.36-14.72) | 380 (42-1084) | 4.22 (0.46-12.12) | 0.25 (0.09-0.41) |
| Bosnia and Herzegovina | 462 (140-835) | 10.39 (3.14-18.71) | 181 (14-608) | 3.18 (0.26-10.46) | -5.34 (-5.78--4.9) |
| Botswana | 579 (237-988) | 93.04 (38.45-156.84) | 1273 (497-2154) | 81.51 (31.9-136.33) | -1.12 (-1.61--0.64) |
| Brazil | 11966 (1806-33135) | 12.24 (1.82-34.37) | 13219 (1530-42930) | 5.4 (0.63-17.46) | -3.11 (-3.27--2.96) |
| Brunei Darussalam | 15 (3-32) | 14.18 (3.15-31.07) | 32 (10-61) | 9.93 (3.02-19.08) | -1.28 (-1.54--1.02) |
| Bulgaria | 523 (35-1717) | 4.39 (0.32-14.16) | 767 (139-1872) | 6.4 (1.25-15.36) | 1.81 (1.56-2.05) |
| Burkina Faso | 1224 (611-1822) | 26.34 (13.1-38.98) | 3427 (1608-5374) | 34.68 (16.43-54.45) | 1.28 (1.1-1.46) |
| Burundi | 536 (25-2066) | 20.73 (0.94-80.93) | 507 (31-2016) | 9.25 (0.58-37.09) | -3.77 (-4.31--3.24) |
| Cabo Verde | 104 (38-172) | 47.69 (17.99-78.25) | 268 (82-504) | 60.99 (18.4-114.8) | 0 (-0.35-0.34) |
| Cambodia | 1107 (457-1855) | 22.24 (9.14-36.93) | 2012 (801-3340) | 15.75 (6.33-25.91) | -1.27 (-1.39--1.16) |
| Cameroon | 224 (14-811) | 4.56 (0.29-16.29) | 1091 (85-3638) | 8.11 (0.62-27.58) | 1.66 (1.08-2.24) |
| Canada | 1792 (153-5484) | 5.65 (0.48-17.28) | 3485 (344-10531) | 5.46 (0.53-16.41) | -0.52 (-0.81--0.22) |
| Central African Republic | 779 (194-1651) | 57.65 (14.78-121.73) | 1265 (357-2471) | 49.12 (13.85-96.53) | -0.6 (-0.65--0.54) |
| Chad | 601 (262-932) | 20.63 (9.04-32.08) | 1862 (806-2981) | 30.98 (13.48-49.69) | 1.84 (1.61-2.06) |
| Chile | 1948 (423-4349) | 19.71 (4.23-43.97) | 1416 (178-4024) | 5.89 (0.76-16.67) | -4.81 (-5.07--4.55) |
| China | 794382 (250428-1420613) | 89.33 (28.23-159.82) | 436575 (71099-1152946) | 21.28 (3.6-55.83) | -5.39 (-5.99--4.79) |
| Colombia | 1190 (106-3682) | 6.51 (0.54-20.4) | 1023 (74-3653) | 1.94 (0.14-6.94) | -4.54 (-4.77--4.32) |
| Comoros | 39 (1-157) | 16.4 (0.67-66.61) | 104 (8-338) | 19.83 (1.46-65.2) | 0.61 (0.46-0.76) |
| Congo | 690 (171-1511) | 58.39 (14.67-129.29) | 1018 (225-2249) | 34.04 (7.51-75.67) | -2.1 (-2.32--1.88) |
| Cook Islands | 1 (0-2) | 7.08 (1.17-18.6) | 1 (0-3) | 3.93 (0.39-12.25) | -2.04 (-2.11--1.97) |
| Costa Rica | 49 (3-176) | 2.69 (0.17-9.85) | 79 (5-298) | 1.54 (0.11-5.79) | -1.64 (-2--1.27) |
| Croatia | 569 (65-1594) | 8.73 (1.03-24.35) | 282 (19-955) | 3.72 (0.26-12.68) | -3.66 (-3.93--3.38) |
| Cuba | 590 (44-1898) | 5.73 (0.42-18.52) | 959 (72-3406) | 5.23 (0.4-18.58) | -0.29 (-0.67-0.1) |
| Cyprus | 7 (0-28) | 0.9 (0.06-3.52) | 28 (2-93) | 1.48 (0.11-4.92) | 1.78 (1.57-2) |
| Czechia | 994 (161-2556) | 7.61 (1.22-19.53) | 1650 (328-4078) | 9 (1.8-21.94) | 0.93 (0.67-1.18) |
| Côte d'Ivoire | 351 (33-1123) | 7.42 (0.66-23.68) | 1413 (194-4047) | 11.51 (1.53-33.27) | 2.05 (1.8-2.31) |
| Democratic People's Republic of Korea | 4395 (545-12450) | 24.55 (3.05-69.76) | 7728 (1228-20391) | 23.16 (3.67-60.58) | -0.27 (-0.41--0.13) |
| Democratic Republic of the Congo | 4488 (568-12654) | 25.42 (3.14-72.43) | 14659 (4412-28214) | 36.68 (10.9-70.42) | 0.92 (0.37-1.47) |
| Denmark | 1078 (234-2433) | 14.79 (3.28-33.18) | 421 (33-1484) | 4.03 (0.32-14.21) | -5.31 (-6.13--4.49) |
| Djibouti | 158 (67-271) | 94.1 (39.69-158.18) | 528 (206-991) | 75.35 (30.04-137.9) | -1.02 (-1.18--0.86) |
| Dominica | 0 (0-0) | 0.33 (0.22-0.69) | 0 (0-1) | 0.32 (0.2-0.71) | -0.14 (-0.46-0.17) |
| Dominican Republic | 38 (3-155) | 0.89 (0.09-3.74) | 65 (10-285) | 0.66 (0.11-2.88) | -1.58 (-1.95--1.2) |
| Ecuador | 27 (5-109) | 0.47 (0.1-1.85) | 94 (10-380) | 0.61 (0.07-2.42) | 0.78 (0.35-1.2) |
| Egypt | 702 (57-2237) | 2.05 (0.15-6.7) | 634 (55-2621) | 0.84 (0.08-3.55) | -3.41 (-3.77--3.04) |
| El Salvador | 126 (27-283) | 4.11 (0.86-9.4) | 208 (32-544) | 3.54 (0.54-9.25) | -0.99 (-1.32--0.67) |
| Equatorial Guinea | 111 (29-231) | 50.26 (13.07-101.84) | 57 (6-183) | 10.64 (1.04-34.21) | -6.91 (-7.78--6.04) |
| Eritrea | 940 (301-1757) | 76.02 (23.64-144.53) | 1837 (552-3642) | 56.96 (16.38-114.98) | -1.1 (-1.19--1) |
| Estonia | 276 (82-534) | 13.6 (4.04-26.12) | 190 (34-459) | 8.65 (1.58-20.6) | -1.96 (-2.29--1.63) |
| Eswatini | 260 (85-468) | 79.26 (25.77-143.56) | 413 (109-874) | 63.8 (16.89-135.12) | -1.08 (-1.83--0.32) |
| Ethiopia | 7897 (3609-13533) | 34.65 (15.89-58.48) | 8238 (3760-13365) | 18.59 (8.57-30.31) | -2.46 (-2.58--2.33) |
| Fiji | 56 (24-90) | 13.99 (6.02-22.72) | 109 (40-192) | 13.8 (5.11-24.26) | 0.34 (0.01-0.66) |
| Finland | 566 (132-1255) | 8.19 (1.92-18.14) | 645 (120-1540) | 6.02 (1.14-14.24) | -1.09 (-1.4--0.77) |
| France | 14236 (2181-36541) | 19.58 (3.07-50.04) | 11454 (2089-28428) | 10.1 (1.89-24.57) | -2.52 (-2.7--2.35) |
| Gabon | 23 (3-96) | 3.88 (0.46-16.69) | 72 (6-283) | 6.1 (0.51-25.02) | 1.37 (0.77-1.98) |
| Gambia | 46 (22-72) | 12.08 (5.9-18.76) | 156 (77-241) | 15.5 (7.52-23.54) | 0.74 (0.55-0.93) |
| Georgia | 280 (30-799) | 4.46 (0.49-12.61) | 331 (71-746) | 6.07 (1.4-13.41) | 1.66 (1.45-1.88) |
| Germany | 5697 (377-19008) | 5.02 (0.35-16.51) | 11869 (1382-34366) | 7.45 (0.9-21.41) | 1.03 (0.7-1.37) |
| Ghana | 430 (49-1292) | 6.19 (0.65-18.76) | 357 (35-1365) | 2.03 (0.21-7.61) | -4.39 (-4.68--4.09) |
| Greece | 57 (15-237) | 0.42 (0.1-1.76) | 91 (13-383) | 0.47 (0.07-2) | -0.56 (-0.98--0.14) |
| Greenland | 12 (2-34) | 30.22 (4.23-83.67) | 14 (1-44) | 18.73 (1.98-57.46) | -1.98 (-2.18--1.78) |
| Grenada | 4 (0-16) | 6.16 (0.4-25.48) | 8 (0-28) | 6.45 (0.42-23.92) | 0.65 (0.46-0.83) |
| Guam | 3 (0-8) | 3.41 (0.35-9.93) | 7 (1-22) | 3.5 (0.38-11.24) | 0.42 (0.03-0.81) |
| Guatemala | 202 (47-431) | 5.14 (1.13-11.09) | 562 (97-1417) | 4.8 (0.81-12.32) | -0.31 (-0.67-0.06) |
| Guinea | 67 (4-244) | 1.93 (0.13-7.03) | 203 (22-627) | 3.41 (0.34-10.69) | 2.34 (2.12-2.56) |
| Guinea-Bissau | 97 (25-204) | 21.73 (5.45-45.76) | 199 (51-424) | 24.29 (5.95-52.46) | 1.06 (0.81-1.31) |
| Guyana | 31 (8-67) | 7.58 (1.82-16.53) | 53 (13-117) | 7.7 (1.89-16.84) | 0.45 (0.35-0.55) |
| Haiti | 253 (18-862) | 7.05 (0.51-24.12) | 707 (117-1906) | 9.1 (1.47-25.03) | 0.88 (0.76-1) |
| Honduras | 52 (6-158) | 2.36 (0.26-7.27) | 202 (23-595) | 3.26 (0.37-9.75) | 1.42 (1.17-1.66) |
| Hungary | 1419 (151-4036) | 10.5 (1.18-29.54) | 1733 (282-4360) | 10.57 (1.69-26.16) | -0.76 (-1.44--0.08) |
| Iceland | 40 (11-83) | 14.96 (3.92-30.79) | 43 (6-115) | 8.55 (1.29-22.58) | -2.25 (-2.48--2.02) |
| India | 154151 (77301-229059) | 30.24 (15.13-44.86) | 292416 (135588-459962) | 24.11 (11.15-38.01) | -1.06 (-1.21--0.92) |
| Indonesia | 15474 (6326-24271) | 14.01 (5.6-21.99) | 16749 (4262-36310) | 7.16 (1.83-15.52) | -2.41 (-2.51--2.31) |
| Iran (Islamic Republic of) | 1036 (117-3559) | 3.66 (0.41-12.42) | 1317 (189-4446) | 1.7 (0.25-5.75) | -2.75 (-3.33--2.16) |
| Iraq | 154 (9-554) | 1.81 (0.1-6.63) | 842 (140-2112) | 3.16 (0.51-8.01) | 1.53 (1.04-2.02) |
| Ireland | 985 (299-1891) | 24.65 (7.49-47.29) | 705 (74-2097) | 9.75 (1.04-29.04) | -3.83 (-4.2--3.46) |
| Israel | 20 (4-87) | 0.45 (0.08-1.93) | 58 (7-241) | 0.53 (0.07-2.22) | 0.53 (0.23-0.84) |
| Italy | 1807 (209-6244) | 2.21 (0.25-7.57) | 1368 (164-4638) | 1.13 (0.13-3.88) | -2.7 (-3.06--2.35) |
| Jamaica | 56 (4-204) | 3.22 (0.2-11.65) | 112 (10-362) | 3.79 (0.33-12.38) | 1.11 (0.75-1.48) |
| Japan | 26238 (7485-53588) | 15.17 (4.35-30.99) | 31488 (8596-66160) | 11.63 (3.47-23.02) | -0.77 (-1.06--0.49) |
| Jordan | 34 (3-109) | 2.25 (0.17-7.41) | 162 (20-457) | 2.16 (0.25-6.13) | -0.54 (-0.78--0.3) |
| Kazakhstan | 13769 (5251-21214) | 105.93 (40.52-164.17) | 3262 (562-8244) | 18.4 (3.31-46.07) | -6.69 (-6.96--6.42) |
| Kenya | 3714 (1211-7030) | 41.41 (13.38-79.16) | 11550 (3490-23192) | 46.56 (13.86-93.64) | 0.71 (0.51-0.91) |
| Kiribati | 12 (3-27) | 27.91 (5.82-64.8) | 19 (3-47) | 23.14 (3.76-58.38) | -0.94 (-1.15--0.73) |
| Kuwait | 15 (1-52) | 2.01 (0.11-7.17) | 54 (5-163) | 1.8 (0.14-5.63) | -0.38 (-0.71--0.05) |
| Kyrgyzstan | 1304 (475-2134) | 41.77 (15.22-68.23) | 702 (195-1391) | 14.96 (4.18-29.61) | -4.22 (-4.46--3.98) |
| Lao People's Democratic Republic | 509 (182-897) | 22.07 (7.91-38.57) | 250 (32-729) | 5.11 (0.64-14.91) | -5.73 (-5.99--5.47) |
| Latvia | 446 (130-850) | 12.64 (3.74-24.06) | 480 (136-929) | 14.52 (4.14-28.15) | 0.27 (-0.01-0.56) |
| Lebanon | 3 (1-10) | 0.14 (0.06-0.43) | 22 (3-93) | 0.42 (0.05-1.75) | 4.87 (4.41-5.33) |
| Lesotho | 832 (355-1339) | 79.85 (34.01-127.28) | 1389 (569-2358) | 100.3 (40.61-167.21) | 1.11 (0.82-1.4) |
| Liberia | 156 (36-338) | 13.47 (3.13-29.38) | 474 (139-941) | 20.66 (6.04-41.45) | 1.9 (1.56-2.23) |
| Libya | 22 (1-83) | 1.09 (0.06-4.06) | 94 (6-350) | 1.6 (0.1-6.2) | 0.56 (0.03-1.1) |
| Lithuania | 499 (125-1063) | 11.19 (2.87-23.65) | 577 (115-1339) | 12.11 (2.57-27.73) | 0.3 (0.01-0.6) |
| Luxembourg | 52 (8-142) | 10.12 (1.56-27.46) | 63 (10-164) | 6.77 (1.06-17.48) | -1.27 (-1.38--1.15) |
| Madagascar | 2748 (739-5669) | 47.85 (12.6-99.78) | 5777 (1809-11510) | 44.25 (13.79-87.32) | -0.54 (-0.81--0.27) |
| Malawi | 4197 (1268-7993) | 96.16 (28.71-183.49) | 6883 (1553-15492) | 83.93 (18.04-187.33) | -0.69 (-0.88--0.5) |
| Malaysia | 1051 (299-1975) | 10.83 (3.09-20.29) | 1165 (115-3613) | 4.25 (0.45-13.08) | -4.05 (-4.53--3.58) |
| Maldives | 18 (5-38) | 17.14 (4.38-36.15) | 18 (5-39) | 5.45 (1.3-11.73) | -4.61 (-4.94--4.29) |
| Mali | 640 (239-1034) | 14.17 (5.27-22.95) | 1099 (345-2099) | 11.66 (3.62-21.93) | -0.72 (-0.81--0.62) |
| Malta | 22 (2-65) | 5.24 (0.53-15.22) | 31 (3-90) | 3.93 (0.42-11.33) | -0.33 (-0.74-0.08) |
| Marshall Islands | 2 (1-4) | 11.98 (3.45-24.07) | 4 (1-9) | 9.91 (2.79-21.57) | -0.64 (-0.72--0.56) |
| Mauritania | 306 (138-475) | 29.22 (13.2-45.29) | 568 (248-940) | 26.45 (11.44-42.84) | 0.16 (-0.08-0.39) |
| Mauritius | 152 (69-224) | 19.62 (8.91-28.91) | 204 (71-360) | 11.41 (3.99-20.07) | -2.06 (-2.19--1.92) |
| Mexico | 1325 (205-3771) | 2.9 (0.44-8.42) | 2200 (307-6510) | 1.82 (0.25-5.41) | -1.69 (-2.03--1.35) |
| Micronesia (Federated States of) | 7 (2-14) | 14.01 (3.89-27.26) | 9 (2-19) | 11.48 (2.98-24.17) | -0.85 (-0.91--0.79) |
| Monaco | 2 (0-6) | 2.73 (0.25-10.47) | 1 (0-6) | 1.78 (0.23-7.48) | -1.12 (-1.32--0.92) |
| Mongolia | 1672 (797-2488) | 162.56 (77.21-243.01) | 2913 (1367-4609) | 130.44 (61.94-202.53) | -1.24 (-1.49--0.98) |
| Montenegro | 13 (1-51) | 1.99 (0.13-7.8) | 9 (1-37) | 1.02 (0.13-4.08) | -3.72 (-4.38--3.06) |
| Morocco | 457 (56-1294) | 3.11 (0.36-8.86) | 264 (26-1039) | 0.8 (0.08-3.17) | -5.59 (-6.36--4.82) |
| Mozambique | 2442 (1058-3852) | 38.45 (16.32-60.25) | 5283 (1984-8741) | 44.85 (16.86-73.78) | 0.85 (0.7-1) |
| Myanmar | 5790 (2402-9854) | 22.63 (9.61-38.22) | 5285 (1792-9570) | 10.67 (3.62-19.54) | -2.85 (-2.94--2.75) |
| Namibia | 118 (50-183) | 15.56 (6.66-24.09) | 241 (98-397) | 15.86 (6.44-26.06) | -0.28 (-0.66-0.1) |
| Nauru | 0 (0-1) | 8.15 (1.28-22.17) | 0 (0-1) | 8.36 (1.5-20.67) | -0.14 (-0.95-0.69) |
| Nepal | 3191 (1223-5318) | 29.86 (11.4-49.54) | 4387 (1248-8611) | 18.79 (5.32-36.64) | -1.52 (-1.92--1.13) |
| Netherlands | 1522 (202-4248) | 8.03 (1.06-22.3) | 3017 (267-9382) | 9.62 (0.87-29.97) | 0.49 (0.2-0.77) |
| New Zealand | 241 (20-775) | 6.23 (0.53-19.92) | 378 (37-1163) | 5.15 (0.52-15.68) | -0.67 (-0.73--0.6) |
| Nicaragua | 79 (26-135) | 4.91 (1.63-8.49) | 263 (103-442) | 5.9 (2.32-9.89) | 0.08 (-0.25-0.4) |
| Niger | 735 (351-1129) | 23.79 (11.28-36.38) | 1907 (722-3265) | 22.86 (8.67-38.98) | 0.1 (-0.06-0.25) |
| Nigeria | 1332 (325-3231) | 2.85 (0.69-6.87) | 2455 (614-5988) | 2.64 (0.65-6.33) | -0.26 (-0.4--0.12) |
| Niue | 0 (0-0) | 7.33 (1.67-17.29) | 0 (0-0) | 5.59 (1.05-14.31) | -1.32 (-1.54--1.11) |
| North Macedonia | 46 (3-155) | 2.32 (0.15-7.76) | 53 (3-194) | 1.7 (0.11-6.04) | -1.57 (-1.8--1.34) |
| Northern Mariana Islands | 1 (0-2) | 2.2 (0.26-6.71) | 3 (0-8) | 4.47 (0.5-12.97) | 3.64 (3.16-4.12) |
| Norway | 322 (62-804) | 5.31 (1.03-13.26) | 274 (40-770) | 3.09 (0.45-8.68) | -2.14 (-2.32--1.96) |
| Oman | 13 (1-51) | 1.62 (0.11-6.63) | 21 (2-77) | 0.94 (0.11-3.64) | -1.75 (-2.24--1.26) |
| Pakistan | 20264 (7120-36096) | 33.32 (11.64-59.86) | 48274 (17356-85358) | 38.02 (13.67-67.75) | 0.46 (0.19-0.73) |
| Palau | 1 (0-2) | 5.9 (1.02-14.38) | 1 (0-3) | 5.37 (0.85-14.26) | -0.4 (-0.5--0.3) |
| Palestine | 33 (6-78) | 3.63 (0.66-8.65) | 55 (8-151) | 2.11 (0.28-5.9) | -2.06 (-2.19--1.93) |
| Panama | 62 (10-152) | 4.02 (0.66-9.99) | 148 (24-404) | 3.57 (0.57-9.76) | 0.09 (-0.27-0.45) |
| Papua New Guinea | 161 (48-322) | 7.69 (2.29-15.52) | 371 (98-769) | 6.82 (1.78-14.47) | -0.35 (-0.44--0.25) |
| Paraguay | 47 (4-191) | 2.02 (0.16-8.27) | 242 (15-870) | 4.22 (0.26-15.25) | 2.15 (1.34-2.96) |
| Peru | 660 (153-1441) | 5.29 (1.18-11.71) | 670 (75-2074) | 2.06 (0.22-6.41) | -3.57 (-3.74--3.4) |
| Philippines | 1115 (209-2869) | 3.2 (0.57-8.29) | 2189 (414-5852) | 2.5 (0.46-6.67) | -1.07 (-1.26--0.88) |
| Poland | 6308 (1967-11886) | 14.66 (4.62-27.51) | 6065 (1383-13835) | 9.61 (2.19-21.76) | -1.77 (-1.97--1.57) |
| Portugal | 913 (56-3076) | 7.02 (0.45-23.24) | 777 (48-2749) | 4.16 (0.26-14.49) | -1.17 (-1.46--0.89) |
| Puerto Rico | 519 (79-1310) | 14.4 (2.15-36.27) | 171 (12-620) | 2.64 (0.18-9.58) | -6.97 (-7.52--6.42) |
| Qatar | 2 (0-8) | 1.39 (0.19-5.39) | 8 (1-33) | 0.75 (0.14-3.15) | -3.08 (-3.42--2.74) |
| Republic of Korea | 5370 (1019-12769) | 15.96 (3.02-37.97) | 3402 (383-10272) | 3.75 (0.44-11.28) | -5.69 (-6.13--5.25) |
| Republic of Moldova | 317 (50-834) | 6.87 (1.1-17.88) | 349 (67-818) | 6.2 (1.22-14.4) | -0.33 (-0.56--0.1) |
| Romania | 1155 (171-3061) | 4.12 (0.63-10.75) | 1596 (127-5036) | 5.12 (0.41-16.07) | 0.18 (-0.29-0.65) |
| Russian Federation | 39284 (13126-69924) | 21.16 (7.14-37.62) | 22577 (5100-49823) | 9.95 (2.32-21.82) | -3.29 (-3.56--3.01) |
| Rwanda | 34 (19-45) | 1.09 (0.61-1.44) | 43 (27-70) | 0.63 (0.41-0.99) | -2.9 (-3.28--2.52) |
| Saint Kitts and Nevis | 10 (4-15) | 28.83 (11.93-44.64) | 15 (5-26) | 20.2 (7.25-35.1) | -1.44 (-1.82--1.06) |
| Saint Lucia | 2 (0-11) | 2.74 (0.27-12.32) | 17 (2-51) | 7.47 (0.77-22.8) | 3.96 (3.58-4.33) |
| Saint Vincent and the Grenadines | 4 (1-11) | 6.36 (1.25-15.36) | 3 (0-12) | 2.26 (0.15-8.4) | -4.45 (-5.07--3.82) |
| Samoa | 7 (2-14) | 7.3 (2.04-15.04) | 8 (2-17) | 5.01 (1.25-10.87) | -1.62 (-1.74--1.5) |
| San Marino | 1 (0-2) | 2.09 (0.19-6.59) | 1 (0-3) | 1.84 (0.14-6.37) | -0.66 (-1.11--0.2) |
| Sao Tome and Principe | 4 (0-11) | 5.6 (0.5-16.9) | 4 (0-15) | 3.03 (0.24-12.56) | -2.53 (-3.14--1.93) |
| Saudi Arabia | 112 (7-429) | 1.65 (0.1-6.31) | 657 (66-1960) | 2.71 (0.25-8.36) | 1.68 (1.13-2.23) |
| Senegal | 770 (320-1245) | 22.48 (9.31-36.32) | 2005 (714-3384) | 25.38 (9.12-42.4) | 1.02 (0.66-1.37) |
| Serbia | 454 (30-1511) | 3.83 (0.27-12.67) | 292 (20-1130) | 2.13 (0.15-8.27) | -2.92 (-3.28--2.55) |
| Seychelles | 11 (3-23) | 19.87 (5.38-41.73) | 16 (3-40) | 13.42 (2.46-33.69) | -1.49 (-1.62--1.36) |
| Sierra Leone | 321 (105-568) | 16.25 (5.26-28.89) | 791 (248-1481) | 20.5 (6.37-38.58) | 1.33 (1.11-1.56) |
| Singapore | 200 (19-585) | 8.83 (0.84-26.15) | 99 (7-393) | 1.25 (0.09-4.94) | -6.81 (-7.18--6.44) |
| Slovakia | 834 (216-1734) | 14.47 (3.77-29.97) | 917 (180-2142) | 10.61 (2.18-24.79) | -1.56 (-1.83--1.29) |
| Slovenia | 128 (6-462) | 5.29 (0.28-18.93) | 106 (7-361) | 2.85 (0.2-9.67) | -2.65 (-3.19--2.11) |
| Solomon Islands | 27 (8-53) | 16.95 (5.16-32.47) | 52 (13-110) | 13.7 (3.53-28.47) | -0.94 (-1.13--0.76) |
| Somalia | 2981 (1009-5345) | 97.06 (32.61-171.61) | 6326 (2458-10854) | 82.21 (31.54-140.43) | -0.59 (-0.7--0.48) |
| South Africa | 17841 (7181-29668) | 77.51 (30.95-130.15) | 24145 (9332-39685) | 51.23 (19.7-82.97) | -2.15 (-2.82--1.48) |
| South Sudan | 818 (119-2293) | 31.57 (4.5-89.75) | 1012 (139-2879) | 22.88 (3.1-64.53) | -1.43 (-1.54--1.31) |
| Spain | 1039 (108-4732) | 2.17 (0.22-9.57) | 1270 (118-4844) | 1.58 (0.15-5.86) | -1.42 (-1.66--1.17) |
| Sri Lanka | 2556 (920-4375) | 21.87 (7.83-37.52) | 4950 (1620-9419) | 18.9 (6.19-35.69) | -0.02 (-0.55-0.52) |
| Sudan | 1337 (236-3350) | 13.33 (2.28-33.59) | 2229 (275-6207) | 10.89 (1.28-31.07) | -0.93 (-1.06--0.81) |
| Suriname | 9 (1-24) | 3.23 (0.44-8.85) | 18 (2-51) | 2.87 (0.36-8) | -0.5 (-0.78--0.23) |
| Sweden | 818 (162-1991) | 5.95 (1.2-14.43) | 762 (98-2203) | 4.05 (0.51-11.64) | -1.75 (-1.91--1.6) |
| Switzerland | 724 (61-2259) | 7.64 (0.66-23.81) | 763 (64-2360) | 4.89 (0.42-15.1) | -1.71 (-1.78--1.65) |
| Syrian Arab Republic | 43 (3-171) | 0.72 (0.04-2.89) | 148 (8-525) | 1.11 (0.06-3.92) | 0.82 (0.44-1.2) |
| Taiwan (Province of China) | 1531 (126-4721) | 8.91 (0.72-27.61) | 3074 (215-12014) | 8.37 (0.57-32.41) | 0.46 (0.18-0.73) |
| Tajikistan | 1607 (501-2871) | 54.72 (17.01-97.19) | 1598 (473-3687) | 28.77 (8.28-67.86) | -2.38 (-3.06--1.71) |
| Thailand | 3509 (467-9973) | 8.74 (1.11-25.08) | 3132 (210-12392) | 3.04 (0.2-11.89) | -4.45 (-4.83--4.06) |
| Timor-Leste | 54 (24-87) | 16.15 (7.03-25.85) | 119 (48-207) | 14.05 (5.68-24.35) | -0.59 (-0.85--0.34) |
| Togo | 337 (154-506) | 24.8 (11.34-37.09) | 1345 (579-2226) | 32.78 (14.28-53.39) | 1.36 (1.19-1.52) |
| Tokelau | 0 (0-0) | 8.42 (2.4-16.38) | 0 (0-0) | 5.24 (1.15-12.32) | -1.81 (-1.89--1.74) |
| Tonga | 4 (1-9) | 7.27 (2.06-14.76) | 5 (1-12) | 6.44 (1.53-14.61) | -0.64 (-0.93--0.34) |
| Trinidad and Tobago | 84 (25-153) | 9.64 (2.9-17.74) | 100 (25-222) | 5.44 (1.34-11.9) | -2.81 (-3.21--2.42) |
| Tunisia | 68 (4-232) | 1.31 (0.08-4.41) | 98 (6-395) | 0.76 (0.05-3.06) | -2.21 (-2.56--1.87) |
| Turkey | 465 (40-2019) | 1.1 (0.1-4.79) | 311 (62-1232) | 0.34 (0.07-1.34) | -4.79 (-5.14--4.45) |
| Turkmenistan | 1953 (388-4537) | 96.14 (18.85-223.74) | 658 (52-2113) | 15.31 (1.16-49.4) | -7.03 (-7.64--6.41) |
| Tuvalu | 1 (0-2) | 12.85 (3.96-23.35) | 1 (0-2) | 8.44 (2.42-17.64) | -1.49 (-1.58--1.39) |
| Uganda | 200 (44-783) | 2.77 (0.65-10.75) | 1304 (133-5032) | 7.68 (0.84-29.73) | 4.42 (4.01-4.83) |
| Ukraine | 10899 (3275-20502) | 15.47 (4.7-29.03) | 9242 (2597-18205) | 13.32 (3.83-26.35) | -1.67 (-2.13--1.21) |
| United Arab Emirates | 14 (1-63) | 1.4 (0.15-6.45) | 552 (21-2206) | 6.53 (0.26-28.72) | 5.28 (4.79-5.77) |
| United Kingdom | 20216 (6542-38285) | 23.72 (7.75-44.74) | 22902 (6463-47378) | 19.81 (5.68-40.91) | -0.92 (-1.02--0.81) |
| United Republic of Tanzania | 7935 (2373-15290) | 66.71 (19.69-128.45) | 7549 (1081-20564) | 27.49 (3.95-74.78) | -3.67 (-3.91--3.43) |
| United States of America | 25327 (4467-64670) | 8.58 (1.53-21.82) | 36663 (5776-99780) | 7.06 (1.14-18.88) | -0.64 (-0.8--0.48) |
| United States Virgin Islands | 4 (0-12) | 4.45 (0.53-13.43) | 5 (1-19) | 3.09 (0.32-11.04) | -0.94 (-1.49--0.38) |
| Uruguay | 833 (127-2136) | 22.04 (3.41-56.15) | 461 (39-1423) | 9.25 (0.77-28.71) | -3.01 (-3.17--2.85) |
| Uzbekistan | 7694 (2247-14705) | 64.75 (18.6-124.89) | 2918 (287-8762) | 12.31 (1.19-37.01) | -7.12 (-7.68--6.56) |
| Vanuatu | 3 (0-10) | 4.26 (0.27-14.13) | 11 (1-32) | 5.85 (0.73-17.18) | 1.13 (1.02-1.24) |
| Venezuela (Bolivarian Republic of) | 280 (18-995) | 2.75 (0.17-9.93) | 866 (70-2860) | 2.9 (0.24-9.53) | -0.08 (-0.39-0.23) |
| Viet Nam | 4268 (1316-7981) | 10.31 (3.18-19.33) | 8051 (1262-20662) | 7.76 (1.21-19.82) | -0.66 (-0.83--0.49) |
| Yemen | 1082 (264-2126) | 19.96 (4.94-39.11) | 2699 (624-5409) | 18.35 (4.18-36.37) | -0.79 (-0.98--0.59) |
| Zambia | 4172 (2023-6432) | 130.36 (62.97-202.45) | 7952 (3514-12890) | 103.62 (46.22-166.27) | -1.25 (-1.53--0.96) |
| Zimbabwe | 4458 (2089-6663) | 102.12 (48.08-151.18) | 8251 (3755-12696) | 106.56 (49.34-164.62) | 0.14 (-0.13-0.41) |

**Table S7.** Changes in deaths numbers of esophageal cancer attributable to diet low in vegetables and fruits based on the population-level determinants and causes between 1990 and 2019.

| Location |  | Dietary risks | | | Diet low in vegetables | | | Diet low in fruits | | |
| --- | --- | --- | --- | --- | --- | --- | --- | --- | --- | --- |
|  | Varname | Overll difference | Value | Contribute to the total changes (%) | Overll difference | Value | Contribute to the total changes (%) | Overll difference | Value | Contribute to the total changes (%) |
| Global | Aging | -3879.13 | 13304.44 | -342.97 | -4415.04 | 3954.40 | -89.57 | -656.26 | 9961.17 | -1517.87 |
|  | Population |  | 41837.86 | -1078.54 |  | 12299.14 | -278.57 |  | 31532.01 | -4804.80 |
|  | Epidemiological change |  | -59021.43 | 1521.51 |  | -20668.58 | 468.14 |  | -42149.44 | 6422.67 |
| High SDI | Aging | 3091.15 | 2282.96 | 73.85 | 1248.55 | 834.56 | 66.84 | 1909.08 | 1531.14 | 80.20 |
|  | Population |  | 2980.67 | 96.43 |  | 1067.75 | 85.52 |  | 2018.35 | 105.72 |
|  | Epidemiological change |  | -2172.48 | -70.28 |  | -653.76 | -52.36 |  | -1640.41 | -85.93 |
| High-middle SDI | Aging | -5022.05 | 3461.50 | -68.93 | -2806.12 | 908.37 | -32.37 | -2762.76 | 2679.20 | -96.98 |
|  | Population |  | 7390.28 | -147.16 |  | 1913.52 | -68.19 |  | 5752.59 | -208.22 |
|  | Epidemiological change |  | -15873.83 | 316.08 |  | -5628.00 | 200.56 |  | -11194.54 | 405.19 |
| Middle SDI | Aging | -11198.91 | 9095.26 | -81.22 | -5872.72 | 2484.50 | -42.31 | -6535.30 | 7028.93 | -107.55 |
|  | Population |  | 21010.70 | -187.61 |  | 5745.90 | -97.84 |  | 16251.77 | -248.68 |
|  | Epidemiological change |  | -41304.87 | 368.83 |  | -14103.12 | 240.15 |  | -29816.00 | 456.23 |
| Low-middle SDI | Aging | 5883.00 | 1892.11 | 32.16 | 1652.60 | 585.81 | 35.45 | 4511.81 | 1430.70 | 31.71 |
|  | Population |  | 8236.67 | 140.01 |  | 2473.37 | 149.67 |  | 6309.09 | 139.83 |
|  | Epidemiological change |  | -4245.78 | -72.17 |  | -1406.58 | -85.11 |  | -3227.97 | -71.54 |
| Low SDI | Aging | 3360.50 | -153.13 | -4.56 | 1358.84 | -66.33 | -4.88 | 2217.32 | -96.63 | -4.36 |
|  | Population |  | 4348.41 | 129.40 |  | 1812.30 | 133.37 |  | 2828.79 | 127.58 |
|  | Epidemiological change |  | -834.77 | -24.84 |  | -387.12 | -28.49 |  | -514.84 | -23.22 |

**Table S8.** Changes in DALYs numbers of esophageal cancer attributable to diet low in vegetables and fruits based on the population-level determinants and causes between 1990 and 2019. Abbreviations: DALYs, disability-adjusted life years.

| Location |  | Dietary risks | | | Diet low in vegetables | | | Diet low in fruits | | |
| --- | --- | --- | --- | --- | --- | --- | --- | --- | --- | --- |
|  | Varname | Overll difference | Value | Contribute to the total changes (%) | Overll difference | Value | Contribute to the total changes (%) | Overll difference | Value | Contribute to the total changes (%) |
| Global | Aging | -207476.14 | 264803.51 | -127.63 | -130686.60 | 78256.47 | -59.88 | -108743.22 | 198954.90 | -182.96 |
|  | Population |  | 1060814.40 | -511.29 |  | 308600.98 | -236.14 |  | 803728.62 | -739.11 |
|  | Epidemiological change |  | -1533094.05 | 738.93 |  | -517544.05 | 396.02 |  | -1111426.74 | 1022.07 |
| High SDI | Aging | 44460.30 | 37283.42 | 83.86 | 18830.49 | 13499.07 | 71.69 | 26330.27 | 25082.98 | 95.26 |
|  | Population |  | 65347.03 | 146.98 |  | 22919.58 | 121.72 |  | 44686.48 | 169.72 |
|  | Epidemiological change |  | -58170.15 | -130.84 |  | -17588.16 | -93.40 |  | -43439.19 | -164.98 |
| High-middle SDI | Aging | -153911.19 | 67146.43 | -43.63 | -73436.32 | 17549.15 | -23.90 | -94518.46 | 52087.45 | -55.11 |
|  | Population |  | 182872.51 | -118.82 |  | 46473.85 | -63.28 |  | 143250.20 | -151.56 |
|  | Epidemiological change |  | -403930.13 | 262.44 |  | -137459.32 | 187.18 |  | -289856.12 | 306.67 |
| Middle SDI | Aging | -339478.25 | 191172.51 | -56.31 | -155004.11 | 52443.90 | -33.83 | -216034.26 | 147625.67 | -68.33 |
|  | Population |  | 529520.39 | -155.98 |  | 144000.97 | -92.90 |  | 410625.84 | -190.07 |
|  | Epidemiological change |  | -1060171.14 | 312.29 |  | -351448.98 | 226.74 |  | -774285.78 | 358.41 |
| Low-middle SDI | Aging | 148543.95 | 35273.02 | 23.75 | 41457.63 | 10774.39 | 25.99 | 114077.74 | 26815.52 | 23.51 |
|  | Population |  | 227165.23 | 152.93 |  | 67275.84 | 162.28 |  | 174991.78 | 153.40 |
|  | Epidemiological change |  | -113894.30 | -76.67 |  | -36592.60 | -88.27 |  | -87729.57 | -76.90 |
| Low SDI | Aging | 92735.54 | -5669.61 | -6.11 | 37375.47 | -2458.56 | -6.58 | 61313.09 | -3578.11 | -5.84 |
|  | Population |  | 123789.34 | 133.49 |  | 51038.68 | 136.56 |  | 81130.45 | 132.32 |
|  | Epidemiological change |  | -25384.20 | -27.37 |  | -11204.65 | -29.98 |  | -16239.25 | -26.49 |


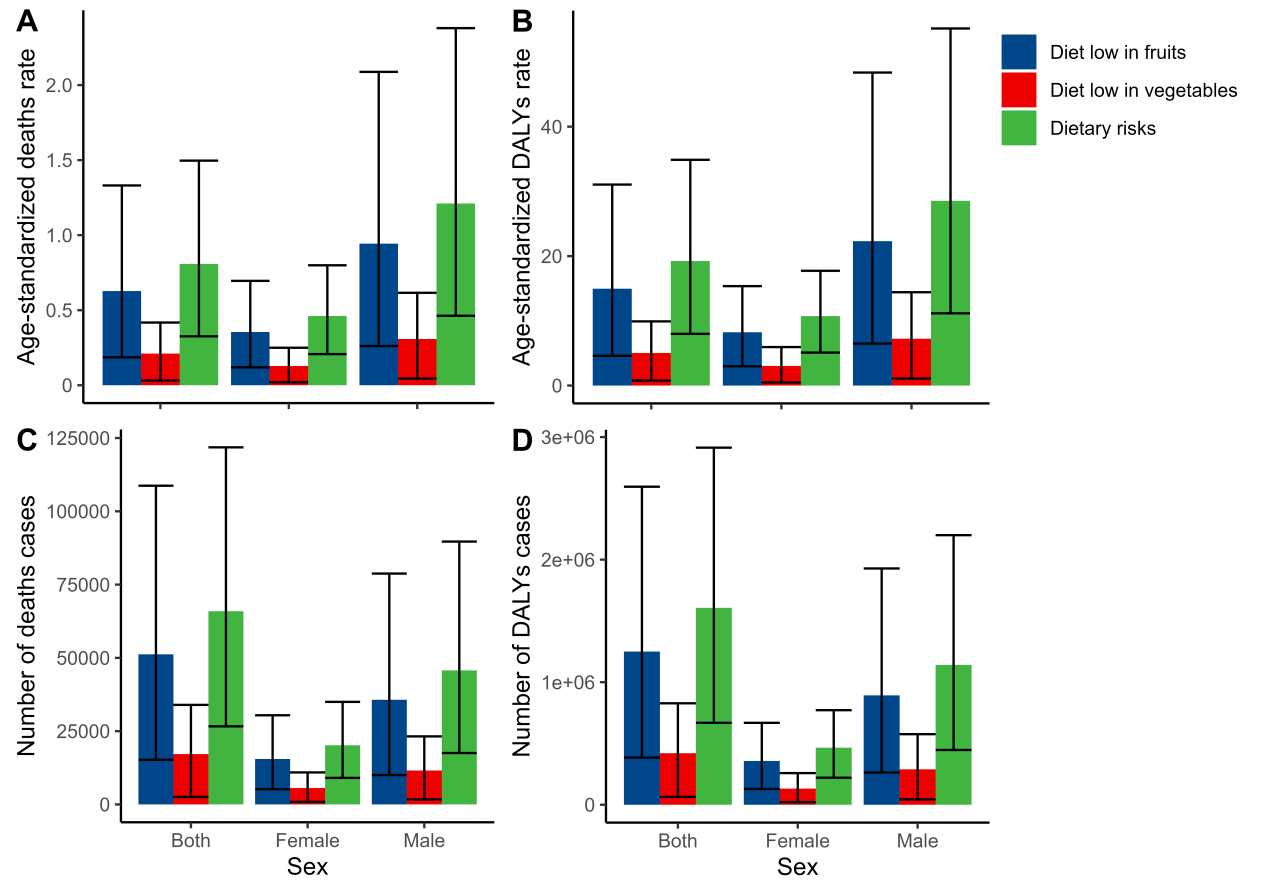


**Figure S1.** Numbers and age-standardized rates of esophageal cancer attributable to diet low in vegetables and fruits deaths and DALYs for different sex in 2019. Abbreviations: DALYs, disability-adjusted life years.


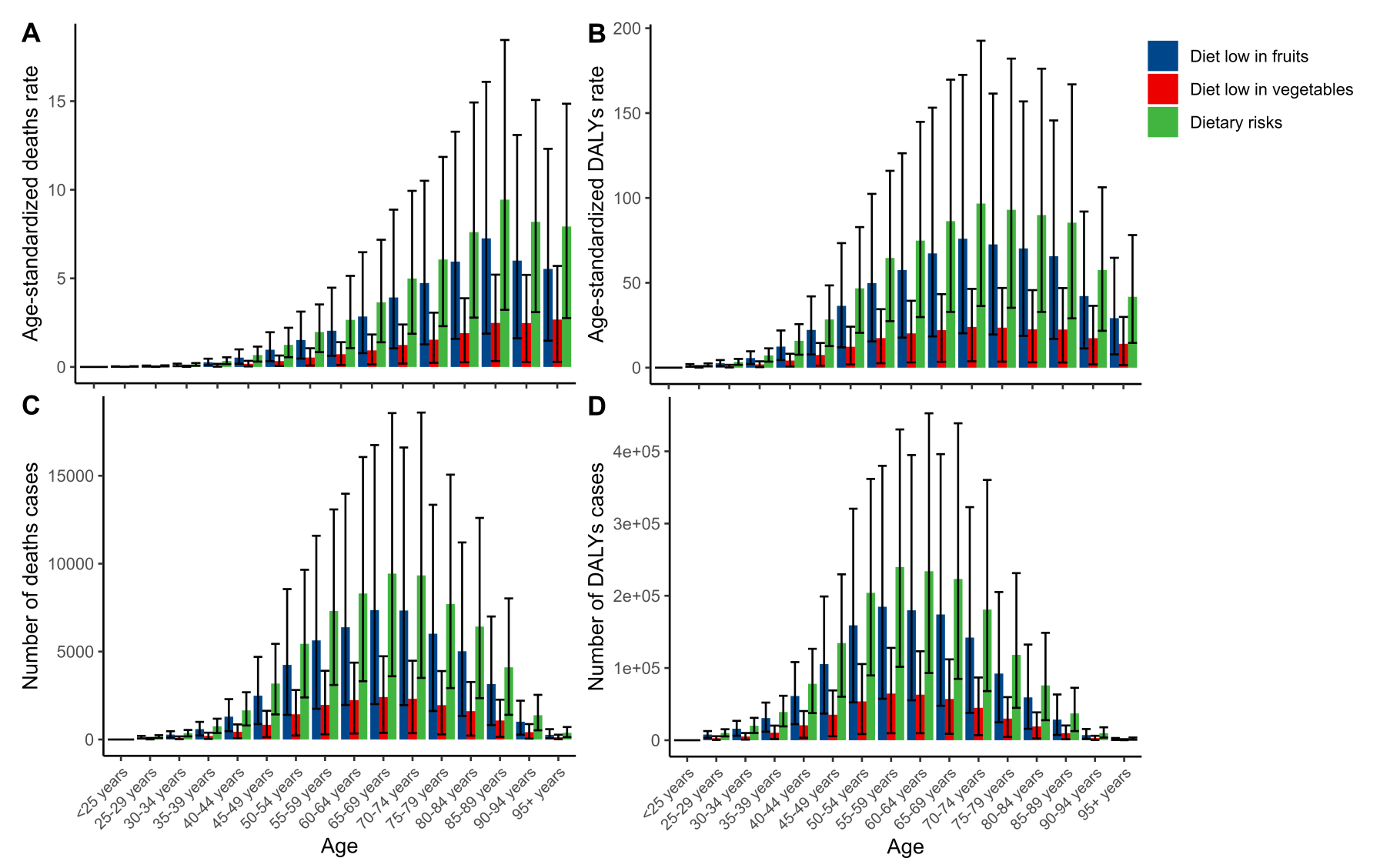


**Figure S2.** Numbers and age-standardized rates of esophageal cancer attributable to diet low in vegetables and fruits deaths and DALYs for different age groups in 2019. Abbreviations: DALYs, disability-adjusted life years.


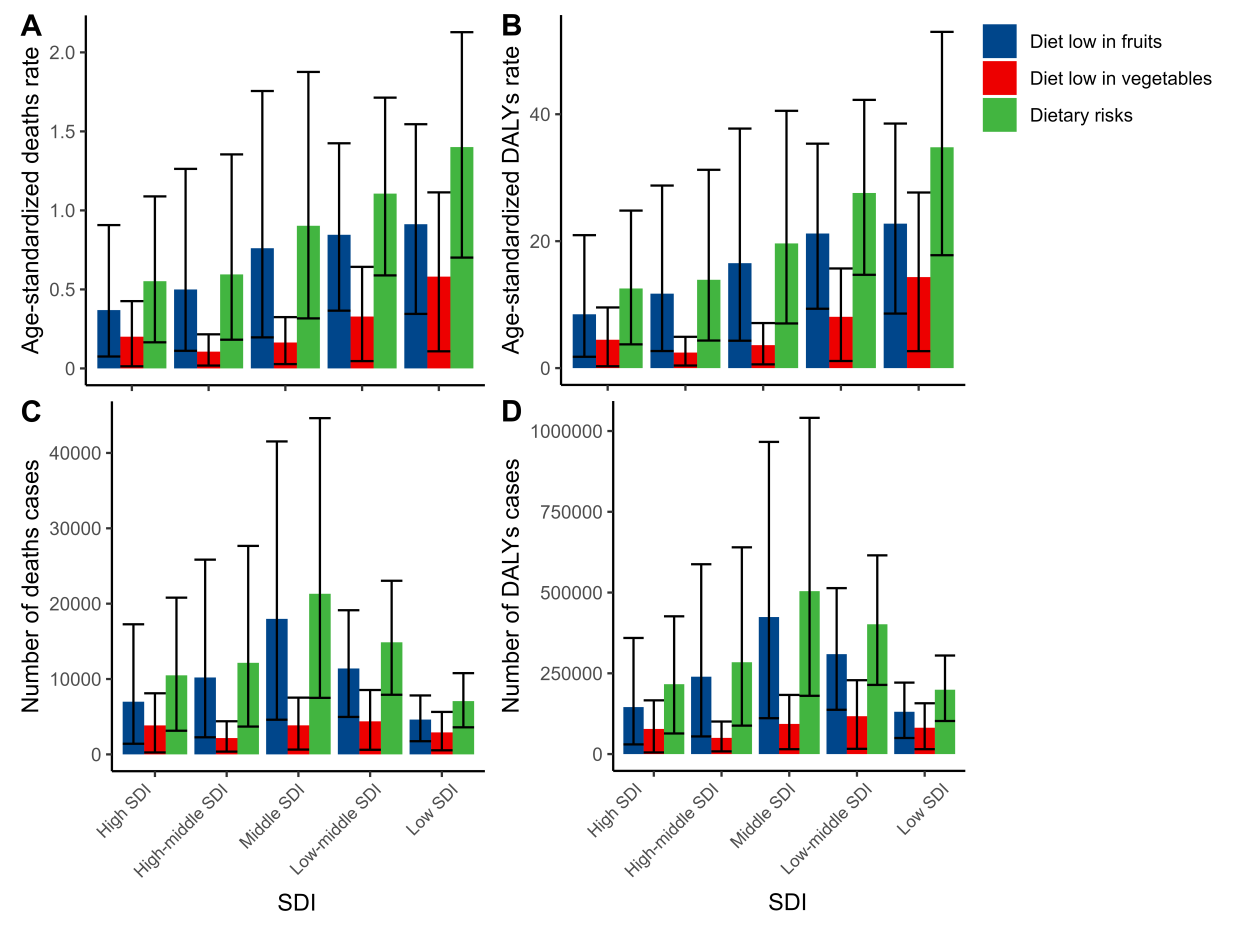


**Figure S3.** Numbers and age-standardized rates of esophageal cancer attributable to diet low in vegetables and fruits deaths and DALYs for different SDI regions in 2019. Abbreviations: DALYs, disability-adjusted life years.


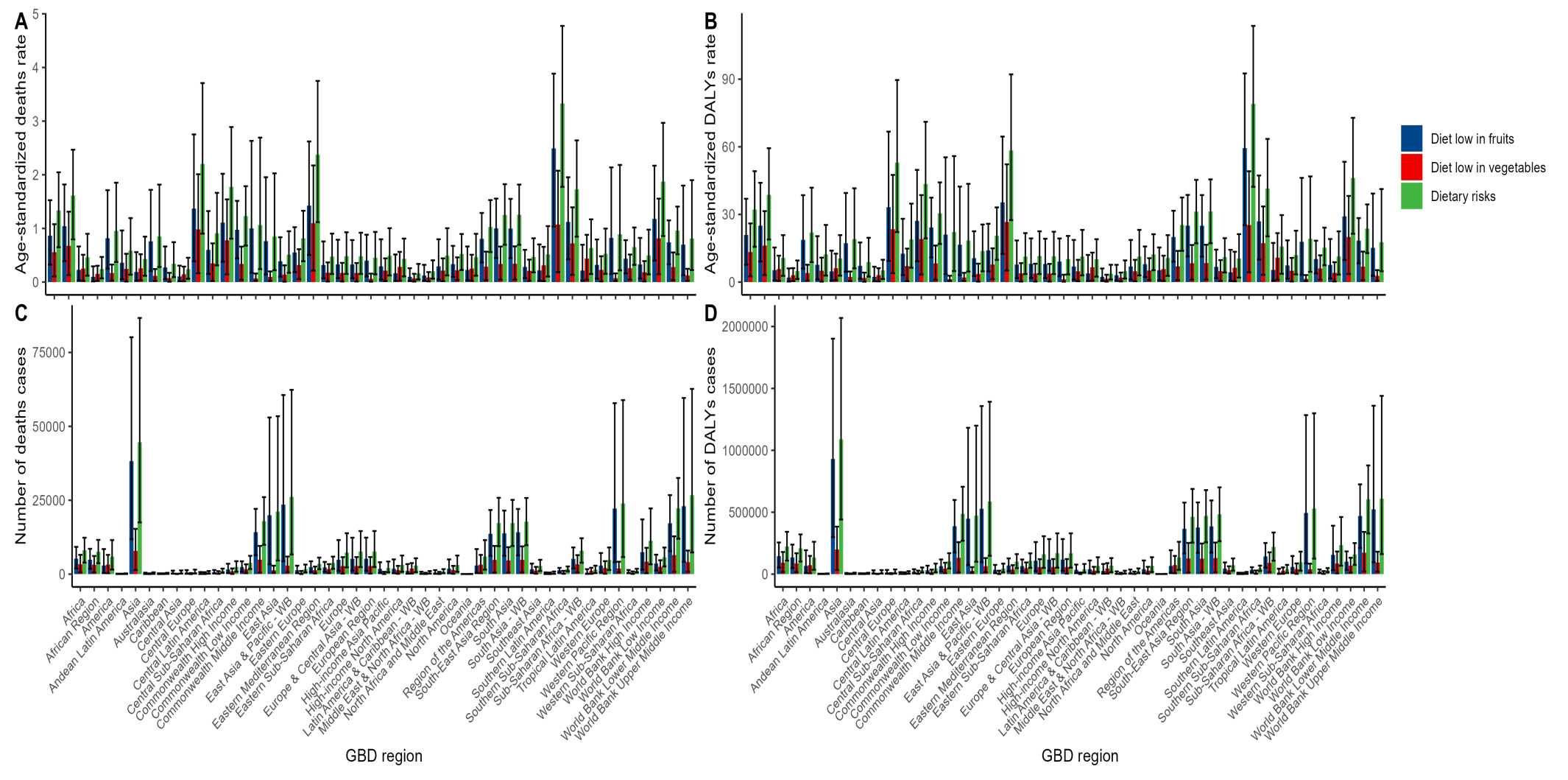


**Figure S4.** Numbers and age-standardized rates of esophageal cancer attributable to diet low in vegetables and fruits deaths and DALYs for different GBD regions in 2019. Abbreviations: DALYs, disability-adjusted life years.


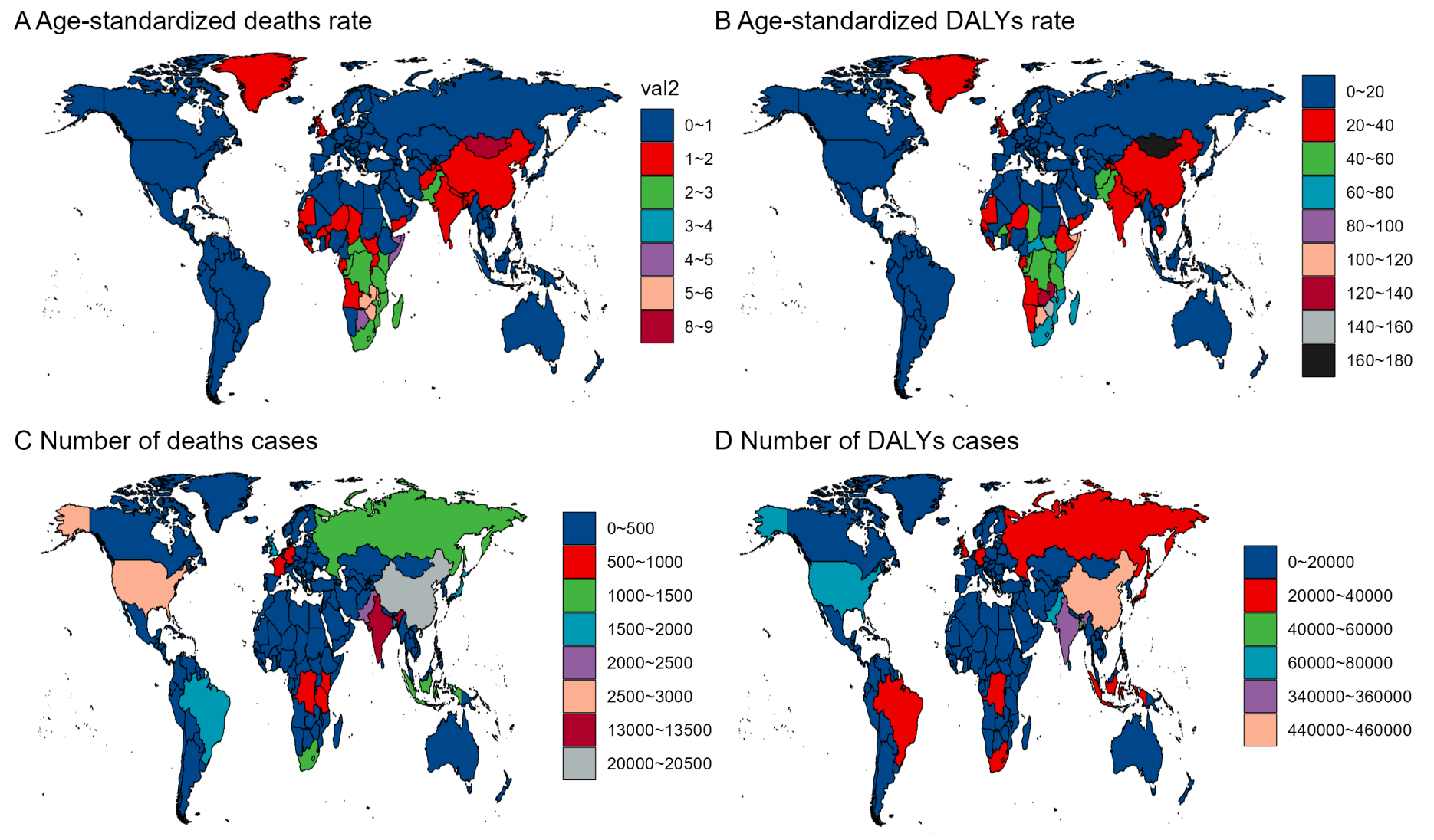


**Figure S5.** Numbers and age-standardized rates of esophageal cancer attributable to dietary risks deaths and DALYs across countries and territories in 2019. Abbreviations: DALYs, disability-adjusted life years.


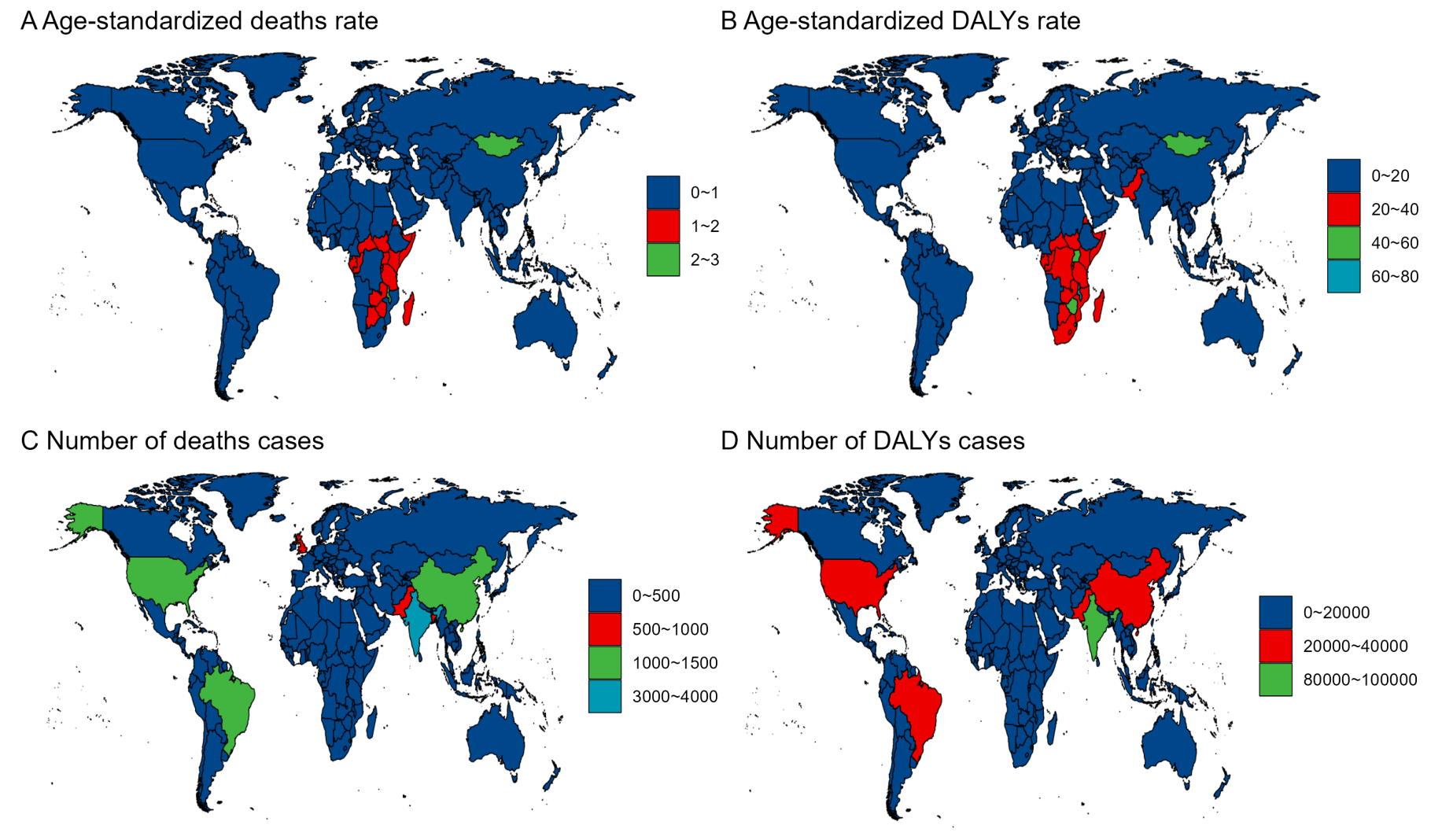


**Figure S6.** Numbers and age-standardized rates of esophageal cancer attributable to diet low in vegetables deaths and DALYs across countries and territories in 2019. Abbreviations: DALYs, disability-adjusted life years.


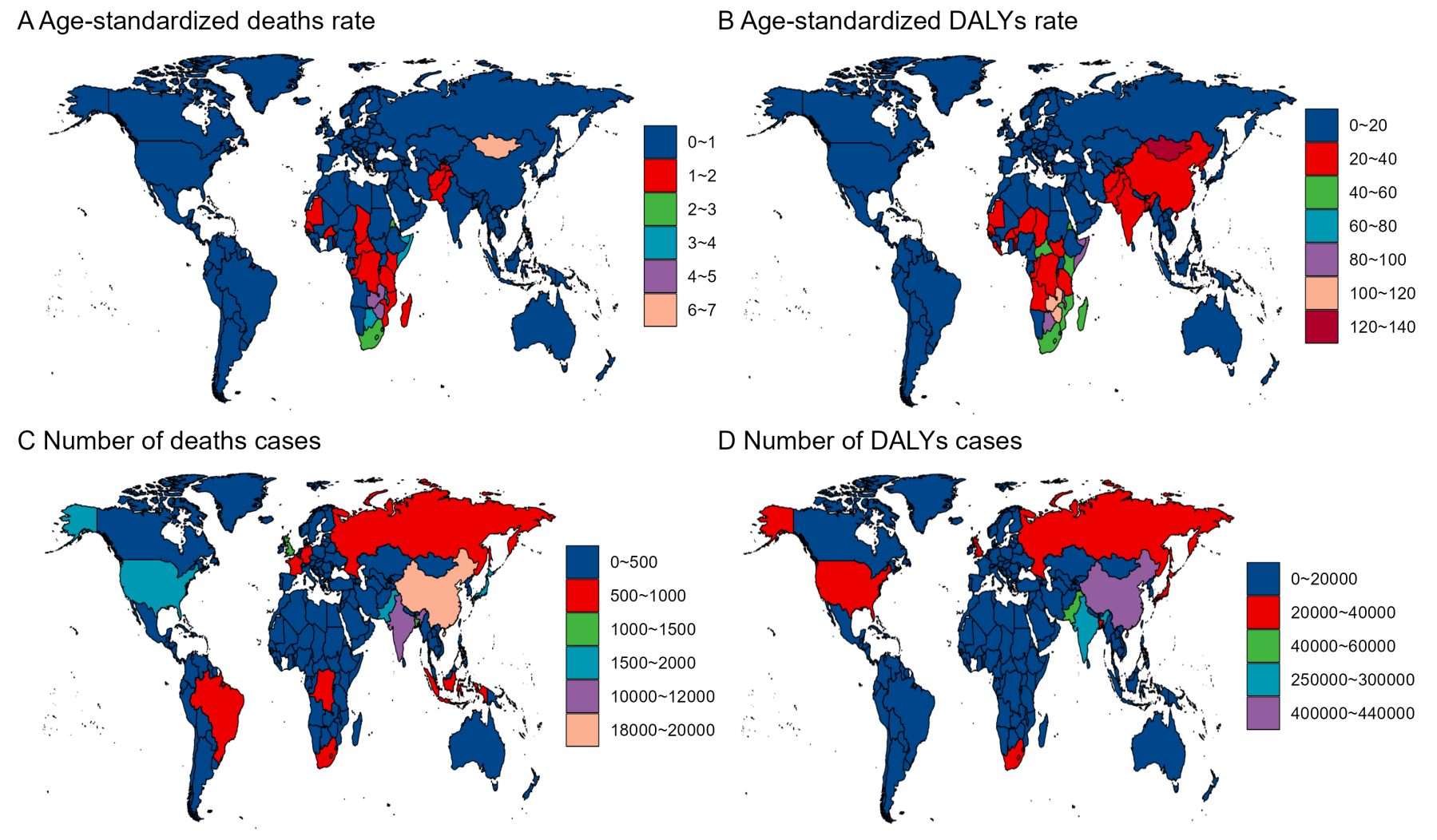


**Figure S7.** Numbers and age-standardized rates of esophageal cancer attributable to diet low in fruits deaths and DALYs across countries and territories in 2019. Abbreviations: DALYs, disability-adjusted life years.


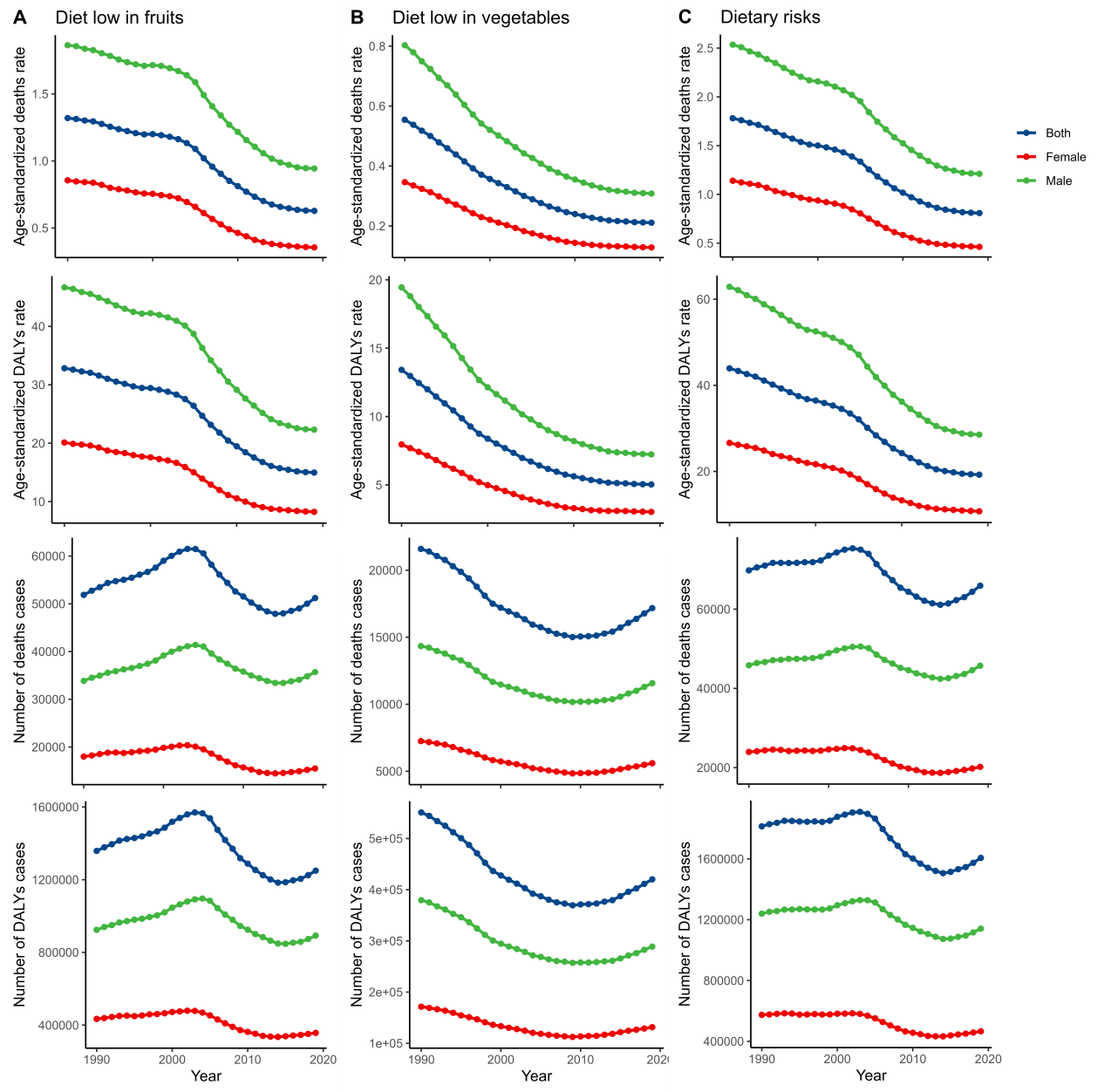


**Figure S8.** Trends in the numbers and age-standardized rates of esophageal cancer attributable to diet low in vegetables and fruits deaths and DALYs globally by sex from 1990 to 2019. Abbreviations: DALYs, disability-adjusted-life-years.


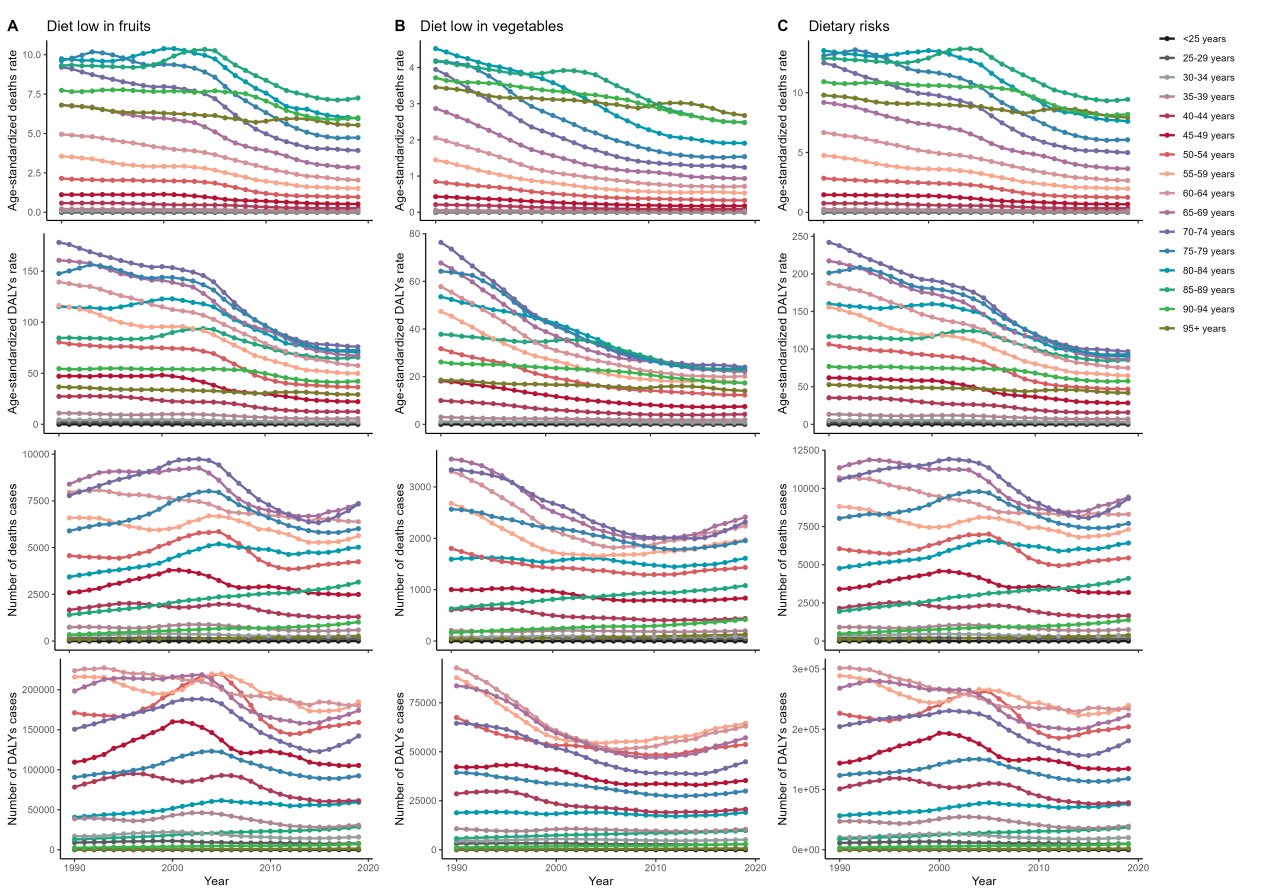


**Figure S9.** Trends in the numbers and age-standardized rates of esophageal cancer attributable to diet low in vegetables and fruits deaths and DALYs globally by age groups from 1990 to 2019. Abbreviations: DALYs, disability-adjusted-life-years.


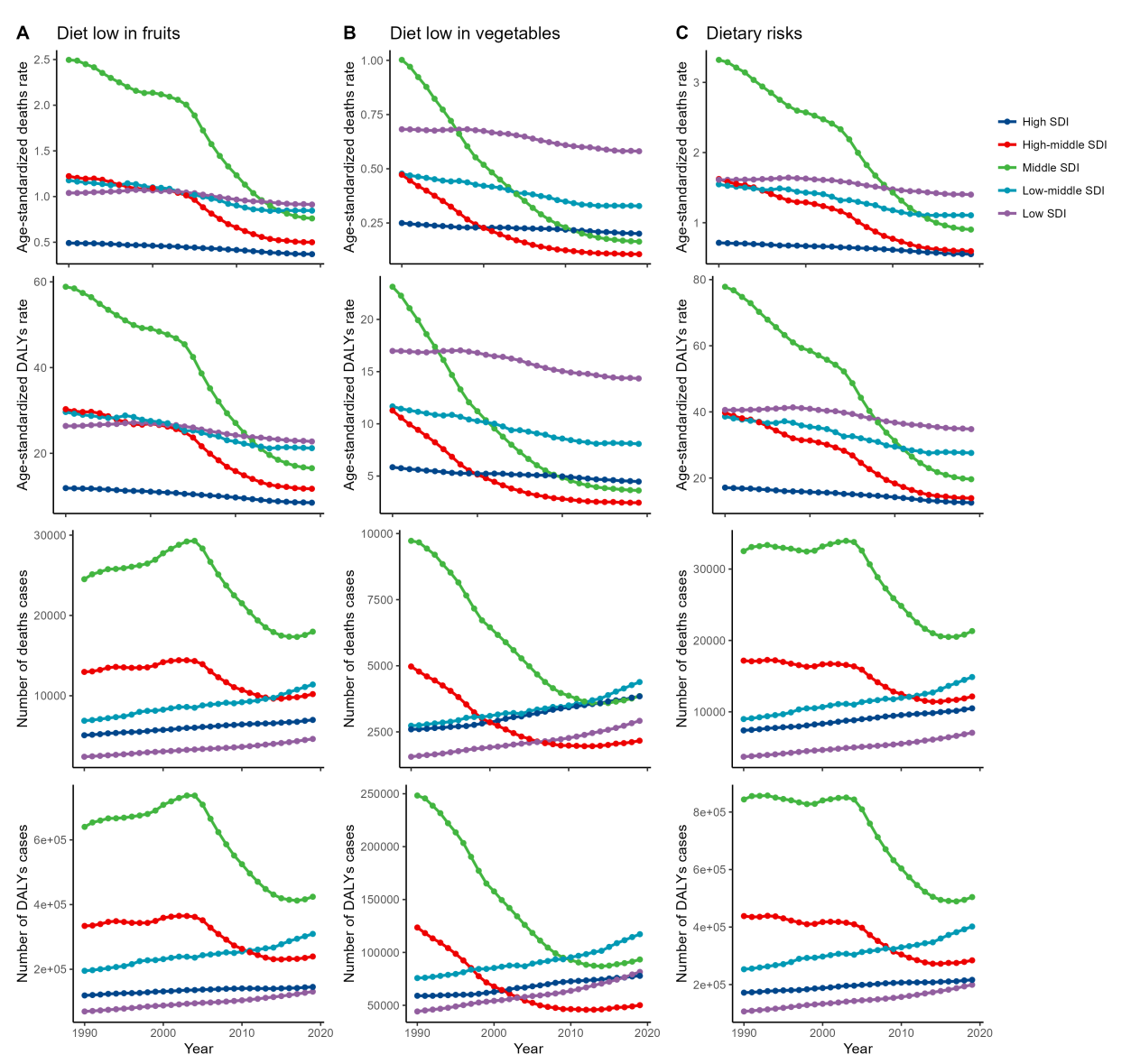


**Figure S10.** Trends in the numbers and age-standardized rates of esophageal cancer attributable to diet low in vegetables and fruits deaths and DALYs globally by SDI regions from 1990 to 2019. Abbreviations: DALYs, disability-adjusted-life-years.


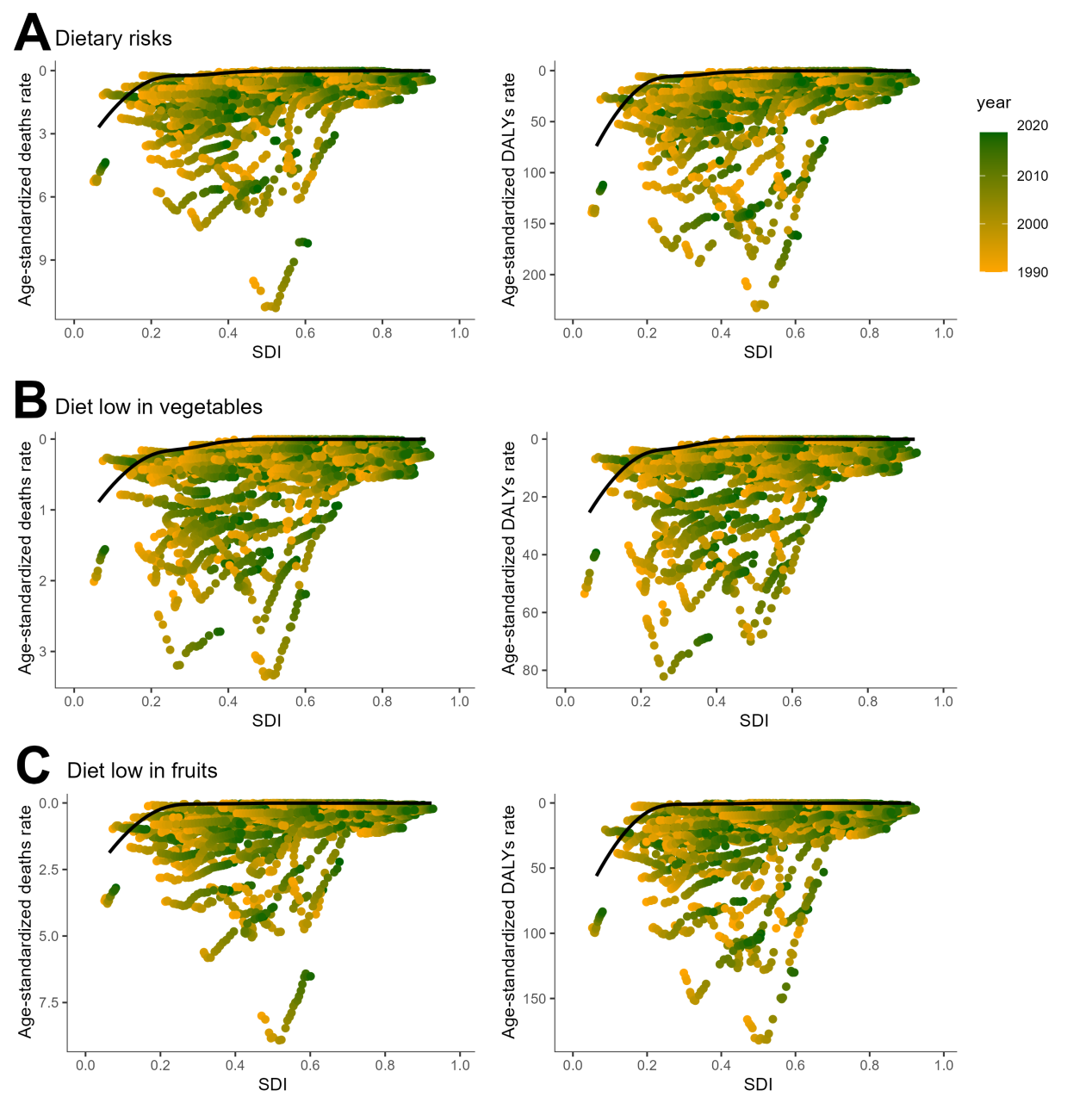


**Figure S11.** Frontier analysis based on SDI and age-standardized diet low in vegetables and fruits-related esophageal cancer deaths and DALYs rate in 2019. The frontier is delineated in solid black color; countries and territories are represented as dots. Abbreviations: SDI, Socio-demographic index; DALYs, Disability-Adjusted Life Years.


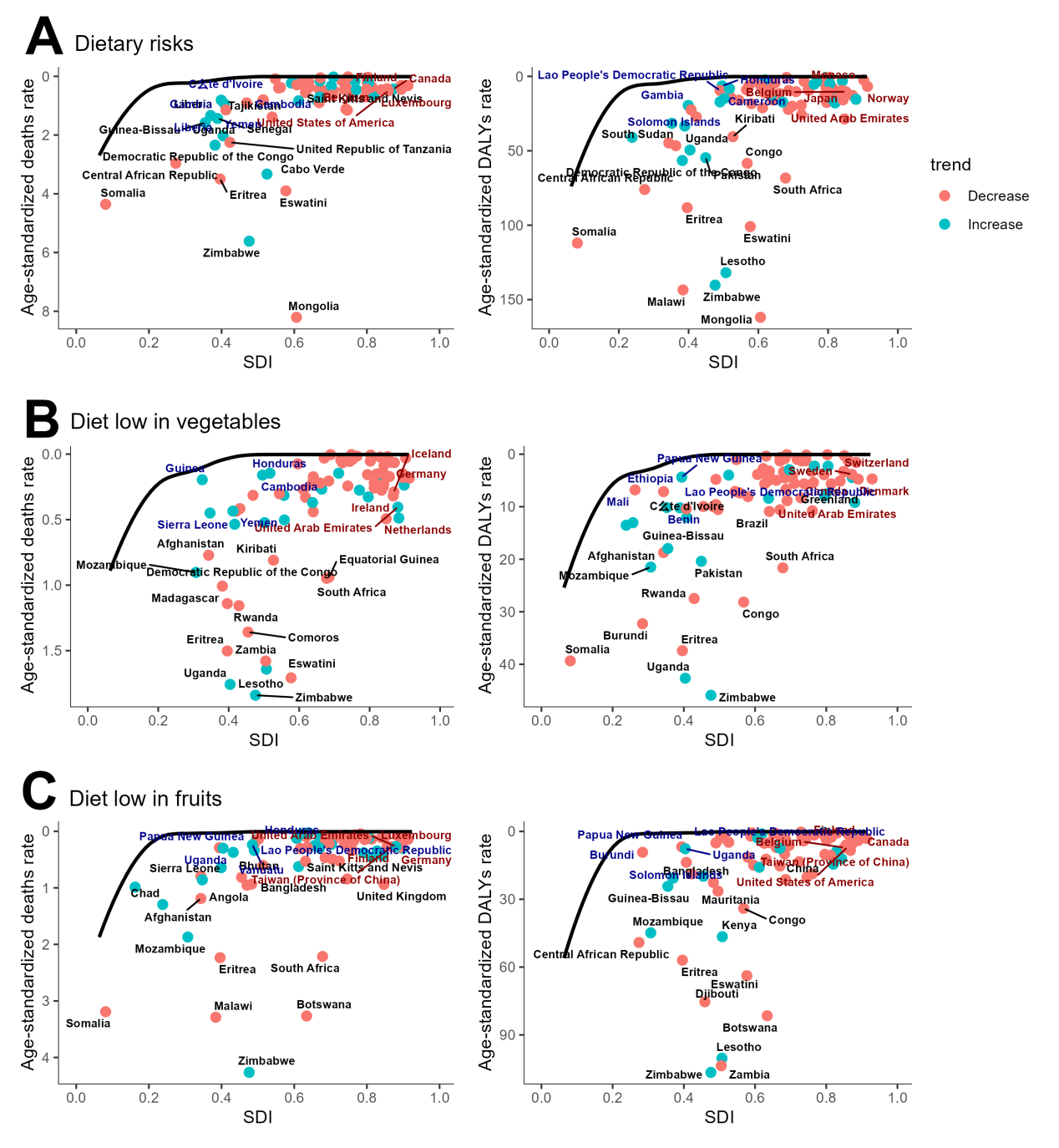


**Figure S12.** Frontier analysis based on SDI and age-standardized diet low in vegetables and fruits-related esophageal cancer deaths and DALYs rate in 2019. The frontier is delineated in solid black color; countries and territories are represented as dots. The top 15 countries with the largest effective difference (largest smoking-related bladder cancer DALYs gap from the frontier) are labeled in black; examples of frontier countries with low SDI (<0.5) and low effective difference are labeled in blue, and examples of countries and territories with high SDI (>0.85) and relatively high effective difference for their level of development are labeled in red. Red dots indicate an increase in age-standardized smoking-related bladder cancer DALYs rate from 1990 to 2019; blue dots indicate a decrease in age-standardized smoking-related bladder cancer DALYs rate between 1990 and 2019. Abbreviations: SDI, Socio-demographic index; DALYs, Disability-Adjusted Life Years.
